# Factors Influencing Breastfeeding Outcomes Following Neonatal Hypoxic Ischaemic Encephalopathy: A Mixed Methods Systematic Review

**DOI:** 10.1177/08903344261426707

**Published:** 2026-04-03

**Authors:** Sarah Edney, Anna Basu, Anne Breaks, Nadia Leake, Judith Rankin, Farag Shuweihdi, Mari Viviers, Kirstin Webster, Lindsay Pennington

**Affiliations:** 1 Newcastle University, Newcastle upon Tyne, UK; 2 Guy’s and St Thomas’ NHS Foundation Trust, London, UK; 3 University of Leeds, UK; 4 Imperial College Healthcare NHS Trust, London, UK; 5 University of Leicester, UK

**Keywords:** breastfeeding, child, feeding, hypoxic ischemic encephalopathy, infant, lactation, mixed methods, neonatal brain injury, systematic reviews

## Abstract

**Background:**

Hypoxic ischaemic encephalopathy (HIE) is the most frequently occurring neonatal brain injury in term-born infants. Families affected by HIE can face significant breastfeeding and lactation challenges.

**Aims:**

This systematic review aims to improve our understanding of these challenges and identify potential solutions by answering the question: What factors influence breastfeeding and lactation outcomes after neonatal HIE?

**Methods:**

This systematic review used a convergent integrated analysis mixed methods design. Eleven databases were searched for qualitative, quantitative, and mixed methods papers meeting specified criteria (e.g., born ≥ 34 weeks gestation) in November 2022 and again in July 2023 and April 2025. Full methods are registered on PROSPERO https://www.crd.york.ac.uk/prospero/display_record.php?ID=CRD42023375506. Of the 3393 titles and abstracts screened, 455 were identified for full-text screening and 10 were included in the review. An additional seven papers were found via reference lists and citation checking, resulting in a total of 17 included studies.

**Results:**

Five categories of potentially influencing factors were identified: infant medical factors, neuroprotective interventions, feeding during therapeutic hypothermia, support for expressing milk, and parent–infant closeness in the neonatal phase. However, significant evidence gaps were demonstrated, including the influence of environmental and social factors, interventions for lactation support and breastfeeding skills, and qualitative and mixed methods studies specifically focused on breastfeeding and lactation following neonatal HIE.

**Conclusion:**

Current research is insufficient to inform our understanding of factors influencing breastfeeding and lactation outcomes following HIE. High-quality breastfeeding and lactation-specific research is urgently needed to inform information sharing and intervention development for HIE-affected families.

## Background

Neonatal hypoxic ischaemic encephalopathy (HIE) is a brain injury resulting from insufficient oxygenation and cerebral blood flow around the time of birth (Inder & Volpe, 2018). In surviving children, long-term neurodisability and health consequences are common (Lee & Glass, 2021; Marlow et al., 2021; Schreglmann et al., 2020).

Supporting direct breastfeeding from the breast and lactation for provision of parent’s milk via tube or bottle feeds may be an important neurodevelopmental intervention following HIE. Lactoferrin, found in high concentrations in colostrum and human milk, has been shown to reduce inflammation and brain injury in animal studies of neonatal brain injury (Sanches et al., 2023; Schirmbeck et al., 2022). In humans, breastfeeding and human milk feeding are associated with improved grey and white matter development, cortical thickness, total brain volume, and improved neurodevelopmental and behavioural outcomes in term and preterm children (Gialeli et al., 2023; Grevet et al., 2024; Janson et al., 2023; Núñez et al., 2023). Additionally, supporting mothers of infants with a history of HIE to breastfeed may help facilitate psychological healing in the context of traumatic birth and having an unwell child (Brown, 2019).

Due to these positive influences on brain development and recovery from brain injury, infants who have experienced HIE may benefit from breastfeeding and human milk feeding, over and above those seen in the general population. However, affected families are likely to face breastfeeding and lactation challenges. Short- and long-term feeding disorders have been identified in infants and children with a history of HIE at all grades (Edney, 2018; Malan et al., 2023; Tsuda et al., 2022), and difficulties specific to breastfeeding have been identified. A case-control study of 28 infants with HIE reported significantly poorer breastfeeding performance compared to healthy infants, including worse outcomes for rooting, latch depth and duration, duration of sucking bursts, and ability to swallow (Krüger et al., 2019). Breastfeeding difficulties have also been observed in mild HIE (Krüger et al., 2017).

In addition to brain injury-related feeding and swallowing disorders, HIE typically requires urgent and often prolonged admission to neonatal care. Therefore, families affected by HIE are also vulnerable to breastfeeding and lactation challenges known to affect other families of hospitalised and medically complex children. These include mother–infant separation; behaviours of the ill infant; lack of privacy; lack of access to appropriate support, expertise, or equipment; milk supply and transfer concerns; hospital feeding policies and practices; staff attitudes; and the psychological effect of the stressful environment and traumatic situation (Alves et al., 2013; Briere et al., 2014; Cescutti-Butler et al., 2019; Dosani et al., 2017; Flacking et al., 2006; Hookway et al., 2021; Ikonen et al., 2015; Magenis et al., 2022; Mӧrelius et al., 2020; Palmquist et al., 2020). Additionally, the social determinants of breastfeeding and lactation outcomes observed in the general population (McKinney et al., 2016; Temple Newhook et al., 2017) have also been evidenced in neonatal settings. In neonatal units, breastfeeding and lactation outcomes have been associated with support from extended family, socioeconomic deprivation, and ethnicity (Elgersma et al., 2023; Liu et al., 2020; Patel et al., 2021).

To characterise, better understand, and identify solutions for specific challenges presented by neonatal HIE, it is essential to identify components that impede or facilitate breastfeeding and lactation in this population. This paper aims to systematically identify, appraise, and summarise currently published literature to answer the question: What factors influence breastfeeding and lactation outcomes after neonatal HIE?

**Key Messages**

• Families affected by neonatal hypoxic ischaemic encephalopathy may be particularly likely to benefit from breastfeeding; however, they are also likely to face challenges. This systematic review aimed to identify factors that influence breastfeeding and lactation outcomes for these families.

• Qualitative studies suggest a need to improve resources for expressing breastmilk and support parent–infant closeness in the neonatal phase. Neuroprotective interventions and enteral feeding during therapeutic hypothermia may improve short-term breastfeeding and lactation outcomes.

• None of the identified interventions targeted skills for breastfeeding, only one study investigated clinical characteristics, and none examined the impact of social factors. Significant evidence gaps need to be addressed to inform parent counselling and intervention development.

## Methods

### Design

The methods used for this convergent integrated analysis mixed methods systematic review followed JBI guidance for mixed methods evidence synthesis (Stern et al., 2020). This paper is part of a larger review on HIE-related feeding outcomes, the protocol for which is registered on PROSPERO https://www.crd.york.ac.uk/prospero/display_record.php?ID=CRD42023375506

### Sample: Defining the Articles Reviewed

Papers were included if they met the following criteria: were peer-reviewed published reports or theses/dissertations of any methodology, and they included participants born at or after 34 completed weeks of gestation, with a primary diagnosis of neonatal HIE and reported quantitative data on factors hypothesised to influence outcomes related to breastfeeding or lactation, or qualitative data regarding factors perceived by parents or healthcare professionals to have influenced these outcomes. Papers were excluded if the cohort included ineligible participants and data for the target population were not reported separately, outcomes were for participants aged ≥ 5 years, or outcomes related only to nutrition/growth. Conference abstracts and quantitative studies in which fewer than three participants met the inclusion criteria (e.g., case studies) were also excluded.

**Figure 1.**
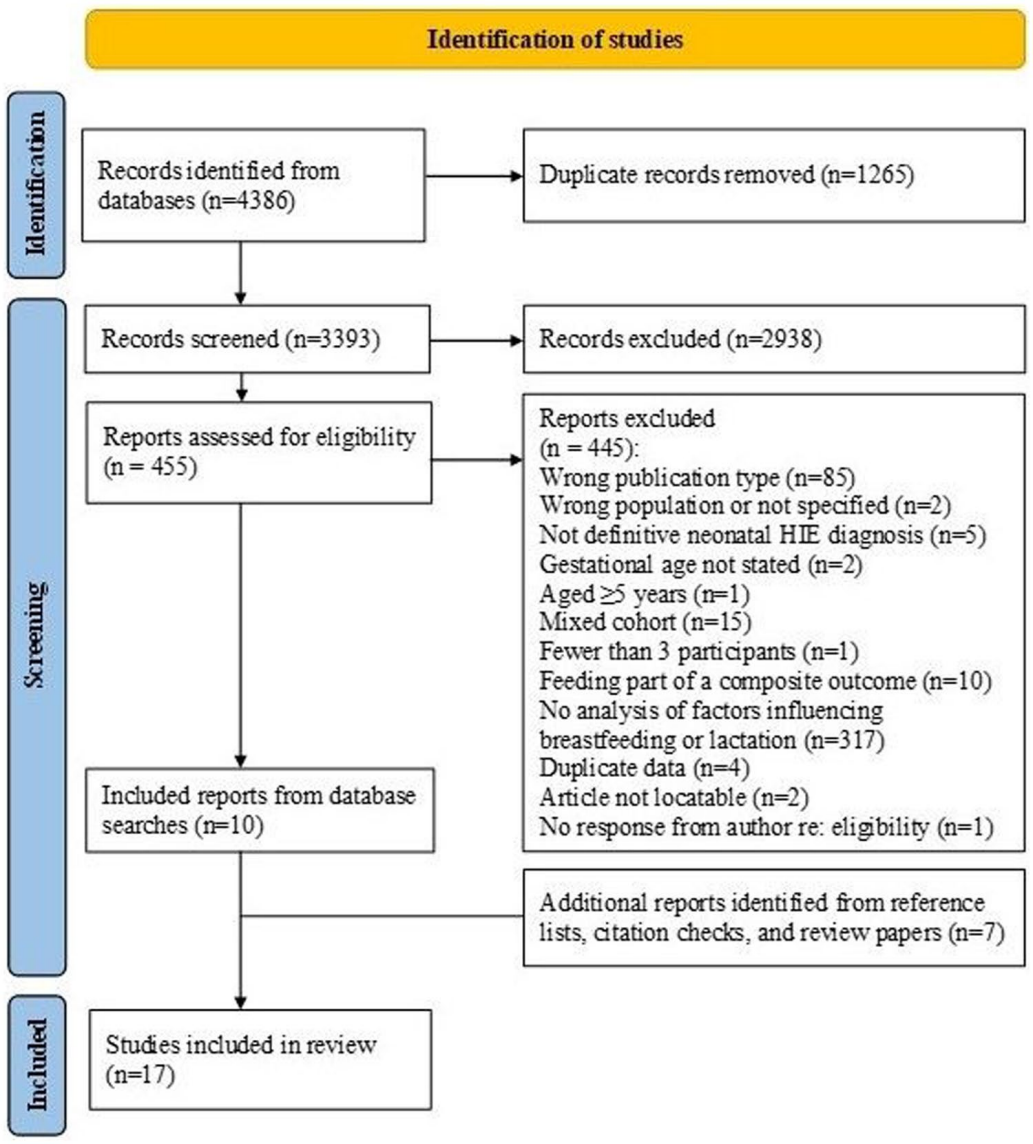
PRISMA diagram: Identification of studies (Adapted from Page et al., 2021 with permission).

Database searches resulted in 3393 unique records. Titles and abstracts were screened by at least two review team members for the original search and July 2024 search re-run. Screening was conducted by a single team member for the April 2025 search re-run. Following exclusion of 2938 records, 455 papers underwent full-text review and screening, with 19% double-screened for quality control. At all stages, conflicts were resolved by consensus. Ten papers from the database searches met the study’s inclusion criteria (see the online Supplementary Material for Table of Excluded Papers), with an additional seven papers identified from 138 review papers and checks of reference lists and subsequent citations of included papers. A total of 17 papers were included in this review (see Figure 1, adapted from Page et al., 2021). No non-English language papers met the inclusion criteria.

### Data Collection: The Search Strategy and Process

A preliminary scoping search was conducted with a university librarian prior to developing the final search strategy, which included terms related to childhood/infancy AND feeding/swallowing AND HIE (see supplementary material for the final Medline strategy). The databases searched were: MEDLINE, Embase, CINAHL, PsycINFO, Scopus, Web of Science, Cochrane Database of Systematic Reviews, Open Access Theses and Dissertations, EThOS, Networked Digital Library of Theses and Dissertations, and ProQuest Dissertation Express. Electronic search strategies are provided in Supplementary Material. Database records were exported into EndNote Version 20.5 (Clarivate, 2020) then, after removing duplicates, were transferred into Rayyan (Ouzzani et al., 2016) and further duplicates were removed. No date or language restrictions were placed on the database searches. Searches were originally carried out in November 2022, then repeated in July 2023 and April 2025 to identify new records dated from January 1, 2022 to March 31, 2025, and a citation check of included papers was conducted in April 2025. Authors were contacted for further details where necessary.

### Measurement

The lead author extracted data from the included papers onto a bespoke MS Excel sheet, previously piloted by the review team. Extracted data included full reference information, the study aim and design, country of origin, setting, sample characteristics, definitions of relevant independent variables, phenomenon and outcomes under study, and relevant results.

Characteristics of included papers are included in the Table. For quantitative studies, variables measured, instruments used, and a summary statement regarding reliability and validity of these instruments are included. Variable definitions have been summarised for brevity.

**Table.** Study Characteristics.

Citation, Setting, and Sample	Study Aims, Design, and Variables Measured	Level of Evidence^a^ and Reliability/Validity
Alburaki et al. (2022). *The Journal of Maternal-Fetal & Neonatal Medicine*. Department of Pediatrics, Alberta Children Hospital, Canada. Sample: 146 infants born in a single NICU at ≥ 35 weeks with moderate to severe HIE and treated with TH.	Aim: Assess effects of EF during TH. Design: Prospective cohort study with historical controls. Intervention: Three hourly EF with human milk, introduced at 10 ml/kg/day during the TH. Volumes increased after rewarming as tolerated until full EF established. Control condition: EF introduced after TH completed and increased by 20 ml/kg/day until full EF established. Outcomes: Type of milk on first feed, rate of excl. BF or BM feeding at discharge and at 4 months.	Level IV – Cohort study. Used routinely collected clinical data with inherent risk of poor data quality. Historical controls, potential differences in practice between the time periods. No statement of strategies to deal with confounding variables. Outcomes not specifically defined.
Bäcke et al. (2021). *Acta Paediatrica*. Department of Women’s and Children’s Health, Uppsala University, Sweden. Sample: eight mothers and three fathers of HIE-affected infants treated with TH in a single NICU.	Aim: Investigate parental experiences of being close to and being involved in the care of their infant during TH. Design: Qualitative study using semi-structured one-to-one interviews and qualitative content analysis.	Level VI – Qualitative study. No stated philosophical perspective or research methodology. No statement locating or regarding the influence of the researcher.
Beasant et al. (2024). *BMJ Open*. Centre for Academic Child Health, University of Bristol, United Kingdom. Sample: 14 neonatal nurses delivering CoolCuddle in four NICUs.	Aims: Examine the normalisation of the CoolCuddle intervention and factors that shape how this intervention may be embedded in tertiary NICUs. Design: Qualitative study using one-to-one and focus group interviews and thematic analysis.	Level VI – Qualitative study. No stated philosophical perspective. No statement locating or regarding the influence of the researcher.
Craig et al. (2020). *The Journal of Maternal-Fetal & Neonatal Medicine.* Department of Pediatric Neurology, Maine Medical Center, United States. Sample: 15 mothers, 12 fathers, and three grandparents of 15 infants treated with TH for HIE in a single NICU.	Aim: Inform improved family-centred care by characterising the emotional experiences of parents of infants treated with therapeutic hypothermia. Design: Exploratory qualitative study using focus group interviews and inductive analysis.	Level VI – Qualitative study. No stated philosophical perspective or research methodology. No statement locating or regarding the influence of the researcher.
Deveci et al. (2025). *The Medical Bulletin of* *Sisli Etfal Hospital*. Division of Neonatology, Harran University, Turkey. Sample: 91 infants born ≥ 35 weeks with moderate to severe HIE and treated with TH in a single NICU.	Aim: Evaluate effect of EF during TH on nutrition-related complications. Design: Retrospective cohort study. Intervention: EF during TH at 10 mg/kg/day, with 30–50 ml/kg/day increments after TH completion. Control condition: EF after TH completion at 30–50 ml/kg/day increments. Outcome: Proportion given nutrition with formula.	Level IV – Cohort study. Used routinely collected clinical data with inherent risk of poor data quality. Confounding factors identified: feeding protocols differed according to individual clinician preference. No statement of strategies to deal with confounding variables. Outcome not specifically defined.
DuPont et al. (2021). *Journal of Perinatology*. University of Utah, United States. Sample: 21 infants born ≥ 34 weeks with mild HIE, not treated with TH in four hospitals.	Aim: Assess the safety and feasibility of using Darbepoetin to treat mild HIE. Design: Randomised-controlled feasibility study. Intervention: Single dose of Darbepoetin intravenously, at 10 μg/kg, within 24 hr of birth. Control condition: Equivalent volume of normal saline within 24 hr of birth. Outcome: Proportion receiving “breast milk” at discharge (not stated if any human milk or only parent’s milk).	Level II – RCT. RoB 2 risk of bias judgement: Some concerns. No pre-specified analysis plan stated. Outcome not specifically defined.
Gale et al. (2021). *Health Technology Assessment*. Neonatal Medicine, Imperial College, United Kingdom Sample: 5716 infants born ≥ 36 weeks with HIE and treated with TH in all NHS neonatal units in England, Wales and Scotland.	Aim: Assess the association between EF and PN during TH and clinical outcomes. Design: Retrospective, population-based cohort study with propensity score matching. Interventions: (1) Any milk feed on at least 1 day during TH; (2) any PN on at least 1 day during TH. Control conditions: (1) No EF recorded in first 3 days after birth and at least 1 day recorded as no EF; (2) No PN recorded in the first 3 days after birth and at least 1 day recorded as receiving intravenous dextrose. Outcomes: Time to first feed of parent’s own milk (1st day recorded to be receiving parent’s own milk by any route), onset of breastfeeding (1st day recorded to be suckling at the breast), breastfeeding at discharge (any suckling at the breast at discharge).	Level IV – Cohort study. Used routinely collected clinical data with inherent risk of poor data quality.
Hu et al. (2022). *Italian Journal of Pediatrics*. Department of Neonatology, Children’s Hospital of Chongqing Medical University, China. Sample: 80 infants born ≥ 35 weeks with moderate to severe HIE and treated with TH in a single NICU.	Aim: Evaluate the effect of EF during TH on clinical outcomes. Design: Single-centre, non-blinded, parallel-group RCT. Intervention: Three hourly EF initiated at 10–20 ml/kg/day during TH and rewarming, increased after 3 days of EFs by 10–20 ml/kg/day until full EF established (120–160 ml/kg/day). Control condition: Feed initiation and progression protocol as above, initiated after completion of TH. Outcomes: Feed type at initiation and at discharge: breastfeeding (including exclusive or mixed) vs. pure formula feeding.	Level II – RCT. RoB 2 risk of bias judgement: Some concerns. Non-blinded. No pre-specified analysis plan stated.
Ingram et al. (2022). *Health Expectations*. Centre for Academic Child Health, University of Bristol, United Kingdom. Sample: 11 mothers and 10 fathers of 11 infants treated for HIE with TH who received the CoolCuddle intervention during TH in two NICUs, and four doctors and six nurses from the same NICUs.	Aims: Explore and compare the views and experiences of staff and parents in relation to the CoolCuddle intervention. Design: Qualitative study using one-to-one semi-structured interviews and thematic analysis methods.	Level VI – Qualitative study. No stated philosophical perspective or research methodology. No statement locating or regarding the influence of the researcher.
Jain et al. (2021). *Iranian Journal of Neonatology*. Department of Pediatrics, Mahatma Gandhi Memorial Medical College, India. Sample: 176 infants born ≥ 36 weeks with moderate to severe HIE, treated with either TH or standard care in a single NICU.	Aim: Assess the effectiveness and feasibility of using a TH protocol in a limited-resource setting. Design: Non-randomised prospective cohort study. Intervention: 72 hr of TH at 33.5 ± 0.5°C initiated within 6 hr of birth via a phase-changing material device, followed by rewarming over 10–12 hours at 0.5°C/hr. Control condition: Standard supportive care with temperature maintained at 36.5°C. Outcome: Successful breastfeeding at discharge.	Level III – Controlled trial without randomisation. Non-randomised study. Infants with delayed admission/not eligible for 6-hr cooling window assigned to standard care, potentially introducing confounding differences between groups. No statement of strategies to deal with confounding variables. Outcome not specifically defined.
Jannatdoust et al. (2014). *Iranian Journal of Neonatology*. Pediatric Health Research Center, Tabriz University of Medical Sciences, Iran. Sample: 16 infants born ≥ 35 weeks gestational age treated for HIE with either head or whole-body TH in two hospitals.	Aim: Compare outcomes for head and whole-body cooling methods of TH. Design: Prospective, non-randomised cohort analysis. Intervention 1: 72 hr of selective head TH (33–35°C) initiated within 6 hr of birth, then 6 hr of re-warming. Intervention 2: 72 hr of whole body TH (33–35°C) initiated within 6 hr of birth, then 6 hr of re-warming. Outcome: Onset of breastfeeding.	Level IV – Cohort study. Intervention 1 and Intervention 2 participants treated at different hospitals, potentially introducing group differences. No analysis of differences between groups. Outcome not specifically defined.
Malviya et al. (2025). *Children*. Department of Neonatology, Khoula Hospital, Oman. Sample: 187 infants born ≥ 35 weeks with moderate to severe HIE, treated with TH in a single NICU.	Aim: Investigate the short-term outcomes of infants treated with TH. Design: Single-centre retrospective observational cohort study. Independent variables: HIE severity (modified Sarnat staging) and MRI score (Washington University of Neonatal Neurology and Physiology Research laboratory MRI scoring system). Outcome: All breastfeeding at discharge.	Level IV – Cohort study. Used routinely collected clinical data with inherent risk of poor data quality. Outcome not specifically defined.
Nassef et al. (2023). *Nursing Open*. Division of Paediatrics, Karolinska Institutet, Sweden. Sample: 13 mothers and eight fathers of 15 infants born ≥ 35 weeks and treated with TH in two NICUs.	Aim: Describe parent experiences of their infant’s TH treatment, 10–13 years following the event. Design: Qualitative descriptive study using focus groups and framework analysis methods.	Level VI – Qualitative study. No stated philosophical perspective.
Savitha & Prakash (2016). *International Journal of Contemporary Pediatrics*. Department of Pediatrics, Mysore Medical College and Research Institute, India. Sample: 120 term-born infants with HIE of any grade admitted to a single NICU.	Aim: Determine the role of magnesium sulphate therapy for recovery and neurology outcomes following perinatal asphyxia. Design: Randomised cohort comparison intervention study. Intervention: Routine birth asphyxia protocol plus intravenous infusion of magnesium sulphate within 6 hr of birth at 250 mg/kg/dose over 1 hr and an additional two doses given after 24 hr and 48 hr. Control condition: Routine birth asphyxia protocol (no further details provided). Outcomes: Duration (in days) for initiation of direct breastfeeding; normal suck and on direct breastfeeding at discharge.	Level II – RCT RoB 2 risk of bias judgement: High risk. Not stated if caregivers and clinicians blinded. Outcomes not defined. Not stated how decisions about breastfeeding initiation were reached or how suck was assessed. No pre-specified analysis plan stated.
Sreenivasa et al. (2017). *Sri Lanka Journal of Child Health*. Basaveshwara Medical College Hospital and Research Centre, India. Sample: 100 term-born infants with HIE of any grade admitted to a single NICU.	Aim: Assess the efficacy of magnesium sulphate for treatment of birth asphyxia. Design: Cohort comparison intervention study. Intervention: Routine birth asphyxia protocol plus intravenous infusion of magnesium sulphate within 6 hr of birth at 250 mg/kg/dose over 1 hr and an additional two doses given after 24 hr and 48 hr. Control condition: Routine birth asphyxia protocol (no further details provided). Outcome: Duration (in days) for initiation of direct breastfeeding.	Level III – Controlled trial, (randomisation unclear). RoB 2 risk of bias judgement: High risk. Group allocation method and use of blinding not stated. Outcome not defined. Not stated how decisions about breastfeeding initiation were reached. No pre-specified analysis plan stated.
Thyagarajan et al. (2015). *Acta Paediatrica*. Princess Anne Hospital, United Kingdom. Sample: 85 infants born ≥ 36 weeks gestational age with moderate to severe HIE and treated with TH in three neonatal units: two in Sweden and one in the UK.	Aim: Explore whether enteral feeding during therapeutic hypothermia is beneficial for feeding outcomes. Design: Retrospective cohort comparison study Intervention: EF during TH hypothermia (Swedish cohort). 91% had EF alongside PN during TH, starting with three hourly boluses of 1–2 ml/kg and increased or stopped depending on tolerance. Control condition: No EF during TH (UK cohort). Predominantly fed using PN without EF during TH, 33% also offered small amounts of BM. Outcomes: Breastfeeding at discharge (receiving full breastfeeds or both breast and bottle feeding); exclusively bottle feeding at discharge.	Level IV – Cohort study. Groups recruited from different population. Used routinely collected clinical data with inherent risk of poor data quality. Exposure to intervention not measured in valid and reliable way. Confounding factors identified. No statement of strategies to deal with confounding factors. Outcomes not specifically defined regarding inclusion of expressed BM and formula.
Varghese et al. (2024). *International Journal of Medicine and Public Health*. James Cook University Hospital, United Kingdom. Sample: 95 infants born ≥ 37 weeks gestational age with any grade of HIE in a single NICU in India.	Aim: Study outcomes and complications in the first 10 days of life of infants treated for perinatal asphyxia using magnesium sulphate. Design: Prospective randomised controlled trial. Intervention: Routine birth asphyxia protocol plus magnesium sulphate infusion at 250 mg/kg/dose over 1 hr within 1 hr of birth, plus two additional doses at 125 mg/kg/dose over 1 hr at 24-hr intervals. Control condition: Routine birth asphyxia protocol (no further details provided). Outcome: Hours to initiation of direct breastfeeding.	Level II – RCT RoB 2 risk of bias judgement: High risk. Group allocation sequence not random. Baseline differences between groups. Use of blinding not stated. Outcome not defined. Not stated how decisions about breastfeeding initiation were reached. No pre-specified analysis plan stated.

*Note.* BF = breastfeeding; BM = breastmilk; EF = enteral feeding; HIE = hypoxic ischemic encephalopathy; NICU = neonatal intensive care unit; PN = parenteral nutrition; RoB 2 = Revised Cochrane Risk-of-Bias Tool for Randomized Trials; RCT = randomised controlled trial.

a Levels of evidence were determined using the Melnyk and Fineout-Overholt model. For details, see: Stillwell, S. B., Fineout-Overholt, E., Melnyk, B. M. & Williamson, K. M. (2010). Evidence-based practice, step by step: searching for the evidence. *American Journal of Nursing*, *110*(41).

Consistent with convergent integrated methods (Stern et al., 2020), relevant quantitative data were “qualitised” by transforming them into mutually compatible textual narrative forms in preparation for integration with qualitative data. Relevant extracts from qualitative data and qualitised narratives of quantitative data were labelled with the factor hypothesised to influence breastfeeding and lactation outcomes. Data were then pooled together and tabulated under headings for each identified factor. Final categories were formed by either retaining the originally identified influencing factor heading or, where factors were closely related, by combining headings into a broader category. For example, combining interventions of the same type or mechanism.

### Data Analysis

Of the 17 papers identified, 12 used quantitative and five used qualitative methods. The included studies originated from a range of geographical and healthcare contexts, including Europe (UK, Sweden), North America (Canada, USA), the Middle East (Iran, Oman), South Asia (India), East Asia (China), and Turkey.

All papers were quality assessed using the relevant JBI critical appraisal tool or Revised Cochrane Risk-of-Bias Tool for Randomized Trials (RoB 2; Sterne et al., 2019).

### Quality Assessment

Six papers were cohort studies (Alburaki et al., 2022; Deveci et al., 2025; Gale et al., 2021; Jain et al., 2021; Malviya et al., 2025; Thyagarajan et al., 2015). Evaluation using the JBI Checklist for Cohort Studies (JBI, 2023) found the validity of outcome measure in all six was unclear, and potentially confounding factors were identified, particularly for the studies by Deveci et al. (2025) and Thyagarajan et al. (2015). Gale et al. (2021) mitigated potential confounders using statistical analysis techniques.

A non-randomised trial (Jannatdoust et al., 2014) evaluated using JBI Checklist for Quasi-Experimental Studies (Barker et al., 2024) identified confounding factors, the validity of the outcome measure was not clear, and it was not clear that all enrolled participants had been included in the analysis.

Five randomised controlled trials (RCTs) were assessed using the Revised Cochrane Risk-of-Bias Tool for Randomized Trials (RoB 2). Two scored as “some concern” regarding lack of intention-to-treat analysis and lack of a previously published analysis protocol (DuPont et al., 2021; Hu et al., 2022). Papers by Savitha & Prakash (2016) and Varghese et al. (2024) scored as “high risk of bias” due to lack of blinding, lack of intention-to-treat analysis and missing outcome data, risk of bias in outcome measurement, and risk of bias in selection of reported results. Varghese et al. (2024) also used quasi-randomisation rather than true randomisation. The final paper (Sreenivasa et al., 2017) did not make clear if it was a randomised study or not; however, use of the RoB 2 allowed direct comparison with other included papers. The paper scored as “high risk of bias” due to lack of randomisation, lack of blinding, lack of intention-to-treat analysis and missing outcome data, risk of bias in outcome measurement, and risk of bias in selection of reported results.

Five qualitative papers were evaluated using the JBI Checklist for Qualitative Studies (Lockwood et al., 2015). Two stated the underlying methodology and both used methods that aligned with this (Beasant et al., 2024; Nassef et al., 2023). Only one paper (Nassef et al., 2023) described the researchers and their use of reflexivity. None identified the philosophical perspective of the researchers. All studies represented the voices of participants and drew conclusions that flowed from the analysed data.

## Results

### Factors Influencing Breastfeeding and Lactation Outcomes Following Neonatal HIE

Five categories of influencing factors were identified in this review: infant medical factors, neuroprotective interventions, feeding during therapeutic hypothermia, information and support for expressing milk, and parent-infant closeness in the neonatal phase.

### Infant Medical Factors

One study analysed short-term breastfeeding outcomes in relation to HIE severity grading and MRI scores. Malviya et al. (2025) reported data for 187 infants who received therapeutic hypothermia for moderate to severe HIE. Feeding status at discharge was reported for 164 of 165 surviving infants. A significantly higher proportion of infants with moderate HIE were exclusively breastfed at discharge compared to those with severe HIE (*n* = 66, 62% vs. *n* = 19, 25%, *p* = .001). The authors also reported that brain MRI scores were a significant predictor of exclusive breastfeeding at discharge (area under the curve: 0.73; *p* < .001).

### Neuroprotective Interventions

Six studies examined the outcomes of neuroprotective interventions on breastfeeding and lactation outcomes following neonatal HIE.

Two studies assessed the effect of therapeutic hypothermia on breastfeeding and lactation. Jain et al. (2021) found that more infants were successfully breastfeeding at discharge compared to non-treated infants (*n* = 76, 92.6% vs. *n* = 47, 70.1%, chi-square test *p* = .003, risk ratio = 1.32, 95% CI [1.11, 1.56]). A non-randomised trial comparing two methods of providing therapeutic hypothermia on onset of breastfeeding during neonatal admission found no significant difference between infants who received selective head cooling versus whole body cooling (5 ± 2 days vs. 8 ± 5 days, Mann-Whitney U test *p* = .50; Jannatdoust et al., 2014).

One RCT examined the effect of darbepoetin compared to placebo on proportions receiving “breast milk” at discharge (DuPont et al., 2021) but found no significant difference between groups (*n* = 7, 77.8% vs. *n* = 7, 58.3%, Fisher’s exact test *p* > 0.05, no further statistics reported). The authors did not state if this outcome included any human milk or only parent’s milk.

Three clinical trials (two randomised, one not specified) examined the effect of magnesium sulphate versus routine care on time to initiation of direct breastfeeding (Savitha & Prakash, 2016; Sreenivasa et al., 2017; Varghese et al., 2024). All studies reported significantly earlier breastfeeding initiation for infants treated with magnesium sulphate compared to those receiving routine care (4.6 ± 1.36 vs. 6.0 ± 1.51 days, *p* < .001; 4.90 ± 1.56 vs. 6.30 ± 1.67 days, *p* < .001; 136 hours vs. 165 hours (*SD* not reported), *p* < .001). However, risk of bias was assessed as “high” for all papers.

### Feeding During Therapeutic Hypothermia

Five studies examined the effect of parenteral and enteral feeding during therapeutic hypothermia on breastfeeding and lactation outcomes following neonatal HIE.

A retrospective cohort study by Gale et al. (2021) found parenteral feeding during therapeutic hypothermia significantly reduced time to first feed of parent’s own milk compared to non-parenteral methods (two-sample *t* test after propensity score matching, median 4.6 vs. 4.9 days, difference = −0.2 days, 95% CI [–0.4, –0.1]; *p* = .01). However, this did not influence time to onset of breastfeeding (median 8.6 days vs. 8.4 days, difference 0.2 days, 95% CI = 0.5 to 0.8 days; *p* = .56) or breastfeeding at discharge (*n* = 575, 46.4% vs. *n* = 582, 47.0%, rate difference 0.6%, 95% CI = –3.8% to 2.6%; *p* = .71).

The same study found enterally fed infants received their first feed of parent’s own milk significantly earlier than those not enterally fed (alternative nutrition not stated; two-sample *t* test after propensity score matching, median 3.3 vs. 5.4 days, difference = –2.1 days, 95% CI = 2.2 to 2.0 days; *p* < .001). Enterally fed infants were also faster to commence direct suckling at the breast (two-sample *t* test after propensity score matching, median 7.3 days vs. 8.7 days, difference = –1.4 days, 95% CI = –1.9 to –0.9 days; *p* < .001; Gale et al., 2021).

Two studies examined the effect of enteral feeding during therapeutic hypothermia on type of milk given at enteral feeding initiation. A prospective cohort study with historical controls (Alburaki et al., 2022) found one infant (1.4%) who was enterally fed during therapeutic hypothermia received some formula, alongside human milk (donor or expressed), with their first enteral feed, compared to 10 (13%) of those who received parenteral nutrition but no enteral feeds during hypothermia (chi-square test *p* < .001; no further statistics reported). In contrast, Hu et al. (2022) reported high numbers exclusively receiving formula at first enteral feed among both groups in their RCT, regardless of whether participants received enteral feeds during therapeutic hypothermia or parenteral nutrition only (*n* = 39, 92.86% vs. *n* = 36, 94.74%, Fisher’ exact test *p* = 1.000).

One study examining the effect of commencing enteral feeding during therapeutic hypothermia on formula use reported that infants who received minimal enteral nutrition during therapeutic hypothermia had lower rates of formula use than those who received no enteral nutrition (Deveci et al., 2025). However, the difference between groups was not significant (*n* = 21, 52.5% vs. *n* = 32, 62.7%, *p* = .442).

Four studies investigated the effect of enteral feeding during therapeutic hypothermia on breastfeeding outcomes at discharge from the neonatal unit. Alburaki et al. (2022) specifically looked at exclusive human milk provision (either directly from the breast or via expressed or donor milk) and reported a significantly higher proportion of exclusive human milk feeding among infants who had been enterally fed during therapeutic hypothermia compared to those who were parenterally fed (*n* = 29, 41% vs. *n* = 10, 13%, chi-square test *p* < .001; no further statistics reported). Hu et al. (2022) looked at full or partial parent’s own milk feeds versus exclusive formula feeding and found little difference between enterally and parenterally fed groups at discharge (*n* = 29, 69.05% vs. *n* = 21, 55.26%, Fisher’ exact test *p* = .203; no further statistics reported). Gale et al. (2021) and Thyagarajan et al. (2015) both examined the effect of enteral feeding during therapeutic hypothermia on full or partial direct breastfeeding at discharge. Gale et al. (2021) reported that full or partial direct breastfeeding at discharge was significantly more common among infants who had received enteral feeds during therapeutic hypothermia compared to those who had not (*n* = 883. 54.6% vs. *n* = 752, 46.5%, rate difference 8.0%, 95% CI = 5.1% to 10.8%; *p* < .001). Thyagarajan et al. (2015) found a trend toward higher proportions who were fully or partially fed by direct breastfeeding at discharge between their Swedish cohort, the majority of whom were enterally fed during therapeutic hypothermia, and their UK cohort, most of whom received only parenteral nutrition, but this did not reach statistical significance (chi-square test 85% vs. 67%, *p* = 0.08; no frequencies or further statistics reported). Alburaki et al. (2022) followed their cohort after discharge to 4 months old, reporting the benefits of enteral feeding during therapeutic hypothermia to breastfeeding rates seen at neonatal discharge were not maintained, and by follow-up there was no significant difference between enterally and parenterally fed groups (*n* = 12, 17% vs. *n* = 5, 7%, chi-square test *p* = .73).

### Information and Support for Expressing Milk

Support and information about expressing milk for their infant has been identified as important for HIE-affected families. A qualitative study by Bäcke et al. (2021) examined 11 parents’ experiences of therapeutic hypothermia. They reported that parents conveyed a great need for clear information during this time, including about expressing milk. Similarly, Nassef et al. (2023) studied 21 parents’ experiences of therapeutic hypothermia. In this study, parents reported receiving insufficient information about expressing milk for their infant and also described not being monitored or asked about their progress.

### Parent–Infant Closeness in the Neonatal Phase

Craig et al. (2020) interviewed 30 parents and grandparents about their experiences of therapeutic hypothermia. Parents described losing the opportunity for skin-to-skin contact and breastfeeding as a result of their infants’ neonatal admission and therapeutic hypothermia. One parent described how separation from her infant, compounded by the stress of the neonatal admission, resulted in unwanted formula supplementation.

To reduce parent–infant separation, the CoolCuddle device was developed, allowing parents to hold their infant on a pillow during therapeutic hypothermia. Two studies have reported parent and staff perceptions of this device. Ingram et al. (2022) interviewed 21 parents and 10 staff members about their experiences of CoolCuddle. They reported parents felt that the opportunity to be physically close to their infant helped establish their milk supply and mitigated barriers to breastfeeding. Staff also felt the physical closeness and bonding enabled by the device had a positive effect on milk supply. Another qualitative study (Beasant et al., 2024) examined the views of 14 neonatal nurses regarding supporting parents to use the CoolCuddle. Like Ingram et al. (2022), participants felt the opportunities for parent–infant closeness and bonding provided by the CoolCuddle had a positive effect on breastfeeding and lactation.

## Discussion

This mixed methods systematic review aimed to answer the question: What factors influence breastfeeding and lactation outcomes after neonatal HIE? To our knowledge, this is the first review examining this topic. Seventeen papers were identified, incorporating both quantitative and qualitative data. Five categories of factors influencing post-HIE breastfeeding and lactation outcomes were identified: infant medical factors, neuroprotective interventions, feeding during therapeutic hypothermia, support for expressing milk, and parent–infant closeness in the neonatal phase. With one exception, quantitative outcomes were limited to breastfeeding metrics in the neonatal phase.

The identified qualitative studies provided insights into some of the factors that parents and healthcare professionals felt may influence post-HIE breastfeeding and lactation outcomes. Although these studies emphasised the importance of breastfeeding and lactation in relation to their topic, the topics were broad in nature and did not explore feeding, breastfeeding, or lactation in depth. It is likely that targeted qualitative work would provide further valuable insights.

Regarding neuroprotective interventions, the commonly used therapeutic hypothermia treatment has only been evaluated for breastfeeding and lactation outcomes against a control group in one study, and only one small study has examined darbepoetin. The three studies investigating magnesium sulphate all reported a positive effect on breastfeeding and lactation but were identified as having high risk of bias. Due to the low numbers of studies, low participant numbers and methodological issues, it is not possible to draw conclusions regarding the effect of neuroprotective treatments on breastfeeding and lactation outcomes following neonatal HIE.

The studies of feeding during therapeutic hypothermia were limited to the effect of parenteral and enteral nutrition methods. Although there is evidence to support these interventions, particularly for enteral feeding, findings remain mixed and there were methodological issues in these studies. However, the largest of these studies (Gale et al., 2021) had comparatively few areas of potential bias and overall provided support for the use of enteral feeding during therapeutic hypothermia.

None of the identified interventions specifically targeted skills for breastfeeding or supporting mothers to express milk, suggesting a lack of focus on improving breastfeeding and lactation for HIE-affected families.

As well as low numbers of studies, small samples, and methodological quality issues, the outcomes used in the identified quantitative studies were not identical, making comparisons challenging. The most widely studied intervention-outcome combination (the effect of enteral feeding on breastfeeding rates at discharge) was reported in four papers, with three different definitions for the outcome used: exclusive human milk feeds, full or partial human milk feeds, and full or partial direct breastfeeding.

Additionally, institutional policies and cultural differences within neonatal units and in the wider social context, are likely to influence feeding practices and outcomes following HIE. The 17 included studies were conducted across a range of international healthcare settings. Models of neonatal care, access to breastfeeding and lactation support, and cultural norms surrounding infant feeding are likely to differ substantially between the study settings. Therefore, the relevance and applicability of findings from the included studies will also vary between countries and healthcare systems and contextual factors should be considered when interpreting and applying findings to settings outside those in which the original studies were conducted.

This review demonstrates a lack of understanding regarding factors that influence breastfeeding and lactation following neonatal HIE. No studies identified in this review used formal feeding measures, therefore it is not currently possible to quantify the specific aspects of breastfeeding and lactation that are problematic following neonatal HIE. Studies of parent experience did not explore breastfeeding or lactation in depth but included these areas as part of the wider experience of having an infant receiving therapeutic hypothermia. Additionally, only two factors relating to the infant’s medical history (HIE severity and brain MRI findings) were examined, and only in a single paper, and none of the identified studies examined social and environmental factors or availability of support services. Given what is already known about factors affecting breastfeeding and lactation in the wider literature, it is necessary to study risk factors related to individual characteristics, medical history, and the social circumstances in which families affected by neonatal HIE are living and operating. It is only by understanding these factors that we can better identify the families likely to be at higher risk of difficulties with breastfeeding and lactation and better select and develop the interventions that will target those particular challenges.

## Limitations

In this review, we searched a large number of databases without language restrictions, including screening many review papers, subsequent citations of included papers, and reference lists to minimise the risk of missing relevant papers. However, this review did not incorporate literature not commercially published, which may have added further information about factors influencing breastfeeding and lactation following neonatal HIE. All identified papers had methodological issues, with some rated as being poor quality and having high risk of bias. Additionally, although papers in any language were eligible for inclusion, the eligible papers identified were all published in the English language. It is possible that relevant papers with titles in languages other than English were not identified in database searches.

## Conclusion

This systematic review identified a small number of factors that may influence breastfeeding and lactation outcomes following neonatal HIE. However, there are significant evidence gaps in the published literature. Studies on a wider range of individual, environmental and social factors, including risk factors for breastfeeding and lactation challenges, and studies of breastfeeding and lactation-specific interventions represent significant areas in need of further research. Qualitative and mixed methods studies specifically focused on this topic area are also needed to ensure a comprehensive understanding of the challenges and the support and interventions required for breastfeeding and lactation following neonatal HIE.

## Supplemental Material

sj-docx-1-jhl-10.1177_08903344261426707 – Supplemental material for Factors Influencing Breastfeeding Outcomes Following Neonatal Hypoxic Ischaemic Encephalopathy: A Mixed Methods Systematic Review Supplemental material, sj-docx-1-jhl-10.1177_08903344261426707 for Factors Influencing Breastfeeding Outcomes Following Neonatal Hypoxic Ischaemic Encephalopathy: A Mixed Methods Systematic Review by Sarah Edney, Anna Basu, Anne Breaks, Nadia Leake, Judith Rankin, Farag Shuweihdi, Mari Viviers, Kirstin Webster and Lindsay Pennington in Journal of Human Lactation

sj-docx-2-jhl-10.1177_08903344261426707 – Supplemental material for Factors Influencing Breastfeeding Outcomes Following Neonatal Hypoxic Ischaemic Encephalopathy: A Mixed Methods Systematic Review Supplemental material, sj-docx-2-jhl-10.1177_08903344261426707 for Factors Influencing Breastfeeding Outcomes Following Neonatal Hypoxic Ischaemic Encephalopathy: A Mixed Methods Systematic Review by Sarah Edney, Anna Basu, Anne Breaks, Nadia Leake, Judith Rankin, Farag Shuweihdi, Mari Viviers, Kirstin Webster and Lindsay Pennington in Journal of Human Lactation
