# Supplementary material for: Factors Influencing Breastfeeding Outcomes Following Neonatal Hypoxic Ischaemic Encephalopathy: A Mixed Methods Systematic Review
Source: J Hum Lact. 2026 Apr 3;42(2):302–14. doi: 10.1177/08903344261426707 (PMC13263470; doi:10.1177/08903344261426707)
Supplement: sj-docx-1-jhl-10.1177_08903344261426707 – Supplemental material for Factors Influencing Breastfeeding Outcomes Following Neonatal Hypoxic Ischaemic Encephalopathy: A Mixed Methods Systematic Review [file sj-docx-1-jhl-10.1177_08903344261426707.docx]

**Supplementary table: Excluded papers**

| **Authors** | **Year** | **Title** | **Journal** | **Volume** | **Issue** | **Pages** | **Reason for exclusion** |
| --- | --- | --- | --- | --- | --- | --- | --- |
| Abdel-Aziz, S. M., Rahman, M., Shoreit, A. H., El Din, M. E., Hamed, E. A. and Gad, E. F. | 2021 | Outcome of Infants with Hypoxic-Ischemic Encephalopathy Treated by Whole Body Cooling and Magnesium Sulfate | Journal of Child Science | 11 | 1 | E280-E286 | No analysis of breastfeeding or lactation outcomes |
| Abdulqawi, K. and Al-Zohairy, Y. Z. and Karam, K. | 2011 | Early predictors of neurodevelopmental adverse outcome in term infants with postasphyxial hypoxic ischemic encephalopathy | International Journal of Collaborative Research on Internal Medicine and Public Health | 3 | 11 | 822-837 | No feeding outcomes reported |
| Abedin, A. F. Z. and McCallion, N. and Thomas, M. and Barry, D. and Molloy, E. J. | 2010 | Normal troponin is associated with severe encephalopathy and poor neurodevelopmental outcome in infants following perinatal asphyxia | Irish Journal of Medical Science | 179 |  | S177-S177 | Conference abstract |
| Adams, L. and Badran, B. and Dancy, M. and Huffman, S. and O'Leary, G. and George, M. and Jenkins, D. | 2021 | Increasing the number of daily stimulation sessions administered during taVNS-paired bottle feeding speeds response time in newborns with feeding difficulty | Brain Stimulation | 14 | 6 | 1703 | Conference abstract |
| Airede, K. I. | 1991 | Neonatal seizures and a 2-year neurological outcome | Journal of Tropical Pediatrics | 37 | 6 | 313-7 | No analysis of factors influencing HIE feeding outcomes |
| Al Amrani, Fatema and Kwan, Saskia and Gilbert, Guillaume and Saint-Martin, Christine and Shevell, Michael and Wintermark, Pia | 2017 | Early Imaging and Adverse Neurodevelopmental Outcome in Asphyxiated Newborns Treated With Hypothermia | Pediatric Neurology | 73 |  | 20-27 | No feeding outcomes reported |
| Al Amrani, Fatema and Marcovitz, Jaclyn and Sanon, Priscille-Nice and Khairy, May and Saint-Martin, Christine and Shevell, Michael and Wintermark, Pia | 2018 | Prediction of outcome in asphyxiated newborns treated with hypothermia: Is a MRI scoring system described before the cooling era still useful? | European journal of paediatric neurology : EJPN : official journal of the European Paediatric Neurology Society | 22 | 3 | 387-395 | No analysis of breastfeeding or lactation outcomes |
| Al Naqeeb, N. and Edwards, A. D. and Cowan, F. M. and Azzopardi, D. | 1999 | Assessment of neonatal encephalopathy by amplitude-integrated electroencephalography | Pediatrics | 103 | 6 | 1263-1271 | No feeding outcomes reported |
| Alderliesten, T. and de Vries, L. S. and Khalil, Y. and van Haastert, I. C. and Benders, Mjnl and Koopman-Esseboom, C. and Groenendaal, F. | 2015 | Therapeutic Hypothermia Modifies Perinatal Asphyxia-Induced Changes of the Corpus Callosum and Outcome in Neonates | Plos One | 10 | 4 |  | No feeding outcomes reported |
| Alderliesten, Thomas and de Vries, Linda S. and Benders, Manon J. N. L. and Koopman, Corine and Groenendaal, Floris | 2011 | MR imaging and outcome of term neonates with perinatal asphyxia: value of diffusion-weighted MR imaging and 1H MR spectroscopy | Radiology | 261 | 1 | 235-42 | No feeding outcomes reported |
| Alderliesten, Thomas and de Vries, Linda S. and Staats, Liza and van Haastert, Ingrid C. and Weeke, Lauren and Benders, Manon J. N. L. and Koopman-Esseboom, Corine and Groenendaal, Floris | 2017 | MRI and spectroscopy in (near) term neonates with perinatal asphyxia and therapeutic hypothermia | Archives of disease in childhood. Fetal and neonatal edition | 102 | 2 | F147-F152 | No feeding outcomes reported |
| Aljuhani, T. and Coker-Bolt, P. and Katikaneni, L. and Ramakrishnan, V. and Brennan, A. and George, M. S. and Badran, B. W. and Jenkins, D. | 2023 | Use of non-invasive transcutaneous auricular vagus nerve stimulation: neurodevelopmental and sensory follow-up | Frontiers in Human Neuroscience | 17 |  | 1297325 | Mixed cohort |
| Aly, H. and Abd-Rabboh, L. and El-Dib, M. and Nawwar, F. and Hassan, H. and Aaref, M. and Abdelrahman, S. and Elsayed, A. | 2009 | Ascorbic acid combined with ibuprofen in hypoxic ischemic encephalopathy: a randomized controlled trial | Journal of perinatology : official journal of the California Perinatal Association | 29 | 6 | 438-43 | No analysis of breastfeeding or lactation outcomes |
| Amess, P. N. and Penrice, J. and Wylezinska, M. and Lorek, A. and Townsend, J. and Wyatt, J. S. and Amiel-Tison, C. and Cady, E. B. and Stewart, A. | 1999 | Early brain proton magnetic resonance spectroscopy and neonatal neurology related to neurodevelopmental outcome at 1 year in term infants after presumed hypoxic-ischaemic brain injury | Developmental Medicine and Child Neurology | 41 | 7 | 436-445 | No feeding outcomes reported |
| Andorka, C. and Barta, H. and Sesztak, T. and Nyilas, N. and Kovacs, K. and Dunai, L. and Rudas, G. and Jermendy, A. and Szabo, M. and Szakmar, E. | 2025 | The predictive value of MRI scores for neurodevelopmental outcome in infants with neonatal encephalopathy | Pediatric Research | 97 | 1 | 253-260 | No feeding outcomes reported |
| Annink, K. V. and de Vries, L. S. and Groenendaal, F. and Vijlbrief, D. C. and Weeke, L. C. and Roehr, C. C. and Lequin, M. and Reiss, I. and Govaert, P. and Benders, Mjnl and Dudink, J. | 2020 | The development and validation of a cerebral ultrasound scoring system for infants with hypoxic-ischaemic encephalopathy | Pediatric Research | 87 |  | 59-66 | No feeding outcomes reported |
| Aoki, H. and Shibasaki, J. and Tsuda, K. and Yamamoto, K. and Takeuchi, A. and Sugiyama, Y. and Isayama, T. and Mukai, T. and Ioroi, T. and Yutaka, N. and Takahashi, A. and Tokuhisa, T. and Nabetani, M. and Iwata, O. and Japan Collaboration, Team |  | Predictive value of the Thompson score for short-term adverse outcomes in neonatal encephalopathy | Pediatric Research |  |  |  | Feeding as part of a composite outcome |
| Aoki, Yoshinori and Kono, Tatsuo and Enokizono, Mikako and Okazaki, Kaoru | 2021 | Short-term outcomes in infants with mild neonatal encephalopathy: a retrospective, observational study | BMC pediatrics | 21 | 1 | 224 | No feeding outcomes reported |
| Arango, Jorge I. and Allred, Kimberlee and Adelson, P. David and Soni, Parita and Stradleigh, Ryan and Wahnoun, Remy and Carballo, Cristina | 2014 | Hypothermia in hypoxic ischemic encephalopathy: a 5-year experience at Phoenix Children's Hospital Neuro NICU | Advances in pediatrics | 61 | 1 | 215-23 | No analysis of factors influencing HIE feeding outcomes |
| Artzi, M. and Ben Sira, L. and Bassan, H. and Gross-Tsur, V. and Berger, I. and Marom, R. and Leitner, Y. and Bental, Y. and Shiff, Y. and Geva, R. and Weinstein, M. and Ben Bashat, D. | 2011 | Brain Diffusivity in Infants With Hypoxic-Ischemic Encephalopathy Following Whole Body Hypothermia: Preliminary Results | Journal of Child Neurology | 26 | 10 | 1230-1236 | No feeding outcomes reported |
| Atici, A. and Celik, Y. and Gulasi, S. and Turhan, A. H. and Okuyaz, C. and Sungur, M. A. | 2015 | Comparison of selective head cooling therapy and whole body cooling therapy in newborns with hypoxic ischemic encephalopathy: Short term results | Turk Pediatri Arsivi | 50 | 1 | 27-36 | No analysis of breastfeeding or lactation outcomes |
| Auriti, C. and Prencipe, G. and Inglese, R. and Moriondo, M. and Nieddu, F. and Mondi, V. and Longo, D. and Bucci, S. and Del Pinto, T. and Timelli, L. and Di Ciommo, V. M. | 2020 | Mannose Binding Lectin, S100 B Protein, and Brain Injuries in Neonates With Perinatal Asphyxia | Frontiers in Pediatrics | 8 |  |  | No feeding outcomes reported |
| Avann, C. and Bowen, J. S. and Mahadevan, S. | 2019 | Hypoxic ischaemic encephalopathy (HIE): An audit of assessment, management and follow up in a level 2 neonatal unit | European Journal of Pediatrics | 178 | 11 | 1726 | Conference abstract |
| Aziz, K. B. and Kuiper, J. and Kilborn, A. and Kambli, H. and Jayakumar, S. and Gerner, G. J. and Tekes, A. and Parkinson, C. and Graham, E. M. and Stafstrom, C. E. and Campbell, C. and Demos, C. and Stengelin, M. and Sigal, G. and Wohlstadter, J. and Everett, A. D. and Northington, F. J. and Chavez-Valdez, R. | 2025 | Seizures May Worsen Outcomes of Neonatal Hypoxic-Ischemic Encephalopathy: A Longitudinal Serum Biomarkers Study | Pediatric Neurology | 166 |  | 55-64 | No analysis of breastfeeding or lactation outcomes |
| Azzopardi, D. and Guarino, I. and Brayshaw, C. and Cowan, F. and Price-Williams, D. and Edwards, A. D. and Acolet, D. | 1999 | Prediction of neurological outcome after birth asphyxia from early continuous two-channel electroencephalography | Early Human Development | 55 | 2 | 113-123 | No feeding outcomes reported |
| Azzopardi, D. and Strohm, B. and Edwards, A. D. and Halliday, H. and Juszczak, E. and Levene, M. and Thoresen, M. and Whitelaw, A. and Brocklehurst, P. and Azzopardi, D. and Strohm, B. and Edwards, A. D. and Halliday, H. and Juszczak, E. and Levene, M. and Thoresen, M. and Whitelaw, A. and Brocklehurst, P. | 2009 | Treatment of asphyxiated newborns with moderate hypothermia in routine clinical practice: how cooling is managed in the UK outside a clinical trial | Archives of Disease in Childhood -- Fetal & Neonatal Edition | 94 | 4 | F260-4 | No analysis of factors influencing HIE feeding outcomes |
| Azzopardi, D. V. and Strohm, B. and Edwards, A. D. and Dyet, L. and Halliday, H. L. and Juszczak, E. and Kapellou, O. and Levene, M. and Marlow, N. and Porter, E. and Thoresen, M. and Whitelaw, A. and Brocklehurst, P. | 2009 | Moderate hypothermia to treat perinatal asphyxial encephalopathy | New England Journal of Medicine | 361 | 14 | 1349-1358 | No analysis of breastfeeding or lactation outcomes |
| Bachu, Sreekruthi and Hariharan, Gopakumar | 2021 | Does fluid balance affect neurodevelopmental outcomes in hypoxic-ischaemic encephalopathy? | Acta paediatrica (Oslo, Norway : 1992) | 110 | 11 | 3140-3141 | Commentary paper |
| Badr, L. K. | 2009 | Statistical Versus Clinical Significance for Infants With Brain Injury Reanalysis of Outcome Data From a Randomized Controlled Study | Clinical Nursing Research | 18 | 2 | 136-152 | No feeding outcomes reported |
| Badran, Bashar W. and Jenkins, Dorothea D. and Cook, Daniel and Thompson, Sean and Dancy, Morgan and DeVries, William H. and Mappin, Georgia and Summers, Philipp and Bikson, Marom and George, Mark S. | 2020 | Transcutaneous Auricular Vagus Nerve Stimulation-Paired Rehabilitation for Oromotor Feeding Problems in Newborns: An Open-Label Pilot Study | Frontiers in human neuroscience | 14 |  | 77 | No analysis of factors influencing HIE feeding outcomes |
| Bahr, Timothy M. and Ohls, Robin K. and Baserga, Mariana C. and Lawrence, Shelley M. and Winter, Sarah L. and Christensen, Robert D. | 2022 | Implications of an Elevated Nucleated Red Blood Cell Count in Neonates with Moderate to Severe Hypoxic-Ischemic Encephalopathy | The Journal of pediatrics | 246 |  | 12-18.e2 | No analysis of breastfeeding or lactation outcomes |
| Balestri, M. and Luisa Lispi, M. and Pro, S. and Longo, D. and Vigevano, F. and Cilio, M. R. | 2015 | The prognostic value of early eeg background during hypothermia on mri and neurodevelopmental outcome in neonates with hypoxic-ischemic encephalopathy | Epilepsy Currents | 15 |  | 250 | Conference abstract |
| Barnes, P. and Shankaran, S. and Hintz, S. and Laptook, A. and Higgins, R. and McDonald, S. | 2012 | Magnetic resonance imaging (MRI) in a trial of therapeutic hypothermia for term hypoxic-ischemic encephalopathy (HIE) | Pediatric Radiology | 42 |  | S276-S277 | Conference abstract |
| Bartha, A. I. and Foster-Barber, A. and Miller, S. P. and Vigneron, D. B. and Glidden, D. V. and Barkovich, A. J. and Ferriero, D. M. | 2004 | Neonatal encephalopathy: Association of cytokines with MR spectroscopy and outcome | Pediatric Research | 56 | 6 | 960-966 | No feeding outcomes reported |
| Battin, Malcolm Richard | 2008 | Selective cerebral hypothermia for term infants following hypoxic ischaemic injury |  |  |  |  | No feeding outcomes reported |
| Battin, M. R. and Anne Dezoete, J. and Gunn, T. R. and Gluckman, P. D. and Gunn, A. J. | 2001 | Neurodevelopmental outcome of infants treated with head cooling and mild hypothermia after perinatal asphyxia | Pediatrics | 107 | 3 | 480-484 | No feeding outcomes reported |
| Baumgart, S. and Massaro, A. N. and Chang, T. and Glass, P. and Tsuchida, T. and Short, B. L. | 2011 | Whole body cooling for treatment of and antecedents of neonatal encephalopathy: The Children's National Medical Center's experience May 2006-August 2009, -95 infants | Journal of Neonatal-Perinatal Medicine | 4 | 3 | 201-209 | No analysis of factors influencing HIE feeding outcomes |
| Belet, N. and Belet, U. and Incesu, L. and Uysal, S. and Ozinal, S. and Keskin, T. and Sunter, A. T. and Kucukoduk, S. | 2004 | Hypoxic-ischemic encephalopathy: Correlation of serial MRI and outcome | Pediatric Neurology | 31 | 4 | 267-274 | No feeding outcomes reported |
| Beltempo, Marc and Wintermark, Pia and Mohammad, Khorshid and Jabbour, Elias and Afifi, Jehier and Shivan and a, S and esh and Louis, Deepak and Redpath, Stephanie and Lee, Kyong-Soon and Fajardo, Carlos and Shah, Prakesh S. and Canadian Neonatal Network, Investigators | 2022 | Variations in practices and outcomes of neonates with hypoxic ischemic encephalopathy treated with therapeutic hypothermia across tertiary NICUs in Canada | Journal of perinatology : official journal of the California Perinatal Association | 42 | 7 | 898-906 | No analysis of factors influencing HIE feeding outcomes |
| Benz, Laura D. and Bode, Peter K. and Br and t, Simone and Grass, Beate and Hagmann, Cornelia and Liamlahi, Rabia and Frey, Bernhard and Held, Ulrike and Brotschi, Barbara | 2022 | Placental findings are not associated with neurodevelopmental outcome in neonates with hypoxic-ischemic encephalopathy - an 11-year single-center experience | Journal of perinatal medicine | 50 | 3 | 343-350 | No analysis of breastfeeding or lactation outcomes |
| Berber Çiftci et al. | 2024 | Effect of Nonnutritive Sucking on Oral Feeding in Neonates With Perinatal Asphyxia: A Randomized Controlled Trial | American Journal of Speech-Language Pathology | 33 | 1 | 406-417 | Mixed cohort |
| Bhagat, I. and Agarwal, P. and Leikert, S. and Dechert, R. and Altinok, D. | 2020 | Does severity of brain injury on MRI predict short term outcome in neonates who received therapeutic cooling for hypoxic-ischemic encephalopathy (HIE)? | Pediatrics | 146 | 1 | 341-343 | Conference abstract |
| Bhagat, I. and Agarwal, P. and Sarkar, A. and Dechert, R. and Altinok, D. and Chouthai, N. | 2021 | Does Severity of Brain Injury on Magnetic Resonance Imaging Predict Short-Term Outcome in Neonates Who Received Therapeutic Hypothermia? | American Journal of Perinatology |  |  |  | No analysis of factors influencing HIE feeding outcomes |
| Bhat, M. A. and Charoo, B. A. and Bhat, J. I. and Ahmad, S. M. and Ali, S. W. and Mufti, M. | 2009 | Magnesium sulfate in severe perinatal asphyxia: a randomized, placebo-controlled trial | Pediatrics | 123 | 5 | e764-9 | No analysis of breastfeeding or lactation outcomes |
| Bhat, M. A. and Charoo, B. A. and Bhat, J. I. and Ahmad, S. M. and Ali, S. W. and Mufti, M. U. H. | 2009 | Magnesium sulfate in severe perinatal asphyxia: A randomized, placebo-controlled trial | Obstetrical and Gynecological Survey | 64 | 9 | 573-574 | Editorial paper |
| Bhati, Parul and Sharma, Suvasini and Jain, Ridhimaa and Rath, B. and Beri, Sarita and Gupta, Vinod and Aneja, Satinder | 2019 | Cerebral palsy in North Indian children: Clinico-etiological profile and co-morbidities | Journal of Pediatric Neurosciences | 14 | 1 | 30-35 | Mixed cohort |
| Bosnjak-Nad, K. and Miocinovic, L. P. and Stanojevic, M. and Bosnjak, V. M. and Ahmetasevic, S. G. and Razum, S. and Nad, I. and Hafner, T. and Bosnjak, A. P. | 2012 | Neurodevelopmental outcome of late preterm infants | Gynaecologia et Perinatologia | 21 |  | 32-38 | No feeding outcomes reported |
| Bourel-ponchel, E. and Wallois, F. and Flamein, F. and Lamblin, M. D. | 2019 | P02-F Early EEG characteristics in hypoxic-ischemic encephalopathy and outcome at 2years | Clinical Neurophysiology | 130 | 7 | e67 | Conference abstract |
| Bozkaya, A. and Gunes, A. O. and Ciftci, H. B. and Davutoglu, S. | 2023 | The efficacy of oral motor interventions on feeding outcomes in newborns with hypoxic-ischemic encephalopathy who received therapeutic hypothermia | Turkish Journal of Pediatrics | 65 | 6 | 949 EP - 958 | No analysis of breastfeeding or lactation outcomes |
| Brankica, V. and Svjetlana, M. D. and Miroslava, G. and Sanja, S. | 2012 | The role of oxidative stress in perinatal hypoxic-ischemic brain injury | Srpski Arhiv za Celokupno Lekarstvo | 140 | 1 | 35-41 | No feeding outcomes reported |
| Braun, M. A. and Palmer, M. M. | 1985 | A pilot study of oral-motor dysfunction in “at-risk" infants | Physical and Occupational Therapy in Pediatrics | 5 | 4 | 13-26 | No analysis of factors influencing HIE feeding outcomes |
| Brocard, Clementine and Belaroussi, Yaniss and Labat, Justine and Delmas, Jean and Brissaud, Olivier and Chateil, Jean-Francois | 2021 | Brain MRI after therapeutic hypothermia in asphyxiated newborns: Predictive value at one year of imaging features | European journal of radiology | 139 |  | 109724 | No feeding outcomes reported |
| Brown, J. K. and Purvis, R. J. and Forfar, J. O. and Cockburn, F. | 1974 | Neurological Aspects of Perinatal Asphyxia | Developmental Medicine & Child Neurology | 16 | 5 | 567-580 | Not definitive HIE diagnosis |
| Brugnaro, B. H. and De Abreu, R. W. F. and Verderio, B. N. and Lima, C. R. G. and Dos Santos, M. M. and Rocha, N. A. C. F. | 2022 | Characterization of participation at home of infants with and without biological risk in the first year of life | Developmental Medicine and Child Neurology | 64 |  | 50 | Conference abstract |
| Buchiboyina, Ashok and Ma, Eric and Yip, Andrew and Wagh, Deepika and Tan, Jason and McMichael, Judy and Bulsara, Max and Rao, Shripada | 2017 | Servo controlled versus manual cooling methods in neonates with hypoxic ischemic encephalopathy | Early human development | 112 |  | 35-41 | No feeding outcomes reported |
| Burgod, C. and Mazlan, M. and Pant, S. and Krishnan, V. and Garegrat, R. and Montaldo, P. and Muraleedharan, P. and Bandiya, P. and Kamalaratnam, C. N. and Chandramohan, R. and Manerkar, S. and Jahan, I. and Moni, S. C. and Shahidullah, M. and Rodrigo, R. and Sumanasena, S. and Sujatha, R. and Sathyanathan, B. P. and Joshi, A. R. and Pressler, R. R. and Bassett, P. and Shankaran, S. and Thayyil, S. | 2024 | Duration of birth depression and neurodevelopmental outcomes after whole-body hypothermia for hypoxic ischemic encephalopathy in India, Sri Lanka and Bangladesh - an exploratory analysis of the HELIX trial | The Lancet Regional Health - Southeast Asia | 2772-3682 (electronic) 2772-3682 | 20 | 100284 | No feeding outcomes reported |
| Burt, Alan J. | 1984 | Study of symptomatic birth asphyxia : its immediate manifestations and long term significance |  |  |  |  | No analysis of factors influencing HIE feeding outcomes |
| Byrne, P. and Welch, R. and Johnson, M. A. and Darrah, J. and Piper, M. | 1990 | Serial magnetic resonance imaging in neonatal hypoxic-ischemic encephalopathy | The Journal of Pediatrics | 117 | 5 | 694-700 | No feeding outcomes reported |
| Calabrese, Evan and Wu, Yvonne and Scheffler, Aaron Wolfe and Wisnowski, Jessica L. and McKinstry, Robert C. and Mathur, Amit and Glass, Hannah C. and Comstock, Bryan A. and Heagerty, Patrick J. and Gillon, Shivani and Juul, Sandra E. and Hess, Christopher P. and Li, Yi | 2023 | Correlating Quantitative MRI-based Apparent Diffusion Coefficient Metrics with 24-month Neurodevelopmental Outcomes in Neonates from the HEAL Trial | Radiology | 308 | 3 | e223262 | No feeding outcomes reported |
| Calame, A. and Reymond-Goni, I. and Maherzi, M. and Roulet, M. and March and , C. and Prod'hom, L. S. | 1976 | Psychological and neurodevelopmental outcome of high risk newborn infants | Helvetica paediatrica acta | 31 | 4 | 287-97 | No feeding outcomes reported |
| Calkavur, S. and Akisu, M. and Olukman, O. and Balim, Z. and Berdeli, A. and Cakmak, B. and Koroglu, O. and Yalaz, M. and Kultursay, N. | 2011 | Genetic factors that influence short-term neurodevelopmental outcome in term hypoxic-ischaemic encephalopathic neonates | The Journal of international medical research | 39 | 5 | 1744-56 | No feeding outcomes reported |
| Calkavur, S. and Olukman, O. and Koroglu, O. and Yalaz, M. and Balim, Z. and Berdeli, A. and Akisu, M. and Kultursay, N. | 2010 | Importance of cytokines gene polymorphisms and thrombophilic states on short-termneurodevelopmental outcome in term hypoxic-ischaemic encephalopathic neonates | Early Human Development | 86 |  | S11-S12 | Conference abstract |
| Calvo Medina, R. and Pelaez Cantero, M. J. and Rodriguez, A. Madrid and Romina, A. B. and Moreno Medinilla, E. and Mora Ramirez, M. D. and Martinez Anton, J. and Urda Cardona, A. | 2015 | Palliative care and paediatric neurology | European Journal of Paediatric Neurology | 19 |  | S127 | Conference abstract |
| Cardona, V. Q. and Rao, R. and Zaniletti, I. and Joe, P. and Johnson, Y. R. and DiGeronimo, R. and Hamrick, S. E. and Lee, K. S. and Mietzsch, U. and Natarajan, G. and Peeples, E. S. and Wu, T. W. and Hossain, T. and Flibotte, J. and Ch and el, A. and Distler, A. and Shenberger, J. S. and Oghifobibi, O. and Massaro, A. N. and Dizon, M. L. V. | 2023 | Association of Hospital Resource Utilization with Neurodevelopmental Outcomes in Neonates with Hypoxic-Ischemic Encephalopathy | JAMA Network Open | 6 | 3 | e233770 | No analysis of factors influencing HIE feeding outcomes |
| Carli, G. and Reiger, I. and Evans, N. | 2004 | One-year neurodevelopmental outcome after moderate newborn hypoxic ischaemic encephalopathy | Journal of Paediatrics and Child Health | 40 | 4 | 217-220 | No feeding outcomes reported |
| Catherine, R. Christina and Bhat, B. Vishnu and Adhisivam, B. and Bharadwaj, Shruthi K. and Vinayagam, Vickneshwaran and Chinnakali, Palanivel | 2020 | Neuronal Biomarkers in Predicting Neurodevelopmental Outcome in Term Babies with Perinatal Asphyxia | Indian journal of pediatrics | 87 | 10 | 787-792 | No feeding outcomes reported |
| Celik, Y. and Ozgur, A. and Sungur, M. A. and Ylldlrlm, N. and Teke, S. | 2023 | Is Selective Head Cooling Combined with Whole-Body Cooling the Most Effective Hypothermia Method for Neonatal Hypoxic-Ischemic Encephalopathy? | Therapeutic Hypothermia and Temperature Management | 13 | 2 | 70-76 | No analysis of breastfeeding or lactation outcomes |
| Chalak, Lina F. and Pappas, Athina and Tan, Sylvia and Das, Abhik and Sanchez, Pablo J. and Laptook, Abbot R. and Van Meurs, Krisa P. and Shankaran, Seetha and Bell, Edward F. and Davis, Alexis S. and Heyne, Roy J. and Pedroza, Claudia and Poindexter, Brenda B. and Schibler, Kurt and Tyson, Jon E. and Ball, M. Bethany and Bara, Rebecca and Grisby, Cathy and Sokol, Gregory M. and D'Angio, Carl T. and Hamrick, Shannon E. G. and Dysart, Kevin C. and Cotten, C. Michael and Truog, William E. and Watterberg, Kristi L. and Timan, Christopher J. and Garg, Meena and Carlo, Waldemar A. and Higgins, Rosemary D. and Eunice Kennedy Shriver National Institute of Child, Health and Human Development Neonatal Research, Network | 2021 | Association Between Increased Seizures During Rewarming After Hypothermia for Neonatal Hypoxic Ischemic Encephalopathy and Abnormal Neurodevelopmental Outcomes at 2-Year Follow-up: A Nested Multisite Cohort Study | JAMA neurology | 78 | 12 | 1484-1493 | No feeding outcomes reported |
| Chalak, L. F. and Prempunpong, C. and Garfinkle, J. and Rollins, N. and Nguyen, K. A. and Pappas, A. and Montaldo, P. and Thayyil, S. and Sanchez, P. J. and Shankaran, S. and Laptook, A. R. and Sant'Anna, G. | 2017 | Prospective study of infants with mild encephalopathy: Prime study | Acta Paediatrica, International Journal of Paediatrics | 106 |  | 9 | No analysis of factors influencing HIE feeding outcomes |
| Chalak, Lina F. and Rollins, Nancy and Morriss, Michael C. and Brion, Luc P. and Heyne, Roy and Sanchez, Pablo J. | 2012 | Perinatal acidosis and hypoxic-ischemic encephalopathy in preterm infants of 33 to 35 weeks' gestation | The Journal of pediatrics | 160 | 3 | 388-94 | Mixed cohort |
| Chang, Lilly L. and Wynn, James L. and Pacella, Marisa J. and Rossignol, C and ace C. and Banadera, Felix and Alviedo, Neil and Vargas, Alfonso and Bennett, Jeffrey and Huene, Melissa and Copenhaver, Nicole and Sura, Livia and Barnette, Kimberly and Solomon, Jayne and Bliznyuk, Nikolay A. and Neu, Josef and Weiss, Michael D. | 2018 | Enteral Feeding as an Adjunct to Hypothermia in Neonates with Hypoxic-Ischemic Encephalopathy | Neonatology | 113 | 4 | 347-352 | No analysis of breastfeeding or lactation outcomes |
| Chang, Peter D. and Chow, Daniel S. and Alber, Anna and Lin, Yen-Kuang and Youn, Young Ah | 2020 | Predictive Values of Location and Volumetric MRI Injury Patterns for Neurodevelopmental Outcomes in Hypoxic-Ischemic Encephalopathy Neonates | Brain sciences | 10 | 12 |  | No analysis of factors influencing HIE feeding outcomes |
| Charki, S. and Kalyanshettar, S. and Singh, S. and Biradar, V. and Kulkarni, T. and Patil, S. | 2020 | Experience of therapeutic hypothermia in neonates with perinatal asphyxia in a tertiary care center in North Karnataka, India | Journal of Clinical Neonatology | 9 | 3 | 175-181 | No feeding outcomes reported |
| Charki, S. and Patil, S. V. and Vijayakumar, S. and Kolkar, Y. | 2024 | Erythropoietin in Neonates with Perinatal Asphyxia Undergoing Therapeutic Hypothermia-A Prospective Cohort Study | Journal of Neonatology | 38 | 2 | 302 | No feeding outcomes reported |
| Charon, Valerie and Proisy, Maia and Bretaudeau, Gilles and Bruneau, Bertr and Pladys, Patrick and Beuchee, Alain and Burnouf-Rose, Gladys and Ferre, Jean-Christophe and Rozel, Celine | 2016 | Early MRI in neonatal hypoxic-ischaemic encephalopathy treated with hypothermia: Prognostic role at 2-year follow-up | European journal of radiology | 85 | 8 | 1366-74 | No feeding outcomes reported |
| Chattopadhyay, N and ita and Mitra, Kaninika | 2015 | Neurodevelopmental outcome of high risk newborns discharged from special care baby units in a rural district in India | Journal of public health research | 4 | 1 | 318 | No feeding outcomes reported |
| Chen, D. Y. and Lee, I. C. and Yu, C. S. and Wong, S. H. and Lue, K. H. | 2021 | Troponin i levels in neonatal hypoxic-ischemic encephalopathy are related to cardiopulmonary comorbidity and neurodevelopmental outcomes | Journal of Clinical Medicine | 10 | 17 | 4010 | No feeding outcomes reported |
| Cheung, K. and Janice, I. P. and Leeyuen, K. O. and Law, M. and Lam, W. | 2016 | Correlation with MR findings in neonates with hypoxic ischaemic encephalopathy undergoing cerebral hypothermia and subsequent neurolodevelopmental outcomes | Pediatric Radiology | 46 |  | S106 | Conference abstract |
| Chiang, M. C. and Lien, R. and Lin, J. J. and Lin, K. L. and Chu, S. M. and Yang, P. H. and Wang, H. S. | 2012 | Serum S100B protein and short-term outcome of newborn infants with hypoxic-ischemic encephalopathy treated with moderate hypothermia | Developmental Medicine and Child Neurology | 54 |  | 195-196 | Conference abstract |
| Chiesa, C. and Pellegrini, G. and Panero, A. and De Luca, T. and Assumma, M. and Signore, F. and Pacifico, L. | 2003 | Umbilical cord interleukin-6 levels are elevated in term neonates with perinatal asphyxia | European Journal of Clinical Investigation | 33 | 4 | 352-358 | No feeding outcomes reported |
| Chouthai, Nitin S. and Sobczak, Holly and Khan, Reshma and Subramanian, Divya and Raman, Sim and Rao, Raghavendra | 2015 | Hyperglycemia is associated with poor outcome in newborn infants undergoing therapeutic hypothermia for hypoxic ischemic encephalopathy | Journal of neonatal-perinatal medicine | 8 | 2 | 125-31 | No feeding outcomes reported |
| Chuang, S. L. and Gbinigie, H. and Shivamurthappa, V. and Ogundipe, E. | 2016 | Outcomes of high risk surgical neonatal cohort up to 18 months at a UK tertiary perinatal referral surgical unit | Journal of Maternal-Fetal and Neonatal Medicine | 29 |  | 228 | Conference abstract |
| Cizmeci, M. N. and Sarica, C. and Kalish, B. T. and Lozano, A. M. and Chen, R. | 2024 | Neuromodulation using transcranial focused ultrasonography in neonates with perinatal hypoxic-ischemic encephalopathy | Medical Hypotheses | 191 |  | 111463 | Protocol or trial registry entry |
| Cornet, Marie-Coralie and Gonzalez, Fernando F. and Glass, Hannah C. and Wu, Tai-Wei and Wisnowski, Jessica L. and Li, Yi and Heagerty, Patrick and Juul, Sandra E. and Wu, Yvonne W. | 2025 | Chorioamnionitis and Two-Year Outcomes in Infants with Hypoxic-Ischemic Encephalopathy | The Journal of Pediatrics | 278 |  | 114415 | No feeding outcomes reported |
| Costescu, O. C. and Manea, A. M. and Boia, E. R. and Cioboata, D. M. and Doandes, F. M. and Enatescu, I. and Costescu, S. and Prodan, M. and Boia, M. | 2024 | Early Postnatal Administration of Erythropoietin and Its Association with Neurodevelopmental Outcomes and Incidence of Intraventricular Hemorrhage and Hypoxic-Ischemic Encephalopathy: A Four-Week Observational Study | Pediatric Reports | 16 | 2 | 339 EP - 352 | No feeding outcomes reported |
| Cotten, C. Michael and Murtha, Amy P. and Goldberg, Ronald N. and Grotegut, Chad A. and Smith, P. Brian and Goldstein, Ricki F. and Fisher, Kimberley A. and Gustafson, Kathryn E. and Waters-Pick, Barbara and Swamy, Geeta K. and Rattray, Benjamin and Tan, Siddhartha and Kurtzberg, Joanne | 2014 | Feasibility of autologous cord blood cells for infants with hypoxic-ischemic encephalopathy | The Journal of pediatrics | 164 | 5 | 973-979.e1 | No analysis of breastfeeding or lactation outcomes |
| Coutinho, K. A. A. and de Pacheco, S. T. A. and Rodrigues, B. M. R. D. and Da Silva, L. F. | 2015 | Family home care in feeding children with encephalopathy | Revista Enfermagem | 23 | 3 | 318-323 | No analysis of factors influencing HIE feeding outcomes |
| CTRI | 2024 | Evaluation of the adequacy of spoon feeding in neonates who have not cried at birth and suffered subsequent brain malfunction | https://trialsearch.who.int/Trial2.aspx?TrialID=CTRI/2024/08/071881 |  |  |  | Protocol or trial registry entry |
| Curtis, P. D. and Matthews, T. G. and Clarke, T. A. and Darling, M. and Crowley, P. and Griffin, E. and O'Connell, P. and Gorman, W. and O'Brien, N. and O'Herlihy, C. and O'Regan, M. | 1988 | Neonatal seizures: The Dublin Collaborative Study | Archives of Disease in Childhood | 63 | 9 | 1065-1068 | No analysis of factors influencing HIE feeding outcomes |
| da Silva, L. F. G. and Filho, J. R. H. and AnÃ©s, M. and Nunes, M. L. | 2006 | Prognostic Value of 1H-MRS in Neonatal Encephalopathy | Pediatric Neurology | 34 | 5 | 360-366 | No feeding outcomes reported |
| Dalmazzo, C. and Pomero, G. and Delogu, A. and Alpicrovi, C. and Borgarello, G. and Castellino, N. and Simonitti, A. and Gancia, P. | 2011 | Correlation between MRI and neurological outcome at 1 year of age in asphyxiated cooled infants | Journal of Maternal-Fetal and Neonatal Medicine | 24 |  | 168-169 | Conference abstract |
| Damien, J. and Vannasing, P. and Tremblay, J. and Petitpas, L. and Marandyuk, B. and Balasingam, T. and El Jalbout, R. and Paquette, N. and Donofrio, G. and Birca, A. and Gallagher, A. and Pinchefsky, E. F. | 2024 | Relationship between EEG spectral power and dysglycemia with neurodevelopmental outcomes after neonatal encephalopathy | Clinical Neurophysiology | 163 |  | 160-173 | No feeding outcomes reported |
| Danguecan, Ashley and El Shahed, Amr I. and Somerset, Emily and Fan, Chun-Po Steve and Ly, Linh G. and Williams, Tricia | 2021 | Towards a biopsychosocial understanding of neurodevelopmental outcomes in children with hypoxic-Ischemic encephalopathy: A mixed-methods study | The Clinical Neuropsychologist | 35 | 5 | 925-947 | No feeding outcomes reported |
| Davis, D. J. and Creery, W. D. and Radziuk, J. | 1999 | Inappropriately high plasma insulin levels in suspected perinatal asphyxia | Acta Paediatrica, International Journal of Paediatrics | 88 | 1 | 76-81 | No feeding outcomes reported |
| De Vries, L. S. and Pierrat, V. and Eken, P. and Minami, T. and Daniels, H. and Casaer, P. | 1991 | Prognostic value of early somatosensory evoked potentials for adverse outcome in full-term infants with birth asphyxia | Brain and Development | 13 | 5 | 320-325 | No feeding outcomes reported |
| Del Balzo, Francesca and Maiolo, Stella and Papoff, Paola and Giannini, Luigi and Moretti, Corrado and Properzi, Enrico and Spalice, Alberto | 2014 | Electroencephalogram and magnetic resonance imaging comparison as a predicting factor for neurodevelopmental outcome in hypoxic ischemic encephalopathy infant treated with hypothermia | Pediatric reports | 6 | 3 | 5532 | Feeding as part of a composite outcome |
| Delnard, N. and Cneude, F. and Hamelin, S. and Emeriaud, G. and Berne-Audeoud, F. and Andrini, P. and Debillon, T. | 2010 | [Assessment of a hypothermia protocol implementation for hypoxic-ischemic encephalopathy in term newborns] | Evaluation d'un protocole de prise en charge de l'encephalopathie anoxo-ischemique du nouveau-ne par hypothermie. | 17 | 10 | 1425-32 | No analysis of breastfeeding or lactation outcomes |
| Deorari, A. K. and Paul, V. K. and Singh, M. | 1989 | Birth asphyxia and neurodevelopmental outcome | Indian pediatrics | 26 | 8 | 793-9 | Not definitive HIE diagnosis |
| Dereymaeker, Anneleen and Matic, Vladimir and Vervisch, Jan and Cherian, Perumpillichira J. and Ansari, Amir H. and De Wel, Ofelie and Govaert, Paul and De Vos, Maarten and Van Huffel, Sabine and Naulaers, Gunnar and Jansen, Katrien | 2019 | Automated EEG background analysis to identify neonates with hypoxic-ischemic encephalopathy treated with hypothermia at risk for adverse outcome: A pilot study | Pediatrics and neonatology | 60 | 1 | 50-58 | No feeding outcomes reported |
| Dhungana, S. P. and Shrestha, S. and Shrestha, G. S. | 2020 | Correlation of Thompson Score in Predicting Early Outcome of Newborn with Birth Asphyxia | Journal of Nepal Health Research Council | 18 | 3 | 406-410 | No feeding outcomes reported |
| Diaz Martinez, C. | 2010 | Early sequential electroencephalogram (EEG) in neonates with hypoxic ischemic encephalopathy (HIE) | Clinical Neurophysiology | 121 |  | S252 | Conference abstract |
| Dimario, F. J., Jr. | 1989 | Symmetrical Thalamic Degeneration With Calcifications of Infancy | American Journal of Diseases of Children | 143 | 9 | 1056-1060 | <3 participants meeting criteria |
| Doandes, Florina Marinela and Manea, Aniko Maria and Lungu, Nicoleta and Br and ibur, Timea and Cioboata, Daniela and Costescu, Oana Cristina and Zaharie, Mihaela and Boia, Marioara | 2023 | The Role of Amplitude-Integrated Electroencephalography (aEEG) in Monitoring Infants with Neonatal Seizures and Predicting Their Neurodevelopmental Outcome | Children | 10 | 5 | 833 | No feeding outcomes reported |
| Dorrepaal, C. A. and Berger, H. M. and Benders, Mjnl and vanZoerenGrobben, D. and VanDeBor, M. and VanBel, F. | 1996 | Nonprotein-bound iron in postasphyxial reperfusion injury of the newborn | Pediatrics | 98 | 5 | 883-889 | No feeding outcomes reported |
| Doughty, Kimberly N. and Nichols, Caitlin and Henry, Christine and Shabanova, Veronika and Taylor, Sarah N. | 2024 | Maternal stress and breastfeeding outcomes in the NICU couplet care experience: a prospective cohort study | Journal of Perinatology | 44 | 11 | 1624-1629 | Wrong population |
| Doyle, O. M. and Greene, B. R. and Murray, D. M. and Marnane, L. and Lightbody, G. and Boylan, G. B. | 2007 | The effect of frequency band on quantitative EEG measures in neonates with hypoxic-ischaemic encephalopathy | Annual International Conference of the IEEE Engineering in Medicine and Biology Society. IEEE Engineering in Medicine and Biology Society. Annual International Conference | 2007 |  | 717-21 | No feeding outcomes reported |
| Doyle, O. M. and Temko, A. and Murray, D. M. and Lightbody, G. and Marnane, W. and Boylan, G. B. and Ieee and Biol, Soc and Med, Ieee Engn | 2010 | Predicting the neurodevelopmental outcome in newborns with hypoxic-ischaemic injury | 32nd Annual International Conference of the IEEE Engineering-in-Medicine-and-Biology-Society (EMBC 10) |  |  | 1370-1373 | No feeding outcomes reported |
| Dunne, J. M. and Wertheim, D. and Clarke, P. and Kapellou, O. and Chisholm, P. and Boardman, J. P. and Shah, D. K. | 2016 | Automated electroencephalographic discontinuity in cooled newborns predicts cerebral MRI and neurodevelopmental outcome | Archives of Disease in Childhood |  |  |  | No feeding outcomes reported |
| DuPont, Tara L. and Chalak, Lina F. and Morriss, Michael C. and Burchfield, P. Jeannette and Christie, Lucy and Sanchez, Pablo J. | 2013 | Short-term outcomes of newborns with perinatal acidemia who are not eligible for systemic hypothermia therapy | The Journal of Pediatrics | 162 | 1 | 35-41 | No analysis of breastfeeding or lactation outcomes |
| Edney, Sarah K. and Basu, Anna and Harding, Celia and Pennington, Lindsay | 2022 | Short-term feeding outcomes after neonatal brain injury | Journal of Neonatal Nursing | 28 | 4 | 265-269 | Mixed cohort |
| Eken, P. and Toet, M. C. and Groenendaal, F. and De Vries, L. S. | 1995 | Predictive value of early neuroimaging, pulsed Doppler and neurophysiology in full term infants with hypoxic-ischaemic encephalopathy | Archives of Disease in Childhood | 73 | 2 | F75-F80 | No feeding outcomes reported |
| El-Halim, S. A. A. | 2022 | Recording of Complications of Treatment of Hypoxic Ischemic Neonates by Passive Whole-Body Cooling: A Study in Neonatal Intensive Care Unit of Mataria Teaching Hospital | Egyptian Journal of Hospital Medicine | 88 | 1 | 2625-2633 | No analysis of factors influencing HIE feeding outcomes |
| Ellis, Matthew and Man and har, Nilu and Shrestha, Prakash S. and Shrestha, Laxman and Man and har, Dharma S. and De L Costello, Anthony M. and Ellis, M. and Man and har, N. and Shrestha, P. S. and Shrestha, L. and Man and har, D. S. and Costello, A. M. | 1999 | Outcome at 1 year of neonatal encephalopathy in Kathmandu, Nepal | Developmental Medicine & Child Neurology | 41 | 10 | 689-695 | No analysis of factors influencing HIE feeding outcomes |
| Escrig Fern and ez, R. and Gimeno Navarro, A. and Cernada Badia, M. and Boronat Gonzalez, N. and Alberola Perez, A. and Vento Torres, M. and Izquierdo Macian, I. | 2015 | Influence of birth location on outcome of newborns with hypoxic ischemic encephalopathy treated with therapeutic hypothermia | Journal of Perinatal Medicine | 43 |  |  | Conference abstract |
| Fall, C. and Baer, R. J. and Jelliffe-Pawlowski, L. and Matoba, N. and Lee, H. C. and Chambers, C. D. and Bandoli, G. | 2024 | Racial and Ethnic Inequities in Therapeutic Hypothermia and Neonatal Hypoxic-Ischemic Encephalopathy: A Retrospective Cohort Study | Journal of Pediatrics | 269 |  | 113966 | No analysis of breastfeeding or lactation outcomes |
| Fall, C. M. and Baer, R. J. and B and oli, G. and Chambers, C. D. | 2023 | RACIAL DISPARITITIES IN THERAPEUTIC HYPOTHERMIA AND ADVERSE OUTCOMES OF HYPOXIC ISCHEMIC ENCEPHALOPATHY IN A LARGE CALIFORNIA NEONATAL COHORT | Journal of Investigative Medicine | 71 | 1 | 442 | Conference abstract |
| Fall, C. M. and Baer, R. J. and B and oli, G. and Chambers, C. D. | 2023 | CHANGES IN TREATMENT AND OUTCOMES IN LATE PRETERM AND TERM INFANTS WITH HYPOXIC ISCHEMIC ENCEPHALOPATHY OVER TIME IN A LARGE CALIFORNIA NEONATAL COHORT | Journal of Investigative Medicine | 71 | 1 | 74 | Conference abstract |
| Ferradal, S. L. and Vyas, R. and El-Dib, M. and Sutin, J. and Pierce, L. and Morton, S. and Soul, J. and Lin, P. Y. and Ellen Grant, P. | 2020 | Longitudinal monitoring of cerebral metabolism in neonates at risk for hypoxic-ischemic encephalopathy C3 - Optics InfoBase Conference Papers |  |  |  |  | Conference abstract |
| Foster-Barber, A. and Dickens, B. and Ferriero, D. M. | 2001 | Human perinatal asphyxia: Correlation of neonatal cytokines with MRI and outcome | Developmental Neuroscience | 23 | 3 | 213-218 | No feeding outcomes reported |
| Frank, C. M. C. and Nikkels, P. G. J. and Harteman, J. C. and van Haastert, I. C. and Benders, Mjnl and Koopman-Esseboom, C. and de Vries, L. S. and Groenendaal, F. | 2016 | Placental pathology and outcome after perinatal asphyxia and therapeutic hypothermia | Journal of Perinatology | 36 | 11 | 977-984 | No feeding outcomes reported |
| Fukuda, Sumio and Tanimura, Tomoshige and Iwaki, Toshihiko and Higuchi, Machiko and Suyama, Megumi and Goto, Tomoki and Koide, Wakato and Maki, Kanemasa and Ushijima, Katsumi and Ban, Kyoko | 2016 | Ultrasonography of the internal carotid artery during therapeutic hypothermia | Pediatrics international : official journal of the Japan Pediatric Society | 58 | 7 | 666-8 | No feeding outcomes reported |
| Funato, M. and Maruta, K. and Yano, M. and Kai, M. and Umezawa, Y. and Yasuda, K. and Ohta-Noda, E. and Gen, K. | 2023 | Efficacy of interferential current transcutaneous electrical sensory stimulation through the neck skin for treating dysphagia in children with disabilities: A case series | SAGE Open Medical Case Reports | 11 |  |  | Mixed cohort |
| Gaillot, K. and Maurin, L. and Bertr and , P. and Fakhri, N. and Saliba, E. and Sirinelli, D. | 2015 | Early diffusion weighted imaging versus T1 and T2 imaging to predict medium-term outcome after moderate hypothermia for neonatal hypoxic-ischemic encephalopathy | Reanimation | 24 | 5 | 573-585 | Feeding as part of a composite outcome |
| Galderisi, A. and Tordin, M. and Suppiej, A. and Cainelli, E. and Baraldi, E. and Trevisanuto, D. | 2023 | Glucose-to-lactate ratio and neurodevelopment in infants with hypoxic-ischemic encephalopathy: an observational study | European Journal of Pediatrics | 182 | 2 | 837 EP - 844 | No feeding outcomes reported |
| Gale, Chris and Longford, Nicholas T. and Jeyakumaran, Dusha and Ougham, Kayleigh and Battersby, Cheryl and Ojha, Shalini and Dorling, Jon | 2021 | Feeding during neonatal therapeutic hypothermia, assessed using routinely collected National Neonatal Research Database data: a retrospective, UK population-based cohort study | The Lancet. Child & adolescent health | 5 | 6 | 408-416 | Duplicate of data already included |
| Gardiner, J. and Wagh, D. and McMichael, J. and Hakeem, M. and Rao, S. | 2013 | Two year neurodevelopmental outcomes of neonates with hypoxic ischemic encephalopathy treated with therapeutic hypothermia using cool gel packs-an experience from western australia | Journal of Paediatrics and Child Health | 49 |  | 98-99 | Conference abstract |
| Gardiner, Jackie and Wagh, Deepika and McMichael, Judy and Hakeem, Mohammed and Rao, Shripada | 2014 | Outcomes of hypoxic ischaemic encephalopathy treated with therapeutic hypothermia using cool gel packs - experience from Western Australia | European journal of paediatric neurology : EJPN : official journal of the European Paediatric Neurology Society | 18 | 3 | 391-8 | No analysis of factors influencing HIE feeding outcomes |
| Garfinkle, Jarred and Sant'Anna, Guilherme Mendes and Wintermark, Pia and Ali, Nabeel and Morneault, Linda and Koclas, Louise and Shevell, Michael I. | 2013 | Cooling in the real world: therapeutic hypothermia in hypoxic-ischemic encephalopathy | European journal of paediatric neurology : EJPN : official journal of the European Paediatric Neurology Society | 17 | 5 | 492-7 | No feeding outcomes reported |
| Girard, N. and Millet, V. and Bartoli, J. M. and Lacroze, V. and Leboucq, N. and Raybaud, C. and Unal, D. | 1997 | Prognostic value of magnetic resonance imaging at 3 to 6 months corrected chronological age in infants who suffered perinatal neurologic insult | International Journal of Neuroradiology | 3 | 1 | 57-67 | No feeding outcomes reported |
| Gire, C. and Nicaise, C. and Roussel, M. and Soula, F. and Girard, N. and Somma-Mauvais, H. and Lagier, P. and Dejode, J. M. and Farnarier, G. and Garnier, J. M. | 2000 | [Hypoxic-ischemic encephalopathy in the full-term newborn. Contribution of electroencephalography and MRI or computed tomography to its prognostic evaluation. Apropos of 26 cases] | Encephalopathie hypoxo-ischemique du nouveau-ne a terme. Apport de l'electroencephalogramme et de l'IRM ou de la TDM a l'evaluation pronostique. A propos de 26 observations. | 30 | 2 | 97-107 | No feeding outcomes reported |
| Gluckman, Peter D. and Wyatt, John S. and Azzopardi, Denis and Ballard, Roberta and Edwards, A. David and Ferriero, Donna M. and Polin, Richard A. and Robertson, Charlene M. and Thoresen, Marianne and Whitelaw, Andrew and Gunn, Alistair J. | 2005 | Selective head cooling with mild systemic hypothermia after neonatal encephalopathy: Multicentre randomised trial | The Lancet | 365 | 9460 | 663-670 | No feeding outcomes reported |
| Goeral, K. and Giordano, V. and Klebermass-Schrehof, K. and Weninger, M. and Berger, A. and Olischar, M. | 2014 | Effect of hypothermia on amplitude-integrated electroencephalogram in infants with perinatal asphyxia | Archives of Disease in Childhood | 99 |  | A373 | Conference abstract |
| Gonzalez, Fernando F. and Voldal, Emily and Comstock, Bryan A. and Mayock, Dennis E. and Goodman, Amy M. and Cornet, Marie-Coralie and Wu, Tai-Wei and Redline, Raymond W. and Heagerty, Patrick and Juul, Sandra E. and Wu, Yvonne W. | 2023 | Placental Histologic Abnormalities and 2-Year Outcomes in Neonatal Hypoxic-Ischemic Encephalopathy | Neonatology | 120 | 6 | 760-767 | No feeding outcomes reported |
| Goulding, R. M. and Stevenson, N. J. and Murray, D. M. and Livingstone, V. and Boylan, G. B. | 2014 | Heart rate variability in full-term neonates with hypoxic ischaemic encephalopathy | Archives of Disease in Childhood | 99 |  | A396-A397 | Conference abstract |
| Goulding, Robert M. and Stevenson, Nathan J. and Murray, Deirdre M. and Livingstone, Vicki and Filan, Peter M. and Boylan, Geraldine B. | 2015 | Heart rate variability in hypoxic ischemic encephalopathy: correlation with EEG grade and 2-y neurodevelopmental outcome | Pediatric research | 77 | 5 | 681-7 | No feeding outcomes reported |
| Govind, A. and Raghoji, C. and Guruprasad, G. | 2025 | Therapeutic Hypothermia for Birth Asphyxia Using a New Servo-Controlled Device-A Randomised Controlled Trial | Journal of Neonatology |  |  |  | No feeding outcomes reported |
| Grass, Beate and El Shahed, Amr and Ly, Linh G. and Chau, Vann and Branson, Helen M. and Blaser, Susan and Runeckles, Kyle and Wilson, Diane and Whyte, Hilary | 2020 | Therapeutic hypothermia for hypoxic-ischemic encephalopathy after perinatal sentinel events: less brain injury on MRI and improved neurodevelopmental outcome at 18-36 months | Journal of perinatology : official journal of the California Perinatal Association | 40 | 4 | 633-639 | No feeding outcomes reported |
| Grass, B. and Scheidegger, S. and Latal, B. and Hagmann, C. and Held, U. and Brotschi, B. | 2020 | Short-term neurological improvement in neonates with hypoxic-ischemic encephalopathy predicts neurodevelopmental outcome at 18-24 months | Journal of Perinatal Medicine | 48 | 3 | 296-303 | No feeding outcomes reported |
| Grist, L. and MacKie, S. and Vollmer, B. | 2023 | Introduction of a Post Discharge Phone Call for Neonatal Neurology Patients: A Pilot Within Southampton Children's Hospital | Developmental Medicine and Child Neurology | 65 |  | 65 EP - 66 | Conference abstract |
| Groenendaal, F. and Benders, M. J. and De Vries, L. S. | 2006 | Pre-Wallerian Degeneration in the Neonatal Brain Following Perinatal Cerebral Hypoxia-Ischemia Demonstrated with MRI | Seminars in Perinatology | 30 | 3 | 146-150 | No feeding outcomes reported |
| Groenendaal, F. and van der Grond, J. and van Haastert, I. C. and Eken, P. and Mali, W. P. and de Vries, L. S. | 1996 | [Findings in cerebral proton spin resonance spectroscopy in newborn infants with asphyxia, and psychomotor development] | Bevindingen bij cerebrale protonkernspinresonantie-spectroscopie bij pasgeborenen met asfyxie, en psychomotore ontwikkeling. | 140 | 5 | 255-9 | No feeding outcomes reported |
| Grossmann, Katarina Robertsson | 2022 | Long-term Outcome after Hypothermia-Treated Hypoxicischaemic Encephalopathy |  |  |  |  | No feeding outcomes reported |
| Guarnera, Alessia and Lucignani, Giulia and Parrillo, Chiara and Rossi-Espagnet, Maria Camilla and Carducci, Chiara and Moltoni, Giulia and Savarese, Immacolata and Campi, Francesca and Dotta, Andrea and Milo, Francesco and Cappelletti, Simona and Capitello Grimaldi, Teresa and G and olfo, Carlo and Napolitano, Antonio and Longo, Daniela | 2023 | Predictive Value of MRI in Hypoxic-Ischemic Encephalopathy Treated with Therapeutic Hypothermia | Children | 10 | 3 | 446 | No feeding outcomes reported |
| Guillot, Mireille and Philippe, Marissa and Miller, Elka and Davila, Jorge and Barrowman, Nicholas James and Harrison, Mary-Ann and Ben Fadel, Nadya and Redpath, Stephanie and Lemyre, Brigitte | 2019 | Influence of timing of initiation of therapeutic hypothermia on brain MRI and neurodevelopment at 18 months in infants with HIE: a retrospective cohort study | BMJ paediatrics open | 3 | 1 | e000442 | No feeding outcomes reported |
| Guillot, M. and Philippe, M. and Miller, E. and Davila, J. and Borrowman, N. and Harrison, M. A. and Ben Fadel, N. and Redpath, S. and Lemyre, B. | 2018 | Does the timing of initiation of therapeutic hypothermia influence MRI findings and outcomes in encephalopathic babies? | Paediatrics and Child Health (Canada) | 23 |  | e23 | Conference abstract |
| Gulati, I. K. and Shubert, T. and Gao, X. and Jadcherla, S. | 2014 | Proximal aero-digestive and respiratory interactions in infants with hypoxic ischemic encephalopathy (HIE) | Gastroenterology | 146 | 5 | S-894 | Conference abstract |
| Gulati, I. K. and Shubert, T. and Gao, X. and Jadcherla, S. | 2014 | Reactivity of lower esophageal sphincter (LES) upon pharyngeal provocation in infants with hypoxic ischemic encephalopathy (HIE) | Gastroenterology | 146 | 5 | S-101 | Conference abstract |
| Gulati, Ish K. and Shubert, Theresa R. and Sitaram, Swetha and Wei, Lai and Jadcherla, Sudarshan R. | 2015 | Effects of birth asphyxia on the modulation of pharyngeal provocation-induced adaptive reflexes | American journal of physiology. Gastrointestinal and liver physiology | 309 | 8 | G662-9 | No analysis of factors influencing HIE feeding outcomes |
| Gulczynska, E. and Gadzinowski, J. and Walas, W. and Maczka, A. and Talar, T. and Kesiak, M. and Caputa, J. and Sobolewska, B. | 2014 | Therapeutic hypothermia enhanced by MGSO4 for hypoxic-ischemic encephalopathy in the neonates and its influence on biomarkers of asphyxia and course of hospitalization | Journal of Maternal-Fetal and Neonatal Medicine | 27 |  | 221 | Invited lecture |
| Gupta, Suneeti and Bapuraj, Jayapalli Rajiv and Carlson, Gabrielle and Trumpower, Emily and Dechert, Ronald E. and Sarkar, Subrata | 2018 | Predicting the need for home gavage or g-tube feeds in asphyxiated neonates treated with therapeutic hypothermia | Journal of perinatology : official journal of the California Perinatal Association | 38 | 6 | 728-733 | Feeding as part of a composite outcome |
| Hadzimuratovic, E. and Brankovic, S. and Hadzimuratovic, A. and Bukvic, M. | 2022 | Prognostic value of colour Doppler brain sonography for the neurodevelopmental outcome in term neonates with hypoxic ischaemic encephalopathy | Medicinski Glasnik | 19 | 2 | 173-177 | No feeding outcomes reported |
| Hallberg, Boubou | 2010 | Hypoxic-Ischemic Encephalopathy : Diagnosis, Hypothermia Treatment and Outcome |  |  |  |  | No feeding outcomes reported |
| Halloran, D. R. and McClure, E. and Chakraborty, H. and Chomba, E. and Wright, L. L. and Carlo, W. A. | 2009 | Birth asphyxia survivors in a developing country | Journal of perinatology : official journal of the California Perinatal Association | 29 | 3 | 243-9 | No analysis of factors influencing HIE feeding outcomes |
| Hamrick, S. E. G. and Miller, S. P. and Newton, N. R. and Parer, J. T. and Ferriero, D. M. and Barkovich, A. J. and Partridge, J. C. | 2003 | Nucleated red blood cell counts: Not associated with brain injury or outcome | Pediatric Neurology | 29 | 4 | 278-283 | No feeding outcomes reported |
| Hang, Tran Thi Thanh | 2024 | Clinical and Experimental Implementation of Standardized Hypothermic Treatment for Neonatal Asphyxia in Low-Income Settings | Karolinska Institutet (Sweden) |  |  |  | No feeding outcomes reported |
| Hanrahan, J. D. and Cox, I. J. and Edwards, A. D. and Cowan, F. M. and Sargentoni, J. and Bell, J. D. and Bryant, D. J. and Rutherford, M. A. and Azzopardi, D. | 1998 | Persistent increases in cerebral lactate concentration after birth asphyxia | Pediatric Research | 44 | 3 | 304-311 | No feeding outcomes reported |
| Hansen, Gregory and Al Shafouri, Nasser and Narvey, Michael and Vallance, Jeff K. and Srinivasan, Ganesh | 2016 | High blood carbon dioxide variability and adverse outcomes in neonatal hypoxic ischemic encephalopathy | The journal of maternal-fetal & neonatal medicine : the official journal of the European Association of Perinatal Medicine, the Federation of Asia and Oceania Perinatal Societies, the International Society of Perinatal Obstetricians | 29 | 4 | 680-3 | No feeding outcomes reported |
| Harding, Celia and Bell, Nicoll and Griffiths, Sara and Michou, Emilia | 2023 | A descriptive evaluation of early feeding development of infants in a local neonatal unit | Journal of Neonatal Nursing | 29 | 4 | 681-686 | Mixed cohort |
| Hayakawa, M. and Ito, Y. and Saito, S. and Mitsuda, N. and Hosono, S. and Yoda, H. and Cho, K. and Otsuki, K. and Ibara, S. and Terui, K. and Masumoto, K. and Murakoshi, T. and Nakai, A. and Tanaka, M. and Nakamura, T. | 2014 | Incidence and prediction of outcome in hypoxic-ischemic encephalopathy in Japan | Pediatrics International | 56 | 2 | 215-221 | No feeding outcomes reported |
| Hayes, B. C. and Doherty, E. and Grehan, A. and Madigan, C. and McGarvey, C. and Mulavany, S. and Geary, M. and Matthews, T. G. and King, M. D. | 2012 | Are serum markers of liver and muscle injury useful in neonatal hypoxic-ischemic encephalopathy? | Journal of Neonatal-Perinatal Medicine | 5 | 4 | 305-310 | No feeding outcomes reported |
| Hayman, Michael and van Wezel-Meijler, Gerda and van Straaten, Henrica and Brilstra, Eva and Groenendaal, Floris and de Vries, Linda S. | 2019 | Punctate white-matter lesions in the full-term newborn: Underlying aetiology and outcome | European journal of paediatric neurology : EJPN : official journal of the European Paediatric Neurology Society | 23 | 2 | 280-287 | No feeding outcomes reported |
| Heston, Aaron and Patel, Anjali and Ahn, Kevin and Wresch, Delaney and Ridgway, Lauren and Shipman, Bethany and Graf, Katrina and Elkhwad, Mohammed and Muniraman, Hemananda | 2025 | Evaluation of Swallowing Dysfunction With Flexible Endoscopic Evaluation of Swallowing in the Neonatal Unit | American Journal of Speech-Language Pathology | 34 | 2 | 845-852 | Population not specified |
| Hill, Crystal D. and Jadcherla, Sudarshan R. | 2013 | Esophageal mechanosensitive mechanisms are impaired in neonates with hypoxic-ischemic encephalopathy | The Journal of pediatrics | 162 | 5 | 976-82 | No analysis of breastfeeding or lactation outcomes |
| Hossain, M. M. and Mannan, M. A. and Yeasmin, F. and Shaha, C. K. and Rahman, M. H. and Shahidullah, M. | 2013 | Short-term outcome of magnesium sulfate infusion in perinatal asphyxia | Mymensingh medical journal : MMJ | 22 | 4 | 727-35 | No analysis of breastfeeding or lactation outcomes |
| Huang, Hui-Zhi and Hu, Xiao-Feng and Wen, Xiao-Hong and Yang, Li-Qi | 2022 | Serum neuron-specific enolase, magnetic resonance imaging, and electrophysiology for predicting neurodevelopmental outcomes of neonates with hypoxic-ischemic encephalopathy: a prospective study | BMC pediatrics | 22 | 1 | 290 | No feeding outcomes reported |
| Hunt, Rod W. and Liley, Helen G. and Wagh, Deepika and Schembri, Rachel and Lee, Katherine J. and Shearman, Andrew D. and Francis-Pester, Samantha and deWaal, Koert and Cheong, Jeanie Y. L. and Olischar, Monika and Badawi, Nadia and Wong, Flora Y. and Osborn, David A. and Rajadurai, Victor Samuel and Dargaville, Peter A. and Headley, Bevan and Wright, Ian and Colditz, Paul B. and Newborn Electrographic Seizure Trial, Investigators | 2021 | Effect of Treatment of Clinical Seizures vs Electrographic Seizures in Full-Term and Near-Term Neonates: A Randomized Clinical Trial | JAMA network open | 4 | 12 | e2139604 | Mixed cohort |
| Ichiba, H. and Yokoi, T. and Tamai, H. and Ueda, T. and Kim, T. J. and Yamano, T. | 2006 | Neurodevelopmental outcome of infants with birth asphyxia treated with magnesium sulfate | Pediatrics International | 48 | 1 | 70-75 | No feeding outcomes reported |
| Ichiba, Hiroyuki and Tamai, Hiroshi and Negishi, Hirokuni and Ueda, Toru and Kim, Tae-Jang and Sumida, Yutaka and Takahashi, Yukihiro and Fujinaga, Hideshi and Minami, Hirotaka and Kansai Magnesium Study, Group | 2002 | Randomized controlled trial of magnesium sulfate infusion for severe birth asphyxia | Pediatrics international : official journal of the Japan Pediatric Society | 44 | 5 | 505-9 | No analysis of breastfeeding or lactation outcomes |
| Ikeda, N. and Awata, K. and Murano, Y. and Ohkawa, N. and Shoji, H. and Kantake, M. and Shimizu, T. | 2019 | Effects of probiotics on neonates with hypoxic-ischaemic encephalopathy after therapeutic hypothermia | Journal of Perinatal Medicine | 47 |  | eA297 | Conference abstract |
| Indrio, Flavia and Marchese, Flavia and Rinaldi, Matteo and Maffei, Gianfranco and Dargenio, Vanessa and Cinquepalmi, Roberta and Mantovani, Massimo Pettoello and Aceti, Arianna | 2022 | Is acidemia at birth a risk factor for functional gastrointestinal disorders? | European journal of pediatrics | 181 | 10 | 3625-3633 | Not definitive HIE diagnosis |
| Iqbal, Nadeem and Younus, Javaria and Malik, Muneeba and Fatima, Bushra and Imran, Ahmed and Maqbool, Shazia and Irfan Waheed, Khawaja Ahmad and Haque, Khalid | 2021 | The Neuroprotective Efficacy of Postnatal Magnesium Sulfate in Term or Near-Term Infants With Moderate-to-Severe Birth Asphyxia | Cureus | 13 | 8 | e16826 | No analysis of breastfeeding or lactation outcomes |
| Isweisi, Eman and Moore, Carmel Maria and Hurley, Tim and Sola-Visner, Martha and McCallion, Naomi and Ainle, Fionnuala Ni and Zareen, Zunera and Sweetman, Deirdre U. and Curley, Anna E. and Molloy, Eleanor J. and Newborn Brain Society, Guidelines and Publications, Committee | 2021 | Haematological issues in neonates with neonatal encephalopathy treated with hypothermia | Seminars in fetal & neonatal medicine | 26 | 4 | 101270 | Review paper |
| Jadas, V. and Brasseur-Daudruy, M. and Chollat, C. and Pellerin, L. and Devaux, A. M. and Marret, S. | 2014 | The contribution of the clinical examination, electroencephalogram, and brain MRI in assessing the prognosis in term newborns with neonatal encephalopathy. A cohort of 30 newborns before the introduction of treatment with hypothermia | Archives de Pediatrie | 21 | 2 | 125-133 | No feeding outcomes reported |
| Jadcherla, S. R. and Khot, T. and Moore, R. and Malkar, M. and Gulati, I. K. and Slaughter, J. L. | 2017 | Feeding Methods at Discharge Predict Long-Term Feeding and Neurodevelopmental Outcomes in Preterm Infants Referred for Gastrostomy Evaluation | Journal of Pediatrics | 181 |  | 125-130 | Mixed cohort |
| Javed, R. and Hodson, J. and Gowda, H. | 2024 | Prevalence of Pulmonary Hypertension During Therapeutic Hypothermia for Hypoxic Ischemic Encephalopathy and Evaluation of Short-Term Outcomes | Therapeutic Hypothermia and Temperature Management |  |  |  | No analysis of breastfeeding or lactation outcomes |
| Jayakumar, S. and Burton, V. J. and Perin, J. and Asafu-Adjaye, D. and Cristofalo, E. and Northington, F. and Valdez, R. C. and Leppert, M. L. and Allen, M. and Gerner, G. | 2022 | Predictors of Developmental Outcomesin Hypoxic-ischemic Encephalopathy Treated with Hypothermia | Pediatrics | 149 |  |  | Conference abstract |
| Jenkins, D. and Brennan, A. and Badran, B. and George, M. and Wiest, D. and S and ra, G. | 2023 | N-acetylcysteine (NAC) facilitates motor learning in infants of diabetic mothers (IDM) failing oral feeds treated with transcutaneous auricular vagus nerve stimulation (taVNS) | Brain Stimulation | 16 | 1 | 380-381 | Conference abstract |
| Jenkins, D. and George, M. and DeVries, W. and Moss, H. and Dancy, M. and Cook, D. and Mappin, G. and Badran, B. | 2019 | A novel pilot study of tavns paired with oral feeding in neonates with brain injury | Brain Stimulation | 12 | 2 | 520 | Conference abstract |
| Jenkins, Dorothea D. and Rollins, Laura Grace and Perkel, Jessica K. and Wagner, Carol L. and Katikaneni, Lakshmi P. and Bass, W. Thomas and Kaufman, David A. and Horgan, Michael J. and Languani, Sheela and Givelichian, Lawrence and Sankaran, Koravangattu and Yager, Jerome Y. and Martin, Renee H. | 2012 | Serum cytokines in a clinical trial of hypothermia for neonatal hypoxic-ischemic encephalopathy | Journal of cerebral blood flow and metabolism : official journal of the International Society of Cerebral Blood Flow and Metabolism | 32 | 10 | 1888-96 | No feeding outcomes reported |
| Jensen, P. S. and Shubert, T. and Sitaram, S. and Gulati, I. K. and Jadcherla, S. | 2015 | Pharyngeal stimulus induced reflexes are impaired in infants with hypoxic ischemic encephalopathy (HIE) during maturation | Gastroenterology | 148 | 4 | S896 | Conference abstract |
| Jensen, P. S. and Gulati, I. K. and Shubert, T. R. and Sitaram, S. and Sivalingam, M. and Hasenstab, K. A. and El-Mahdy, M. A. and Jadcherla, S. R. | 2017 | Pharyngeal stimulus-induced reflexes are impaired in infants with perinatal asphyxia: Does maturation modify? | Neurogastroenterology and motility : the official journal of the European Gastrointestinal Motility Society | 29 | 7 |  | No analysis of breastfeeding or lactation outcomes |
| Jhajra, S. and Nanda, D. and Dalal, J. S. | 2024 | Enteral feeding in neonates >=34 weeks of gestation with moderate to severe birth asphyxia: A retrospective observational study | Tropical Doctor | 54 | 4 | 312 EP - 316 | No feeding outcomes reported |
| Jiang, C. M. and Yang, Y. H. and Chen, L. Q. and Shuai, X. H. and Lu, H. and Xiang, J. H. and Liu, Z. L. and Zhu, Y. X. and Xu, R. Y. and Zhu, D. R. and Huang, X. M. | 2015 | Early amplitude-integrated EEG monitoring 6 h after birth predicts long-term neurodevelopment of asphyxiated late preterm infants | European Journal of Pediatrics | 174 | 8 | 1043-1052 | No feeding outcomes reported |
| Jiang, Z. D. and Liu, M. Y. and Shi, B. P. and Lin, L. and Bu, C. F. and Wilkinson, A. R. | 2008 | Brainstem auditory outcomes and correlation with neurodevelopment after perinatal asphyxia | Pediatric Neurology | 39 | 3 | 189-195 | No feeding outcomes reported |
| Joshi, S. and Cardona, V. Q. and Taylor, J. and Poletto, E. and Menkiti, O. | 2019 | Neuroimaging and neurodevelopmental outcomes in neonates with hypoxic ischemic encephalopathy (HIE) who received extracorporeal membrane oxygenation (ECMO) and controlled hypothermia (CH) | ASAIO Journal | 65 |  | 87 | Conference abstract |
| Julkunen, M. K. and Uotila, J. and Eriksson, K. and Janas, M. and Luukkaala, T. and Tammela, O. | 2012 | Obstetric parameters and Doppler findings in cerebral circulation as predictors of 1 year neurodevelopmental outcome in asphyxiated infants | Journal of perinatology : official journal of the California Perinatal Association | 32 | 8 | 631-8 | No feeding outcomes reported |
| Juul, S. and Gonzalez, F. and Van Meurs, K. and Ballard, R. and Wu, Y. | 2016 | A phase II randomized controlled trial of erythropoietin and hypothermia for neonatal neuroprotecion in hypoxic-ischemic encephalopathy | Journal of Investigative Medicine | 64 | 1 | 285 | Conference abstract |
| Kader, A. A. A. and El-Kholy, S. and El-Sebaie, D. and Mostafa, S. and El-Gohary, A. and Afifi, L. and Raafat, H. | 2009 | Predictive value of multimodality evoked potentials in asphyxiated term newborns | Egyptian Journal of Neurology, Psychiatry and Neurosurgery | 46 | 2 | 385-394 | No feeding outcomes reported |
| Kadri, M. and Shu, S. and Holshouser, B. and Deming, D. and Hopper, A. and Peverini, R. and Ashwal, S. | 2003 | Proton magnetic resonance spectroscopy improves outcome prediction in perinatal CNS insults | Journal of Perinatology | 23 | 3 | 181-185 | No feeding outcomes reported |
| Kali, Gugulabatembunamahlubi Tenjiwe Jabulile and Martinez-Biarge, Miriam and Van Zyl, Jeanetta and Smith, Johan and Rutherford, Mary | 2016 | Therapeutic hypothermia for neonatal hypoxic-ischaemic encephalopathy had favourable outcomes at a referral hospital in a middle-income country | Acta paediatrica (Oslo, Norway : 1992) | 105 | 7 | 806-15 | No analysis of factors influencing HIE feeding outcomes |
| Karlsson, Mathias and Wiberg-Itzel, Eva and Chakkarapani, Ela and Blennow, Mats and Winbladh, Birger and Thoresen, Marianne | 2010 | Lactate dehydrogenase predicts hypoxic ischaemic encephalopathy in newborn infants: a preliminary study | Acta paediatrica (Oslo, Norway : 1992) | 99 | 8 | 1139-44 | No feeding outcomes reported |
| Kelly, Rod and Ramaiah, S. M. and Sheridan, Helen and Cruickshank, Hilary and Rudnicka, Magda and Kissack, Chris and Becher, Julie-Clare and Stenson, Ben J. | 2018 | Dose-dependent relationship between acidosis at birth and likelihood of death or cerebral palsy | Archives of disease in childhood. Fetal and neonatal edition | 103 | 6 | F567-F572 | No feeding outcomes reported |
| Khashaba, M. T. and El-Ayouty, M. and El-Sayed, O. and Hasanen, B. M. and Aly, H. | 2004 | Interleukin-1 beta (IL-1 beta) and interleukin-6 (IL-6), but not tumor necrosis factor-alpha (TNF-alpha) correlate with neurodevelopmental outcomes (NDO) at six months in term infants with hypoxic-ischemic encephalopathy (HIE) | Pediatric Research | 55 | 4 | 58A-58A | Conference abstract |
| Khushdil, Arshad and Ahmed, Zeeshan and Ehsan, Azra | 2021 | Role of Citicoline in Treatment of Moderate to Severe Birth Asphyxia: A Pilot Project | Journal of the College of Physicians and Surgeons--Pakistan : JCPSP | 31 | 12 | 1511-1512 | No analysis of factors influencing HIE feeding outcomes |
| Kim, Sae Yun and Kang, Hyun-Mi and Im, Soo-Ah and Youn, Young-Ah | 2025 | The impact of clinical seizures and adverse brain MRI patterns in neonates with hypoxic-ischemic encephalopathy and abnormal neurodevelopment | Clinics (Sao Paulo, Brazil) | 80 |  | 100533 | No feeding outcomes reported |
| Kimkool, P. and Ollerenshaw, R. H. and Duckworth, E. C. and King, K. and Beardsall, K. | 2014 | How do we and how should we optimise nutrition during therapeutic hypothermia? | Archives of Disease in Childhood: Fetal and Neonatal Edition | 99 |  | A62-A64 | Conference abstract |
| Kirkley, M. and Kaseman, S. and Gralla, J. and Melara, D. and Grover, T. and Delaney, C. | 2016 | Short-term outcomes of neonates with mild hypoxic-ischemic encephalopathy after therapeutic hypothermia | Journal of Investigative Medicine | 64 | 1 | 338 | Conference abstract |
| Kitai, Y. and Arai, H. and Hirai, S. | 2010 | Profound cerebral dysfunction and brainstem disorder underlie dysphagia in dyskinetic cerebral palsy from asphyxia | European Journal of Paediatric Neurology | 14 | 6 | 553 | Conference abstract |
| Kitai, Y. and Arai, H. and Hirai, S. | 2012 | Bulbar dysfunction after hypothermia for neonatal hypoxic-ischemic encephalopathy | Developmental Medicine and Child Neurology | 54 |  | 114 | Conference abstract |
| Kitai, Y. and Hirai, S. and Ogura, K. and Okuyama, N. and Hirotsune, M. and Mizutani, S. and Arai, H. | 2019 | Etiology of dyskinetic cerebral palsy in Japan and its relationship to functional outcomes | Developmental Medicine and Child Neurology | 61 |  | 07-Aug | Conference abstract |
| Kitai, Y. and Hirai, S. and Okuyama, N. and Hirotsune, M. and Nishimoto, S. and Hirano, S. and Arai, H. | 2021 | Functional outcomes of children with dyskinetic cerebral palsy depend on etiology and gestational age | European Journal of Paediatric Neurology | 30 |  | 108-112 | No analysis of factors influencing HIE feeding outcomes |
| Kitai, Y. and Ohmura, K. and Hirai, S. and Arai, H. | 2015 | Long-term outcome of childhood hypoxic-ischemic encephalopathy | No To Hattatsu | 47 | 1 | 43-48 | Not neonatal HIE |
| Kobayashi, A. and Wada, M. and Usuda, T. | 2015 | Internal cerebral vein flow and neurological outcome in asphyxiated newborns who received hypothermia therapy | Journal of Perinatal Medicine | 43 |  |  | Conference abstract |
| Kolsi, N. and Chaabene, M. and Rregaieg, C. and Bouraoui, A. and Charfi, M. and Ben Hamad, A. and Regaieg, R. and Hmida, N. and Ben Thabet, A. and Gargouri, A. | 2022 | EARLY ONSET NEONATAL HYPOCALCEMIA | Pediatric Critical Care Medicine | 23 | 11 |  | Conference abstract |
| Konar, Mithun Ch and ra and Islam, Kamirul and Sil, Archan and Nayek, Kaustav and Barik, Kanailal | 2021 | Effect of Music on Outcomes of Birth Asphyxia: A Randomized Controlled Trial | Journal of tropical pediatrics | 67 | 2 |  | No feeding outcomes reported |
| Koshy, Beena and Padankatti, Caroline Sanjeev and George, Koshy C. and Thomas, Niranjan | 2011 | Neurodevelopmental outcome following whole body cooling for perinatal asphyxia | Indian pediatrics | 48 | 12 | 982-3 | No feeding outcomes reported |
| Kota, S. and Liu, Y. L. and Bitar, L. and Chalak, L. | 2024 | EEG Spectral Power and Neurovascular Coupling as Early Predictors of Neurodevelopmental Outcome in Neonatal Hypoxic-Ischemic Encephalopathy | Annual International Conference of the IEEE Engineering in Medicine and Biology Society. IEEE Engineering in Medicine and Biology Society. Annual International Conference | 2024 | 1 | 1 EP - 6 | No feeding outcomes reported |
| Kruger, Esedra and Kritzinger, Alta and Pottas, Lidia | 2019 | Oropharyngeal Dysphagia in Breastfeeding Neonates with Hypoxic-Ischemic Encephalopathy on Therapeutic Hypothermia | Breastfeeding medicine : the official journal of the Academy of Breastfeeding Medicine | 14 | 10 | 718-723 | No analysis of factors influencing HIE feeding outcomes |
| Ku, J. K. and Heo, Y. J. and Lee, K. S. and Lee, B. L. | 2018 | Clinical Findings and Neurologic Outcome in Neonatal Encephalopathy With White Matter Injury Accompanied by Rotavirus | Journal of Child Neurology | 33 | 4 | 297-305 | No feeding outcomes reported |
| Kuenzle, Ch and Baenziger, O. and Martin, E. and Thun-Hohenstein, L. and Steinlin, M. and Good, M. and Fanconi, S. and Boltshauser, E. and Largo, R. H. | 1994 | Prognostic value of early MR imaging in term infants with severe perinatal asphyxia | Neuropediatrics | 25 | 4 | 191-200 | No feeding outcomes reported |
| Kulkarni, T. and Charki, S. and Biradar, V. and Tyagaraj, T. and Anju, T. and Patil, M. M. and Kalyanshettar, S. S. and Patil, S. V. | 2021 | Feasibility of minimal enteral nutrition in neonates with perinatal asphyxia during therapeutic hypothermia: A randomized controlled trial | Current Pediatric Research | 25 | 3 | 466-471 | No analysis of breastfeeding or lactation outcomes |
| Kumar, A. Senthil and Ch and rasekaran, Aparna and Asokan, Rajamannar and Gopinathan, Kathirvelu | 2016 | Prognostic Value of Resistive Index in Neonates with Hypoxic Ischemic Encephalopathy | Indian pediatrics | 53 | 12 | 1079-1082 | No feeding outcomes reported |
| Kumar, Praveen | 2006 | A Study of the Importance of Neurosonogram in Long Term follow up of Birth Asphyxia Cases in New Born |  |  |  |  | Not able to access abstract or full text |
| Kwon, Jennifer M. and Guillet, Ronnie and Shankaran, Seetha and Laptook, Abbot R. and McDonald, Scott A. and Ehrenkranz, Richard A. and Tyson, Jon E. and O'Shea, T. Michael and Goldberg, Ronald N. and Donovan, Edward F. and Fanaroff, Avroy A. and Poole, W. Kenneth and Higgins, Rosemary D. and Walsh, Michele C. and Eunice Kennedy Shriver National Institute of Child, Health and Human Development Neonatal Research, Network | 2011 | Clinical seizures in neonatal hypoxic-ischemic encephalopathy have no independent impact on neurodevelopmental outcome: secondary analyses of data from the neonatal research network hypothermia trial | Journal of child neurology | 26 | 3 | 322-8 | No feeding outcomes reported |
| Lakhkar, B. B. and Damake, S. and Meshram, R. and Lohia, S. and Karotkar, S. | 2020 | Therapeutic hypothermia in moderate and severe hypoxic-ischemic encephalopathy: Use of low-cost vs high technology cooling technique | International Journal of Current Research and Review | 12 | 22 | 31-34 | Protocol or trial registry entry |
| Lally, Peter J. and Montaldo, Paolo and Oliveira, Vania and Soe, Aung and Swamy, Ravi and Bassett, Paul and Mendoza, Josephine and Atreja, Gaurav and Kariholu, Ujwal and Pattnayak, Santosh and Sashikumar, Palaniappan and Harizaj, Helen and Mitchell, Martin and Ganesh, Vijayakumar and Harigopal, Sundeep and Dixon, Jennifer and English, Philip and Clarke, Paul and Muthukumar, Priya and Satodia, Prakash and Wayte, Sarah and Abernethy, Laurence J. and Yajamanyam, Kiran and Bainbridge, Alan and Price, David and Huertas, Angela and Sharp, David J. and Kalra, Vaneet and Chawla, Sanjay and Shankaran, Seetha and Thayyil, Sudhin and consortium, Marble | 2019 | Magnetic resonance spectroscopy assessment of brain injury after moderate hypothermia in neonatal encephalopathy: a prospective multicentre cohort study | The Lancet. Neurology | 18 | 1 | 35-45 | No feeding outcomes reported |
| Lally, Peter J. and Montaldo, Paolo and Oliveira, Vania and Swamy, Ravi Shankar and Soe, Aung and Shankaran, Seetha and Thayyil, Sudhin | 2018 | Residual brain injury after early discontinuation of cooling therapy in mild neonatal encephalopathy | Archives of disease in childhood. Fetal and neonatal edition | 103 | 4 | F383-F387 | No feeding outcomes reported |
| Landau, Yuval and Berger, Irit and Marom, Ronela and M and el, Dror and Ben Sira, Liat and Fattal-Valevski, Aviva and Peylan, Tali and Levi, Loren and Dolberg, Shaul and Bassan, Haim | 2011 | Therapeutic hypothermia for asphyxiated newborns: experience of an Israeli tertiary center | The Israel Medical Association journal : IMAJ | 13 | 1 | 29-33 | No analysis of factors influencing HIE feeding outcomes |
| Langeslag, J. F. and Groenendaal, F. and Roosendaal, S. D. and De Vries, L. S. and Onl and , W. and Leeflang, M. M. G. and Groot, P. F. C. and Van Kaam, A. H. and De Haan, T. R. | 2022 | Outcome Prediction and Inter-Rater Comparison of Four Brain Magnetic Resonance Imaging Scoring Systems of Infants with Perinatal Asphyxia and Therapeutic Hypothermia | Neonatology | 119 | 3 | 311-319 | No feeding outcomes reported |
| Laptook, Abbot R. and Shankaran, Seetha and Barnes, Patrick and Rollins, Nancy and Do, Barbara T. and Parikh, Nehal A. and Hamrick, Shannon and Hintz, Susan R. and Tyson, Jon E. and Bell, Edward F. and Ambalavanan, Namasivayam and Goldberg, Ronald N. and Pappas, Athina and Huitema, Carolyn and Pedroza, Claudia and Chaudhary, Aasma S. and Hensman, Angelita M. and Das, Abhik and Wyckoff, Myra and Khan, Amir | 2021 | Limitations of Conventional Magnetic Resonance Imaging as a Predictor of Death or Disability Following Neonatal Hypoxic-Ischemic Encephalopathy in the Late Hypothermia Trial | Journal of Pediatrics | 230 |  | 106-106 | No feeding outcomes reported |
| Laval, N. and Paquette, M. and Talsmat, H. and Mar and yuk, B. and Wintermark, P. and Birca, A. and Pinchefsky, E. F. and Tremblay, S. | 2023 | Exposure to Maternal Diabetes during Pregnancy Is Associated with Aggravated Short- Term Neonatal and Neurological Outcomes following Perinatal Hypoxic-Ischemic Encephalopathy | American Journal of Perinatology |  |  | 10 | No analysis of breastfeeding or lactation outcomes |
| Lee, Inn-Chi and Hong, Syuan-Yu and Weng, Yi-Ho and Chen, Yi-Ting | 2021 | Amplitude Integrated Electroencephalography and Continuous Electroencephalography Monitoring Is Crucial in High-Risk Infants and Their Findings Correlate With Neurodevelopmental Outcomes | Frontiers in pediatrics | 9 |  | 691764 | No feeding outcomes reported |
| Lee, I. C. and Yang, J. J. and Liou, Y. M. | 2022 | Early blood glucose level post-admission correlates with the outcomes and oxidative stress in neonatal hypoxic-ischemic encephalopathy | Antioxidants | 11 | 1 |  | No feeding outcomes reported |
| Lee, I. C. and Yu, C. S. and Hu, Y. C. and Wang, X. A. | 2023 | Unconjugated bilirubin is correlated with the severeness and neurodevelopmental outcomes in neonatal hypoxic-ischemic encephalopathy | Scientific Reports | 13 | 1 | 10 | No feeding outcomes recorded |
| Lemmers, P. M. A. and Zwanenburg, R. J. and Benders, Mjnl and de Vries, L. S. and Groenendaal, F. and van Bel, F. and Toet, M. C. | 2013 | Cerebral oxygenation and brain activity after perinatal asphyxia: does hypothermia change their prognostic value? | Pediatric Research | 74 | 2 | 180-185 | No feeding outcomes reported |
| Leon, Rachel L. and Krause, Katherine E. and Sides, Rebecca S. and Koch, Mary Beth and Trautman, Michael S. and Mietzsch, Ulrike | 2022 | Therapeutic Hypothermia in Transport Permits Earlier Treatment Regardless of Transfer Distance | American journal of perinatology | 39 | 6 | 633-639 | No analysis of breastfeeding or lactation outcomes |
| Leon-Lozano, M. Z. and Arnaez, J. and Valls, A. and Arca, G. and Agut, T. and Alarcon, A. and Garcia-Alix, A. | 2020 | Cerebrospinal fluid levels of neuron-specific enolase predict the severity of brain damage in newborns with neonatal hypoxic-ischemic encephalopathy treated with hypothermia | PLoS ONE | 15 | 6 | e0234082 | No feeding outcomes reported |
| Liao, H. T. | 1997 | Anterior cerebral artery Doppler ultrasonography for prediction of outcome after perinatal asphyxia | Acta Paediatrica Sinica | 38 | 3 | 208-212 | No feeding outcomes reported |
| Lin, H. C. and Shue, T. C. | 2008 | Prediction of neurodevelopmental outcome after perinatal asphyxia via transcranial cerebral artery Doppler ultrasonography | Pediatrics | 121 |  | S147-S147 | Conference abstract |
| Lin, Y. K. and Hwang-Bo, S. and Seo, Y. M. and Youn, Y. A. | 2021 | Clinical seizures and unfavorable brain MRI patterns in neonates with hypoxic ischemic encephalopathy | Medicine | 100 | 12 | e25118 | No response from author when attempt to clarify details for inclusion |
| Lingwood, B. E. and Healy, G. N. and Kecskes, Z. and Dunster, K. R. and Gray, P. H. and Ward, L. C. and Colditz, P. B. | 2009 | Prediction of outcome following hypoxia/ischaemia in the human infant using cerebral impedance | Clinical neurophysiology : official journal of the International Federation of Clinical Neurophysiology | 120 | 2 | 225-30 | No feeding outcomes reported |
| Lipp-Zwahlen, A. E. and Deonna, T. and Chrzanowski, R. and Micheli, J. L. and Calame, A. | 1985 | Temporal evolution of hypoxic-ischaemic brain lesions in asphyxiated full-term newborns as assessed by computerized tomography | Neuroradiology | 27 | 2 | 138-44 | No analysis of factors influencing HIE feeding outcomes |
| Lipp-Zwahlen, A. E. and Deonna, T. and Micheli, J. L. | 1985 | Prognostic value of neonatal CT scans in asphyxiated term babies: Low density score compared with neonatal neurological signs | Neuropediatrics | 16 | 4 | 209-217 | No analysis of factors influencing HIE feeding outcomes |
| Logitharajah, Pavithra and Rutherford, Mary A. and Cowan, Frances M. | 2009 | Hypoxic-ischemic encephalopathy in preterm infants: antecedent factors, brain imaging, and outcome | Pediatric research | 66 | 2 | 222-9 | No feeding outcomes reported |
| Lucke, A. M. and Kaiser, J. and Gollins, L. and Hagan, J. and Hair, A. | 2019 | Introduction and advancement of enteral nutrition after therapeutic hypothermia in neonates with hypoxic-ischemic encephalopathy | Pediatrics | 144 | 2 |  | Conference abstract |
| Lugli, Licia and Guidotti, Isotta and Pugliese, Marisa and Roversi, Maria Federica and Bedetti, Luca and Della Casa Muttini, Elisa and Cavalleri, Francesca and Todeschini, Aless and ra and Genovese, Maurilio and Ori, Luca and Amato, Maria and Miselli, Francesca and Lucaccioni, Laura and Bertoncelli, Natascia and C and ia, Francesco and Maura, Tommaso and Iughetti, Lorenzo and Ferrari, Fabrizio and Berardi, Alberto | 2022 | Polygraphic EEG Can Identify Asphyxiated Infants for Therapeutic Hypothermia and Predict Neurodevelopmental Outcomes | Children (Basel, Switzerland) | 9 | 8 |  | No feeding outcomes reported |
| Lv, Hong-Yan and Wu, Su-Jing and Wang, Qiu-Li and Yang, Li-Hong and Ren, Peng-Shun and Qiao, Bao-Jun and Wang, Zhi-Ying and Li, Jia-Hong and Gu, Xiu-Ling and Li, Lian-Xiang | 2017 | Effect of erythropoietin combined with hypothermia on serum tau protein levels and neurodevelopmental outcome in neonates with hypoxic-ischemic encephalopathy | Neural regeneration research | 12 | 10 | 1655-1663 | No feeding outcomes reported |
| Maddock, M. A. and Kecskes, Z. and Lindsay, N. and Lingwood, B. | 2010 | Predicting neonatal and infant morbidity after perinatal hypoxic-ischaemic injury | Journal of Paediatrics and Child Health | 46 |  | 73 | Conference abstract |
| Magalhaes, M. and Rodrigues, F. P. M. and Chopard, M. R. T. and De Melo, V. C. A. and Melhado, A. and Oliveira, I. and Gallacci, C. B. and Pachi, P. R. and Lima Neto, T. B. | 2015 | Neuroprotective body hypothermia among newborns with hypoxic ischemic encephalopathy: Three-year experience in a tertiary university hospital. a retrospective observational study | Sao Paulo Medical Journal | 133 | 4 | 314-319 | No analysis of factors influencing HIE feeding outcomes |
| Majnemer, A. and Rosenblatt, B. and Riley, P. S. | 1990 | Prognostic significance of multimodality evoked response testing in high-risk newborns | Pediatric Neurology | 6 | 6 | 367-374 | No analysis of factors influencing HIE feeding outcomes |
| Malan, R. and Van Der Linde, J. and Kritzinger, A. and Graham, M. A. and Kruger, E. and Kollapen, K. and Lockhat, Z. | 2022 | Evolution of swallowing and feeding abilities of neonates with hypoxic-ischaemic encephalopathy during hospitalisation: A case series | International Journal of Speech-Language Pathology |  |  | 45566 | No analysis of breastfeeding or lactation outcomes |
| Malan, R. and van der Linde, J. and Kritzinger, A. and Graham, M. A. and Krueger, E. | 2023 | Evolution of Feeding and Developmental Outcomes in Infants With Moderate Hypoxic-Ischemic Encephalopathy: A Pilot Study | Neonatal Network | 42 | 5 | 264-275 | No analysis of factors influencing HIE feeding outcomes |
| Malik, G. K. and Trivedi, R. and Gupta, R. K. and Hasan, K. M. and Hasan, M. and Gupta, A. and P and ey, C. M. and Narayana, P. A. | 2006 | Serial quantitative diffusion tensor MRI of the term neonates with hypoxic-ischemic encephalopathy (HIE) | Neuropediatrics | 37 | 6 | 337-343 | No feeding outcomes reported |
| Malvia, S. and Jain, P. and Bhardwaj, B. K. | 2022 | The Relationship between Umbilical Cord Arterial PH and Short Term Outcome in Neonates | International Journal of Pharmaceutical and Clinical Research | 14 | 8 | 168-172 | Mixed cohort |
| Martin, E. and Buchli, R. and Ritter, S. and Schmid, R. and Largo, R. H. and Boltshauser, E. and Fanconi, S. and Duc, G. and Rumpel, H. | 1996 | Diagnostic and prognostic value of cerebral 31P magnetic resonance spectroscopy in neonates with perinatal asphyxia | Pediatric Research | 40 | 5 | 749-758 | No analysis of factors influencing HIE feeding outcomes |
| Martinez-Biarge, M. and Bregant, T. and Wusthoff, C. J. and Chew, A. T. and Diez-Sebastian, J. and Rutherford, M. A. and Cowan, F. M. and Martinez-Biarge, Miriam and Bregant, Tina and Wusthoff, Courtney J. and Chew, Andrew T. M. and Diez-Sebastian, Jesus and Rutherford, Mary A. and Cowan, Frances M. | 2012 | White matter and cortical injury in hypoxic-ischemic encephalopathy: antecedent factors and 2-year outcome | Journal of Pediatrics | 161 | 5 | 799-807 | No analysis of breastfeeding or lactation outcomes |
| Martinez-Biarge, Miriam and Diez-Sebastian, Jesus and Wusthoff, Courtney J. and Lawrence, Stacey and Aloysius, Annie and Rutherford, Mary A. and Cowan, Frances M. | 2012 | Feeding and communication impairments in infants with central grey matter lesions following perinatal hypoxic-ischaemic injury | European journal of paediatric neurology : EJPN : official journal of the European Paediatric Neurology Society | 16 | 6 | 688-96 | No analysis of breastfeeding or lactation outcomes |
| Martinez-Biarge, M. and Diez-Sebastian, J. and Lawrence, S. and Aloysius, A. and Rutherford, M. A. and Cowan, F. M. | 2010 | PREDICTING FEEDING PROBLEMS IN INFANTS WITH CENTRAL GREY MATTER DAMAGE FOLLOWING HYPOXIC-ISCHAEMIC ENCEPHALOPATHY (HIE) | Pediatric Research | 68 |  | 89-89 | Conference abstract |
| Martinez-Biarge, M. and Diez-Sebastian, J. and Rutherford, M. A. and Cowan, F. M. | 2010 | Outcomes after central grey matter injury in term perinatal hypoxic-ischaemic encephalopathy | Early Human Development | 86 | 11 | 675-682 | Duplicate of data already included |
| Martinovski, H. and Khanal, L. and Kraft, D. and Natarajan, G. | 2025 | Enteral Feeding in Neonatal Hypoxic-Ischemic Encephalopathy | American Journal of Perinatology |  |  | 8 | No analysis of factors influencing HIE feeding outcomes |
| Massaro, An N. and Wu, Yvonne W. and Bammler, Theo K. and Comstock, Bryan and Mathur, Amit and McKinstry, Robert C. and Chang, Taeun and Mayock, Dennis E. and Mulkey, Sarah B. and Van Meurs, Krisa and Juul, S and ra | 2018 | Plasma Biomarkers of Brain Injury in Neonatal Hypoxic-Ischemic Encephalopathy | The Journal of pediatrics | 194 |  | 67-75.e1 | No feeding outcomes reported |
| Mauro, I. and Franz, A. and Baraldi, E. and Carnielli, V. and Paterlini, G. and Napolitano, M. and Faldini, P. F. and Barbarini, M. and Lista, G. and Visintin, G. and Mastretta, E. and Vento, G. and Fumagalli, M. and Binotti, M. and Cattarossi, L. | 2018 | The albino trail effect of allopurinol in addition to hypothermia for hypoxic-ischemic brain injury on neurocognitive outcome | American Journal of Perinatology | 35 |  |  | Conference abstract |
| Mbatha, S. and Nakwa, F. L. and Th and rayen, K. and Velaphi, S. | 2021 | Neurodevelopmental outcome in neonates with hypoxic-ischaemic encephalopathy managed with therapeutic hypothermia in a tertiary-level public hospital outside an intensive care unit setting | Paediatrics and international child health | 41 | 3 | 171-176 | No feeding outcomes reported |
| McBride, M. C. and Laroia, N. and Guillet, R. | 2000 | Electrographic seizures in neonates correlate with poor neurodevelopmental outcome | Neurology | 55 | 4 | 506-513 | No analysis of factors influencing HIE feeding outcomes |
| McGinn, E. A. and Powers, A. and Galas, M. and Lyden, E. and Peeples, E. S. | 2020 | Neonatal Vitamin D Status Is Associated with the Severity of Brain Injury in Neonatal Hypoxic-Ischemic Encephalopathy: A Pilot Study | Neuropediatrics | 51 | 4 | 251-258 | No analysis of breastfeeding or lactation outcomes |
| McGowan, Meaghan M. and O'Kane, Alex and ra C. and Vezina, Gilbert and Chang, Taeun and Bendush, Nicole and Glass, Penny and Gai, Jiaxiang and Bost, James and Everett, Allen D. and Massaro, An N. | 2021 | Serial plasma biomarkers of brain injury in infants with neonatal encephalopathy treated with therapeutic hypothermia | Pediatric research | 90 | 6 | 1228-1234 | No feeding outcomes reported |
| Mejaski-Bosnjak, V. and Besenski, N. and Lujic, L. and Polak, J. and Buljan-Fl and er, G. and Marusic-Della Marina, B. and Vukadin, M. S. | 1992 | Hypoxic-ischemic brain damage in term neonates--the relation of neurodevelopmental handicap to cranial ultrasound findings | Neurologia croatica : glasilo Udruzenja neurologa Jugoslavije = official journal of Yugoslav Neurological Association | 41 | 3 | 117-29 | No feeding outcomes reported |
| Mejaskibosnjak, V. and Besenski, N. and Duranovic, V. and Polakbabic, J. and Duplancic, R. and Lujic, L. and Marusicdellamarina, B. | 1994 | CEREBRAL-PALSY IN CHILDREN WITH SUBCORTICAL LEUKOMALACIA | Neurologia Croatica | 43 | 4 | 221-231 | No feeding outcomes reported |
| Mellis, C. | 2020 | Therapeutic hypothermia for mild hypoxic-ischaemic encephalopathy | Journal of Paediatrics & Child Health | 56 | 2 | 346-346 | Commentary paper |
| Mercuri, E. and Haataja, L. and Guzzetta, A. and Anker, S. and Cowan, F. and Rutherford, M. and Andrew, R. and Braddick, O. and Cioni, G. and Dubowitz, L. and Atkinson, J. | 1999 | Visual function in term infants with hypoxic-ischaemic insults: Correlation with neurodevelopment at 2 years of age | Archives of Disease in Childhood: Fetal and Neonatal Edition | 80 | 2 | F99-F104 | No feeding outcomes reported |
| Mercuri, E. and Ricci, D. and Cowan, F. M. and Lessing, D. and Frisone, M. F. and Haataja, L. and Counsell, S. J. and Dubowitz, L. M. and Rutherford, M. A. | 2000 | Head growth in infants with hypoxic-ischemic encephalopathy: Correlation with neonatal magnetic resonance imaging | Pediatrics | 106 | 2 | 235-243 | No feeding outcomes reported |
| Mietzsch, U. and Flibotte, J. J. and Law, J. B. and Puia-Dumitrescu, M. and Juul, S. E. and Wood, T. R. | 2023 | Temperature dysregulation during therapeutic hypothermia predicts long-term outcome in neonates with HIE | Journal of Cerebral Blood Flow and Metabolism | 43 | 7 | 1180-1193 | No feeding outcomes reported |
| Miller, S. P. and Ramaswamy, V. and Michelson, D. and Barkovich, A. J. and Holshouser, B. and Wycliffe, N. and Glidden, D. V. and Deming, D. and Partridge, J. C. and Wu, Y. W. and Ashwal, S. and Ferriero, D. M. | 2005 | Patterns of brain injury in term neonatal encephalopathy | Journal of Pediatrics | 146 | 4 | 453-460 | No feeding outcomes reported |
| Misra, P. K. and Srivastava, N. and Malik, G. K. and Kapoor, R. K. and Srivastava, K. L. and Rastogi, S. | 1994 | Outcome in relation to Apgar score in term neonates | Indian pediatrics | 31 | 10 | 1215-8 | No feeding outcomes reported |
| Mitra, Subhabrata and Bale, Gemma and Highton, David and Gunny, Roxanna and Uria-Avellanal, Cristina and Bainbridge, Alan and Sokolska, Magdalena and Price, David and Huertas-Ceballos, Angela and Kendall, Giles S. and Meek, Judith and Tachtsidis, Ilias and Robertson, Nicola J. | 2019 | Pressure passivity of cerebral mitochondrial metabolism is associated with poor outcome following perinatal hypoxic ischemic brain injury | Journal of cerebral blood flow and metabolism : official journal of the International Society of Cerebral Blood Flow and Metabolism | 39 | 1 | 118-130 | No feeding outcomes reported |
| Moen, A. and Hamrick, S. E. and Sewell, E. and Patel, R. M. and Jernigan, S. | 2021 | Prevalence of acute kidney and hepatic injury in infants with hypoxic-ischemic encephalopathy and association with adverse short-term outcomes | Journal of Investigative Medicine | 69 | 2 | 648 | Conference abstract |
| Mohammed Rusli, E. R. and Ismail, J. and Wei, W. S. and Ishak, S. and Jaafar, R. and Zaki, F. M. | 2019 | Neonatal hypoxic encephalopathy: Correlation between post-cooling brain MRI findings and 2 years neurodevelopmental outcome | Indian Journal of Radiology and Imaging | 29 | 4 | 350-355 | No feeding outcomes reported |
| Mondal, N. and Bhat, B. V. and Banupriya, C. and Koner, B. C. and Mondal, Nivedita and Bhat, B. Vishnu and Banupriya, C. and Koner, Bidhan C. | 2010 | Oxidative stress in perinatal asphyxia in relation to outcome | Indian Journal of Pediatrics | 77 | 5 | 515-517 | No feeding outcomes reported |
| Montaldo, P. and Caredda, E. and Pugliese, U. and Zanfardino, A. and Delehaye, C. and Inserra, E. and Capozzi, L. and Chello, G. and Capristo, C. and Del Giudice, E. M. and Iafusco, D. | 2020 | Continuous glucose monitoring profile during therapeutic hypothermia in encephalopathic infants with unfavorable outcome | Pediatric Research | 88 | 2 | 218-224 | No feeding outcomes reported |
| Montaldo, P. and Puzone, S. and Caredda, E. and Galdo, F. and Pugliese, U. and Maietta, A. and Ascione, S. and Diplomatico, M. and Spagnuolo, F. and Roma, V. and De Vivo, M. and Carpentieri, M. and Moschella, S. and Giordano, L. and D'Amico, A. and Capristo, C. and Travan, L. and Chello, G. and Miraglia Del Giudice, E. and Cirillo, M. | 2023 | Magnetic Resonance Biomarkers and Neurological Outcome of Infants with Mild Hypoxic-Ischaemic Encephalopathy Who Progress to Moderate Hypoxic-Ischaemic Encephalopathy | Neonatology | 120 | 1 | 153-160 | No feeding outcomes reported |
| Montaldo, Paolo and Lally, Peter J. and Oliveira, Vania and Swamy, Ravi and Mendoza, Josephine and Atreja, Gaurav and Kariholu, Ujwal and Shivamurthappa, Vijayakumar and Liow, Natasha and Teiserskas, Justinas and Pryce, Russell and Soe, Aung and Shankaran, Seetha and Thayyil, Sudhin | 2019 | Therapeutic hypothermia initiated within 6 hours of birth is associated with reduced brain injury on MR biomarkers in mild hypoxic-ischaemic encephalopathy: a non-randomised cohort study | Archives of disease in childhood. Fetal and neonatal edition | 104 | 5 | F515-F520 | No feeding outcomes reported |
| Moorcraft, J. and Bolas, N. M. and Ives, N. K. and Sutton, P. and Blackledge, M. J. and Rajagopalan, B. and Hope, P. L. and Radda, G. K. | 1991 | Spatially localized magnetic resonance spectroscopy of the brains of normal and asphyxiated newborns | Pediatrics | 87 | 3 | 273-82 | No feeding outcomes reported |
| More, K. and Sakhuja, P. and Ting, J. and Seth, J. and Lapointe, A. and McNamara, P. and Moore, A. | 2014 | Changes in visceral blood flow velocities in neonates with hypoxic ischemic encephalopathy during whole body hypothermia and after rewarming | Journal of Paediatrics and Child Health | 50 |  | 23 | Conference abstract |
| Mulkey, S. and Ramakrishnaiah, R. and Bai, S. and Luo, C. and Chang, T. and Mathur, A. and Juul, S. and Wu, Y. | 2016 | Erythropoietin decreases the volume of brain injury in newborns with hypoxic-ischemic encephalopathy | Annals of Neurology | 80 |  | S404 | Conference abstract |
| Mullalli-Bime, G. and Kuli-Lito, G. and Tushe, E. | 2016 | Use of magnesium sulfate in severe perinatal asphyxia and short-term neurologic outcomes | Journal of Maternal-Fetal and Neonatal Medicine | 29 |  | 52 | Conference abstract |
| Munteanu, Andrei Ioan and Manea, Aniko-Maria and Jinca, Cristian Marius and Boia, Marioara | 2021 | Basic biochemical and hematological parameters in perinatal asphyxia and their correlation with hypoxic ischemic encephalopathy | Experimental and therapeutic medicine | 21 | 3 | 259 | No feeding outcomes reported |
| Murray, D. and Boylan, G. and Ryan, C. and Connolly, S. | 2006 | Early continuous video-EEG monitoring and prediction of neurodevelopmental outcome in moderate neonatal hypoxic-ischaemic encephalopathy | Epilepsia | 47 |  | 19-19 | Conference abstract |
| Murray, D. and Boylan, G. and Ryan, C. and Connolly, S. | 2008 | The value of monitoring early EEG evolution in hypoxic-ischaemic encephalopathy and the prediction of neurodevelopmental outcome at 2 years | Acta Paediatrica | 97 |  | 26-26 | Conference abstract |
| Murray, Deirdre M. and Bala, Pronab and O'Connor, Catherine M. and Ryan, C. Anthony and Connolly, Sean and Boylan, Geraldine B. | 2010 | The predictive value of early neurological examination in neonatal hypoxic-ischaemic encephalopathy and neurodevelopmental outcome at 24 months | Developmental medicine and child neurology | 52 | 2 | e55-9 | No analysis of factors influencing HIE feeding outcomes |
| Murray, Deirdre M. and Boylan, Geraldine B. and Ryan, Cornelius A. and Connolly, Sean | 2009 | Early EEG findings in hypoxic-ischemic encephalopathy predict outcomes at 2 years | Pediatrics | 124 | 3 | e459-67 | No feeding outcomes reported |
| Murray, Deirdre M. and O'Riordan, Mairead N. and Horgan, Richard and Boylan, Geraldine and Higgins, John R. and Ryan, Cornelius A. | 2009 | Fetal heart rate patterns in neonatal hypoxic-ischemic encephalopathy: relationship with early cerebral activity and neurodevelopmental outcome | American journal of perinatology | 26 | 8 | 605-12 | No feeding outcomes reported |
| Muttitt, S. C. and Taylor, M. J. and Kobayashi, J. S. and Macmillan, L. and Whyte, H. E. | 1991 | SERIAL VISUAL EVOKED-POTENTIALS AND OUTCOME IN TERM BIRTH ASPHYXIA | Pediatric Neurology | 7 | 2 | 86-90 | No feeding outcomes reported |
| Nadeem, Montasser and Murray, Deirdre and Boylan, Geraldine and Dempsey, Eugene M. and Ryan, C. Anthony | 2010 | Blood carbon dioxide levels and adverse outcome in neonatal hypoxic-ischemic encephalopathy | American journal of perinatology | 27 | 5 | 361-5 | No feeding outcomes reported |
| Nadeem, Montasser and Murray, Deirdre M. and Boylan, Geraldine B. and Dempsey, Eugene M. and Ryan, Cornelius A. | 2011 | Early blood glucose profile and neurodevelopmental outcome at two years in neonatal hypoxic-ischaemic encephalopathy | BMC pediatrics | 11 |  | 10 | No feeding outcomes reported |
| Namusoke, Hellen and Nannyonga, Maria Musoke and Ssebunya, Robert and Nakibuuka, Victoria Kirabira and Mworozi, Edison | 2018 | Incidence and short term outcomes of neonates with hypoxic ischemic encephalopathy in a Peri Urban teaching hospital, Uganda: a prospective cohort study | Maternal health, neonatology and perinatology | 4 |  | 6 | Feeding as part of a composite outcome |
| Narawane, A. and Rappazzo, C. and Hawney, J. and Eng, J. and Ongkasuwan, J. | 2022 | Videofluoroscopic Swallow Study Findings and Correlations in Infancy of Children with Cerebral Palsy | Annals of Otology, Rhinology and Laryngology | 131 | 5 | 478-484 | Mixed cohort |
| Nassar, M. F. and El-Agouza, I. A. and El-Arab, S. E. and Abdel Mohsen, T. F. | 2012 | Fecal calprotectin in hypoxic ischemic neonates: Relation to enteral feeding | Journal of Neonatal-Perinatal Medicine | 5 | 3 | 229-234 | No analysis of breastfeeding or lactation outcomes |
| Natarajan, G. and Hamrick, S. E. and Zaniletti, I. and Lee, K. S. and Mietzsch, U. and DiGeronimo, R. and Dizon, M. L. V. and Peeples, E. S. and Yanowitz, T. D. and Wu, T. W. and Flibotte, J. and Joe, P. and Massaro, A. N. and Rao, R. and Children's Hospitals Neonatal Database Hypoxic-Ischemic Encephalopathy Focus, group | 2022 | Opioid exposure during therapeutic hypothermia and short-term outcomes in neonatal encephalopathy | Journal of Perinatology | 42 | 8 | 1017-1025 | No analysis of breastfeeding or lactation outcomes |
| Natarajan, Girija and Pappas, Athina and Shankaran, Seetha and Laptook, Abbot R. and Walsh, Michele and McDonald, Scott A. and Ehrenkranz, Richard A. and Tyson, Jon E. and Goldberg, Ronald N. and Bara, Rebecca and Higgins, Rosemary D. and Das, Abhik and Munoz, Breda | 2012 | Effect of inborn vs. outborn delivery on neurodevelopmental outcomes in infants with hypoxic-ischemic encephalopathy: secondary analyses of the NICHD whole-body cooling trial | Pediatric research | 72 | 4 | 414-9 | No feeding outcomes reported |
| Naveed, M. and Bondi, D. and Shah, P. | 2021 | Dexmedetomidine versus opioids for neonates with hypoxic ischemic encephalopathy undergoing therapeutic hypothermia | Journal of Pediatric Pharmacology and Therapeutics | 26 | 5 | 518-519 | Conference abstract |
| Naveed, Maryam and Bondi, Deborah S. and Shah, Pooja A. | 2022 | Dexmedetomidine Versus Fentanyl for Neonates With Hypoxic Ischemic Encephalopathy Undergoing Therapeutic Hypothermia | The journal of pediatric pharmacology and therapeutics : JPPT : the official journal of PPAG | 27 | 4 | 352-357 | No analysis of breastfeeding or lactation outcomes |
| Nayak, H. and Panda, S. and Pande, B. and Naik, M. and Hota, P. S. and Hembram, P. K. and Sahoo, S. | 2023 | The Advantageous Impact of Intravenous Magnesium Sulphate in Full-Term Neonates Experiencing Perinatal Asphyxia | International Journal of Pharmaceutical and Clinical Research | 15 | 9 | 472 EP - 477 | Duplicate of data published elsewhere |
| NCT | 2023 | Effect of Non-nutritive Sucking on Transition to Oral Feeding in Infants With Asphyxia | https://clinicaltrials.gov/show/NCT05687708 |  |  |  | Protocol or trial registry entry |
| Nemati, H. and Karimzadeh, P. and Fallahi, M. | 2018 | Causes and factors associated with neonatal seizure and its short-term outcome: A retrospective prognostic cohort study | Iranian Journal of Child Neurology | 12 | 3 | 59-68 | No feeding outcomes reported |
| Nishimaki, S. and Iwasaki, S. and Minamisawa, S. and Seki, K. and Yokota, S. | 2008 | Blood flow velocities in the anterior cerebral artery and basilar artery in asphyxiated infants | Journal of Ultrasound in Medicine | 27 | 6 | 955-960 | No feeding outcomes reported |
| Nonomura, Miho and Harada, Sayaka and Asada, Yuki and Matsumura, Hisako and Iwami, Hiroko and Tanaka, Yuko and Ichiba, Hiroyuki | 2019 | Combination therapy with erythropoietin, magnesium sulfate and hypothermia for hypoxic-ischemic encephalopathy: an open-label pilot study to assess the safety and feasibility | BMC pediatrics | 19 | 1 | 13 | No analysis of factors influencing HIE feeding outcomes |
| Odd, D. and Sabir, H. and Jones, S. A. and Gale, C. and Chakkarapani, E. | 2024 | Risk factors for infection and outcomes in infants with neonatal encephalopathy: a cohort study | Pediatric Research | 96 | 3 | 785 EP - 791 | No analysis of breastfeeding or lactation outcomes |
| Odd, David and Okano, Satomi and Ingram, Jenny and Blair, Peter S. and Billietop, Amiel and Fleming, Peter J. and Thoresen, Marianne and Chakkarapani, Ela | 2021 | Physiological responses to cuddling babies with hypoxic-ischaemic encephalopathy during therapeutic hypothermia: an observational study | BMJ paediatrics open | 5 | 1 |  | No analysis of factors influencing HIE feeding outcomes |
| Oh, William and Perritt, Rebecca and Shankaran, Seetha and Merritts, Matthew and Donovan, Edward F. and Ehrenkranz, Richard A. and O'Shea, T. Michael and Tyson, Jon E. and Laptook, Abbot R. and Das, Abhik and Higgins, Rosemary D. | 2008 | Association between urinary lactate to creatinine ratio and neurodevelopmental outcome in term infants with hypoxic-ischemic encephalopathy | The Journal of Pediatrics | 153 | 3 | 375-8 | No feeding outcomes reported |
| O'Kane, Alex and ra and Vezina, Gilbert and Chang, Taeun and Bendush, Nicole and Ridore, Michel and e and Gai, Jiaxiang and Bost, James and Glass, Penny and Massaro, An N. | 2021 | Early Versus Late Brain Magnetic Resonance Imaging after Neonatal Hypoxic Ischemic Encephalopathy Treated with Therapeutic Hypothermia | The Journal of pediatrics | 232 |  | 73-79.e2 | No feeding outcomes reported |
| Okereafor, Akudo and Allsop, Joanna and Counsell, Serena J. and Fitzpatrick, Julie and Azzopardi, Denis and Rutherford, Mary A. and Cowan, Frances M. | 2008 | Patterns of brain injury in neonates exposed to perinatal sentinel events | Pediatrics | 121 | 5 | 906-14 | No analysis of factors influencing HIE feeding outcomes |
| Oliveira, A. J. and Nunes, M. L. and Haertel, L. M. and Reis, F. M. and Da Costa, J. C. | 2000 | Duration of rhythmic EEG patterns in neonates: New evidence for clinical and prognostic significance of brief rhythmic discharges | Clinical Neurophysiology | 111 | 9 | 1646-1653 | No feeding outcomes reported |
| Oliveira Pereira, Catarina and Dias, Andrea and Nunes Vicente, Ines and Pinto, Joana Teresa and Marques, Carla and Dinis, Alex and ra and Pinto, Carla and Carvalho, Leonor | 2021 | [Prognostic value of near-infrared spectroscopy in hypoxic-ischaemic encephalopathy] | Valor pronostico de la espectroscopia cercana al infrarrojo en la encefalopatia hipoxico-isquemica. | 94 | 3 | 136-143 | No feeding outcomes reported |
| Omran, N. A. E. | 2011 | Correlation between cord blood IL-6 & the severity and outcome of hypoxic ischemmic encephalopathy in asphyxiated noninfected fullterm neonates | Intensive Care Medicine | 37 |  | S382 | Conference abstract |
| Onda, K. and Catenaccio, E. and Chotiyanonta, J. and Chavez-Valdez, R. and Meoded, A. and Soares, B. P. and Tekes, A. and Spahic, H. and Miller, S. C. and Parker, S. J. and Parkinson, C. and Vaidya, D. M. and Graham, E. M. and Stafstrom, C. E. and Everett, A. D. and Northington, F. J. and Oishi, K. | 2022 | Development of a composite diffusion tensor imaging score correlating with short-term neurological status in neonatal hypoxicâ€“ischemic encephalopathy | Frontiers in Neuroscience | 16 |  |  | Feeding as part of a composite outcome |
| O'Neill, S. and DiGeronimo, R. and Rintoul, N. and Zanilet, I. and Chapman, R. and Keene, S. and Seabrook, R. and Dirnberger, D. and Billimoria, Z. and Cleary, J. and Grover, T. and Daniel, J. and Hamrick, S. and Mahmood, B. and Weems, M. and Najaf, T. and Sullivan, K. and Gray, B. | 2020 | Morbidity and mortality of neonates with severe meconium aspiraton syndrome and ECMO: An analysis from the Children's Hospital Neonatal Consortum | ASAIO Journal | 66 |  | 38 | Conference abstract |
| Osredkar, D. and Toet, M. C. and van Rooij, L. G. and van Huffelen, A. C. and Groenendaal, F. and de Vries, L. S. | 2005 | Sleep-wake cycling on amplitude-integrated electroencephalography in term newborns with hypoxic-ischemic encephalopathy | Pediatrics | 115 | 2 | 327-332 | No feeding outcomes reported |
| O'Sullivan, Marc Paul and Casey, Sophie and Finder, Mikael and Ahearne, Caroline and Clarke, Gerard and Hallberg, Boubou and Boylan, Geraldine B. and Murray, Deirdre M. | 2021 | Up-Regulation of Nfat5 mRNA and Fzd4 mRNA as a Marker of Poor Outcome in Neonatal Hypoxic-Ischemic Encephalopathy | The Journal of pediatrics | 228 |  | 74-81.e2 | No feeding outcomes reported |
| O'Sullivan, Marc Paul and Denihan, Niamh and Sikora, Klaudia and Finder, Mikael and Ahearne, Caroline and Clarke, Gerard and Hallberg, Boubou and Boylan, Geraldine B. and Murray, Deirdre M. | 2021 | Activin A and Acvr2b mRNA from Umbilical Cord Blood Are Not Reliable Markers of Mild or Moderate Neonatal Hypoxic-Ischemic Encephalopathy | Neuropediatrics | 52 | 4 | 261-267 | No feeding outcomes reported |
| O'Sullivan, Marc P. and Sikora, Klaudia M. and Ahearne, Caroline and Twomey, Deirdre M. and Finder, Mikael and Boylan, Geraldine B. and Hallberg, Boubou and Murray, Deirdre M. | 2018 | Validation of Raised Cord Blood Interleukin-16 in Perinatal Asphyxia and Neonatal Hypoxic-Ischaemic Encephalopathy in the BiHiVE2 Cohort | Developmental neuroscience | 40 | 3 | 271-277 | No feeding outcomes reported |
| Pandya, F. and Mukherji, A. and Goswami, I. | 2023 | An Exploratory Analysis of Gastrointestinal Morbidities and Feeding Outcomes Associated with Neonatal Hypoxic-Ischemic Encephalopathy With or Without Hypothermia Therapy | Therapeutic Hypothermia and Temperature Management |  |  |  | No analysis of breastfeeding or lactation outcomes |
| Pang, R. and Mujuni, B. M. and Martinello, K. A. and Webb, E. L. and Nalwoga, A. and Ssekyewa, J. and Musoke, M. and Kurinczuk, J. J. and Sewegaba, M. and Cowan, F. M. and Cose, S. and Nakakeeto, M. and Elliott, A. M. and Sebire, N. J. and Klein, N. and Robertson, N. J. and Tann, C. J. | 2022 | Elevated serum IL-10 is associated with severity of neonatal encephalopathy and adverse early childhood outcomes | Pediatric Research | 92 | 1 | 180-189 | No feeding outcomes reported |
| Pardo, Andrea C. | 2015 | Impact of Seizure Burden in Hypoxic Ischemic Encephalopathy | Pediatric neurology briefs | 29 | 10 | 74 | Commentary paper |
| Parish, A. and Bhatia, J. | 2009 | Hypothermia for hypoxicischemic brain injury | Journal of Maternal-Fetal and Neonatal Medicine | 22 | 9 | 719-721 | Review paper |
| Parmentier, C. E. and de Vries, L. S. and Toet, M. C. and van Haastert, I. C. and Koopman, C. and Weeke, L. C. and Groenendaal, F. | 2020 | Increased Use of Therapeutic Hypothermia in Infants with Milder Neonatal Encephalopathy due to Presumed Perinatal Asphyxia | Neonatology | 117 | 4 | 488-494 | No feeding outcomes reported |
| Parmentier, C. E. J. and Lequin, M. H. and Alderliesten, T. and Swanenburg de Veye, H. F. N. and van der Aa, N. E. and Dudink, J. and Benders, M. J. N. L. and Harteman, J. C. and Koopman-Esseboom, C. and Groenendaal, F. and de Vries, L. S. | 2023 | Additional Value of 3-Month Cranial Magnetic Resonance Imaging in Infants with Neonatal Encephalopathy following Perinatal Asphyxia | Journal of Pediatrics | 258 |  | 113402 | No feeding outcomes reported |
| Parmentier, Corline E. J. and de Vries, Linda S. and van der Aa, Niek E. and Eijsermans, Maria J. C. and Harteman, Johanneke C. and Lequin, Maarten H. and Swanenburg de Veye, Henriette F. N. and Koopman-Esseboom, Corine and Groenendaal, Floris | 2022 | Hypoglycemia in Infants with Hypoxic-Ischemic Encephalopathy Is Associated with Additional Brain Injury and Worse Neurodevelopmental Outcome | The Journal of pediatrics | 245 |  | 30-38.e1 | No feeding outcomes reported |
| Parmentier, Corline E. J. and Steggerda, Sylke J. and Weeke, Lauren C. and Rijken, Monique and De Vries, Linda S. and Groenendaal, Floris | 2022 | Outcome of non-cooled asphyxiated infants with under-recognised or delayed-onset encephalopathy | Archives of disease in childhood. Fetal and neonatal edition | 107 | 4 | 364-370 | No feeding outcomes reported |
| Peden, C. J. and Rutherford, M. A. and Sargentoni, J. and Cox, I. J. and Bryant, D. J. and Dubowitz, L. M. S. | 1993 | Proton spectroscopy of the neonatal brain following hypoxic-ischaemic injury | Developmental Medicine and Child Neurology | 35 | 6 | 502-510 | No feeding outcomes reported |
| Peeples, Eric S. and Rao, Rakesh and Dizon, Maria L. V. and Johnson, Yvette R. and Joe, Priscilla and Flibotte, John and Hossain, Tanzeema and Smith, Danielle and Hamrick, Shannon and DiGeronimo, Robert and Natarajan, Girija and Lee, Kyong-Soon and Yanowitz, Toby D. and Mietzsch, Ulrike and Wu, Tai-Wei and Maitre, Nathalie L. and Pallotto, Eugenia K. and Speziale, Mark and Mathur, Amit M. and Zaniletti, Isabella and Massaro, An and Children's Hospitals Neonatal Consortium Hypoxic-Ischemic Encephalopathy Focus, Group | 2021 | Predictive Models of Neurodevelopmental Outcomes After Neonatal Hypoxic-Ischemic Encephalopathy | Pediatrics | 147 | 2 |  | No feeding outcomes reported |
| Pensabene, Licia and Miele, Erasmo and Del Giudice, Ennio and Strisciuglio, Caterina and Staiano, Annamaria | 2008 | Mechanisms of gastroesophageal reflux in children with sequelae of birth asphyxia | Brain & development | 30 | 9 | 563-71 | Gestational age not stated |
| Pereira, S. and Patel, R. and Zaima, A. and Tvarozkova, K. and Chisholm, P. and Kappelou, O. and Evanson, J. and Ch and raharan, E. and Wertheim, D. and Shah, D. K. |  | Physiological CTG categorization in types of hypoxia compared with MRI and neurodevelopmental outcome in infants with HIE | Journal of Maternal-Fetal & Neonatal Medicine |  |  |  | No feeding outcomes reported |
| Pergami, P. and Seemaladinne, N. and Yossuck, P. and Moyers, A. and Lynch, S. and Carpenter, J. | 2012 | Can we predict functional outcome in neonates with hypoxic ischemic encephalopathy by using neuroimaging and EEG? | Neurology | 78 | 1 |  | Conference abstract |
| Phadke, A. K. and Kumble, A. and Ravikumar, K. | 2021 | Early clinical outcome and complications associated in neonates with hypoxic ischemic encephalopathy grade II/III who underwent treatment with servo controlled whole-body therapeutic hypothermia: A prospective observational study | Journal of Clinical Neonatology | 10 | 1 | 24-30 | No analysis of factors influencing HIE feeding outcomes |
| Phillipos, E. and Hendson, L. and Reichert, A. and Kamstra, B. and Molesky, M. | 2014 | Therapeutic hypothermia (TH) for newborns with hypoxic-ischemic encephalopathy (HIE): Audit of the edmonton experience | Paediatrics and Child Health (Canada) | 19 | 6 | e56 | Conference abstract |
| Piecuch, R. and Clyman, R. and Behle, M. and Lang, M. and Ballard, R. | 1987 | PREDICTING NEURODEVELOPMENTAL OUTCOME IN INFANTS WITH SEVERE PERINATAL ASPHYXIA | Pediatric Research | 21 | 4 | A401-A401 | Conference abstract |
| Pierrat, V. and Haouari, N. and Liska, A. and Thomas, D. and Subtil, D. and Truffert, P. | 2005 | Prevalence, causes, and outcome at 2 years of age of newborn encephalopathy: Population based study | Archives of Disease in Childhood: Fetal and Neonatal Edition | 90 | 3 | F257-F261 | No analysis of factors influencing HIE feeding outcomes |
| Pinto, C. R. and Duarte, J. V. and Marques, C. and Vicente, I. N. and Paiva, C. and Eloi, J. and Pereira, D. J. and Correia, B. R. and Castelo-Branco, M. and Oliveira, G. | 2023 | The role of early functional neuroimaging in predicting neurodevelopmental outcomes in neonatal encephalopathy | European Journal of Pediatrics | 182 | 3 | 1191-1200 | No analysis of factors influencing HIE feeding outcomes |
| Polam, S. and Koons, A. and Anwar, M. and Shen-Schwartz, S. and Hiatt, M. and Hegyi, T. | 2002 | Histologic chorioamnionitis, neonatal brain injury and neurodevelopmental outcome in preterm infants | Pediatric Research | 51 | 4 | 368A-368A | Conference abstract |
| Polat, Muzaffer and Simsek, Ayse and Tansug, Nermin and Sezer, Rabia G. and Ozkol, Mine and Baspinar, Pinar and Tekgul, Hasan | 2013 | Prediction of neurodevelopmental outcome in term neonates with hypoxic-ischemic encephalopathy | European journal of paediatric neurology : EJPN : official journal of the European Paediatric Neurology Society | 17 | 3 | 288-93 | No feeding outcomes reported |
| Power, B. D. and Slevin, M. and Donoghue, V. and Sweetman, D. and Murphy, J. F. A. | 2021 | Neonatal therapeutic hypothermia for neonatal encephalopathy: Mortality and neurodevelopmental outcome | Irish Medical Journal | 114 | 2 | P264 | No feeding outcomes reported |
| Pressler, R. M. and Boylan, G. B. and Morton, M. and Binnie, C. D. and Rennie, J. M. | 2001 | Early serial EEG in hypoxic ischaemic encephalopathy | Clinical Neurophysiology | 112 | 1 | 31-37 | No feeding outcomes reported |
| Prial, Jennifer and El-Shibiny, Hoda and El-Dib, Mohamed and Benjamin, Jennifer and Erdei, Carmina and Dodrill, Pamela and Szakmar, Eniko and Bell, Katherine A. | 2024 | Growth trajectories and need for oral feeding support among infants with neonatal encephalopathy treated with therapeutic hypothermia | Journal of Perinatology | 44 | 8 | 1163-1171 | No analysis of breastfeeding or lactation outcomes |
| Primhak, R. A. and Simmonds, E. | 1991 | First day serum creatine kinase BB isoenzyme in high-risk infants | European Journal of Pediatrics | 150 | 4 | 271-273 | No feeding outcomes reported |
| Puthuraya, S. and Karnati, S. and Sripathi, R. and Acun, C. and Padiyar, S. and Aly, H. | 2022 | Time Until Recovery from Metabolic Acidemia: A Prognostic Marker for Perinatal Hypoxic-ischemic Encephalopathy (HIE)? | Pediatrics | 149 |  |  | Conference abstract |
| Qian, T. Y. and Gao, T. and Qiu, H. and Zhang, P. and Cheng, G. Q. and Wang, L. S. | 2023 | Clinical characteristic and risk factors for feeding difficulties in neonates with hypoxic-ischemic encephalopathy | Fudan University Journal of Medical Sciences | 50 | 3 | 405-411 | Feeding as part of a composite outcome |
| Quattrocchi, C. C. and Longo, D. and Delfino, L. N. and Cilio, M. R. and Piersigilli, F. and Capua, M. D. and Seganti, G. and Danhaive, O. and Fariello, G. | 2010 | Dorsal brain stem syndrome: MR imaging location of brain stem tegmental lesions in neonates with oral motor dysfunction | AJNR. American journal of neuroradiology | 31 | 8 | 1438-42 | Mixed cohort |
| Quinn, Megan and Banta-Wright, Sandra and Warren, Jamie B. | 2025 | Influences of a Remote Monitoring Program of Home Nasogastric Tube Feeds on Transition from NICU to Home | American Journal of Perinatology | 42 | 2 | 250-255 | Mixed cohort |
| Radford, S. and Henderson, D. and Panjwani, D. and Satodia, P. | 2016 | Blood hyperlactaemia as a predictor of morbidity and mortality in infants with hypoxic ischaemic encephalopathy | Journal of Maternal-Fetal and Neonatal Medicine | 29 |  | 239-240 | Conference abstract |
| Ramirez, Alice and Peyv and i, Shabnam and Cox, Stephany and Gano, Dawn and Xu, Duan and Tymofiyeva, Olga and McQuillen, Patrick S. | 2022 | Neonatal brain injury influences structural connectivity and childhood functional outcomes | PloS one | 17 | 1 | e0262310 | No feeding outcomes reported |
| Ravich and ran, Lavanya and Allen, Victoria M. and Allen, Alex and er C. and Vincer, Michael and Baskett, Thomas F. and Woolcott, Christy G. | 2020 | Incidence, Intrapartum Risk Factors, and Prognosis of Neonatal Hypoxic-Ischemic Encephalopathy Among Infants Born at 35 Weeks Gestation or More | Journal of obstetrics and gynaecology Canada : JOGC = Journal d'obstetrique et gynecologie du Canada : JOGC | 42 | 12 | 1489-1497 | No feeding outcomes reported |
| Ravich and ran, L. and Allen, V. M. and Vincer, M. and Kuhle, S. and Filliter, C. and Baskett, T. F. | 2019 | Intrapartum Characteristics and Prognosis Associated with Neonatal Hypoxic-Ischemic Encephalopathy | Obstetrics and Gynecology | 133 |  |  | Conference abstract |
| Reddy, Ravikanth | 2022 | Magnetic Resonance Imaging Evaluation of Perinatal Hypoxic Ischemic Encephalopathy: An Institutional Experience | Journal of neurosciences in rural practice | 13 | 1 | 87-94 | No feeding outcomes reported |
| Reiss, Jonathan and Sinha, Mridu and Gold, Jeffrey and Bykowski, Julie and Lawrence, Shelley M. | 2019 | Outcomes of Infants with Mild Hypoxic Ischemic Encephalopathy Who Did Not Receive Therapeutic Hypothermia | Biomedicine hub | 4 | 3 | 01-Sep | No feeding outcomes reported |
| Robertson, N. J. and Cox, I. J. and Cowan, F. M. and Counsell, S. J. and Azzopardi, D. and Edwards, A. D. | 1999 | Cerebral intracellular lactic alkalosis persisting months after neonatal encephalopathy measured by magnetic resonance spectroscopy | Pediatric Research | 46 | 3 | 287-296 | No feeding outcomes reported |
| Rogers, E. E. and Glass, H. C. and Bonifacio, S. L. and Chang, T. and Mayock, D. and Dur and , D. J. and Song, D. and Ballard, R. A. and Wu, Y. W. | 2013 | Neurodevelopmental outcomes after treatment with erythropoietin in a pilot trial for perinatal hypoxic-ischemic encephalopathy | Journal of Investigative Medicine | 61 | 1 | 167 | Conference abstract |
| Roka, A. and Dorottya, K. and Halasz, J. and Beko, G. and Azzopardi, D. and Szabo, M. | 2012 | Serum S100B and neuron-specific enolase levels in normothermic and hypothermic infants after perinatal asphyxia | Acta Paediatrica | 101 | 3 | 319-323 | No feeding outcomes reported |
| Roland , E. H. and Poskitt, K. and Rodriguez, E. and Lupton, B. A. and Hill, A. | 1998 | Perinatal hypoxic-ischemic thalamic injury: Clinical features and neuroimaging | Annals of Neurology | 44 | 2 | 161-166 | No analysis of factors influencing HIE feeding outcomes |
| Romeo, Domenico M. and Bompard, Sarah and Serrao, Francesca and Leo, Giuseppina and Cicala, Gianpaolo and Velli, Chiara and Gallini, Francesca and Priolo, Francesca and Vento, Giovanni and Mercuri, Eugenio | 2019 | Early Neurological Assessment in Infants with Hypoxic Ischemic Encephalopathy Treated with Therapeutic Hypothermia | Journal of clinical medicine | 8 | 8 |  | No feeding outcomes reported |
| Ross, M. N. and Haase, G. M. and Reiley, T. T. and Meagher Jr, D. P. | 1988 | The importance of acid reflux patterns in neurologically damaged children detected by four-channel esophageal pH monitoring | Journal of Pediatric Surgery | 23 | 6 | 573-576 | No analysis of factors influencing HIE feeding outcomes |
| Roth, S. and Baudin, J. and Cady, E. and Townsend, J. and Wyatt, J. and Reynolds, O. and Stewart, A. | 1995 | DERANGED CEREBRAL OXIDATIVE-PHOSPHORYLATION FOLLOWING BIRTH ASPHYXIA AND NEURODEVELOPMENTAL OUTCOME AT 4 YEARS | Pediatric Research | 38 | 3 | 452-452 | Conference abstract |
| Roth, S. C. and Baudin, J. and Cady, E. and Johal, K. and Townsend, J. P. and Wyatt, J. S. and Reynolds, E. O. and Stewart, A. L. | 1997 | Relation of deranged neonatal cerebral oxidative metabolism with neurodevelopmental outcome and head circumference at 4 years | Developmental medicine and child neurology | 39 | 11 | 718-25 | No feeding outcomes reported |
| Roth, Simon C. and Edwards, A. David and Cady, Ernest B. and Delpy, David T. and Wyatt, John S. and Azzopardi, Denis and Baudin, Jenny and Townsend, Jan and Stewart, Ann L. and Reynolds, E. Osmund R. and Roth, S. C. and Edwards, A. D. and Cady, E. B. and Delpy, D. T. and Wyatt, J. S. and Azzopardi, D. and Baudin, J. and Townsend, J. and Stewart, A. L. and Reynolds, E. O. | 1992 | Relation between cerebral oxidative metabolism following birth asphyxia, and neurodevelopmental outcome and brain growth at one year | Developmental Medicine & Child Neurology | 34 | 4 | 285-295 | No feeding outcomes reported |
| Round, A. and McWilliam, M. and Chang, R. and Derwas, E. and Iyer, A. | 2016 | Electro-clinical features and outcomes in neonatal burst suppression pattern EEG | Developmental Medicine and Child Neurology | 58 |  | 41 | Conference abstract |
| Rutherford, M. A. and Pennock, J. M. and Counsell, S. J. and Mercuri, E. and Cowan, F. M. and Dubowitz, L. M. S. and Edwards, A. D. | 1998 | Abnormal magnetic resonance signal in the internal capsule predicts poor neurodevelopmental outcome in infants with hypoxic-ischemic encephalopathy | Pediatrics | 102 | 2 | 323-328 | No analysis of factors influencing HIE feeding outcomes |
| Saket, Sasan and Karimzadeh, Parvaneh and Nasehi, Mohammad Mehdi and Taghdiri, Mohammad Mehdi and Falahi, Minoo and Shamshiri, Ahmadreza and Rahimian, Elham | 2022 | Neuroimaging Findings of the High-risk Neonates and Infants Referred to Mofid Children's Hospital | Iranian Journal of Child Neurology | 16 | 4 | 33-44 | No feeding outcomes reported |
| Sakhuja, Pankaj and More, Kiran and Ting, Joseph Y. and Sheth, Jesal and Lapointe, Annie and Jain, Amish and McNamara, Patrick J. and Moore, Aideen M. | 2019 | Gastrointestinal hemodynamic changes during therapeutic hypothermia and after rewarming in neonatal hypoxic-Ischemic encephalopathy | Pediatrics and neonatology | 60 | 6 | 669-675 | No analysis of factors influencing HIE feeding outcomes |
| Salamah, A. and El Amrousy, D. and Elsheikh, M. and Mehrez, M. | 2023 | Citicoline in hypoxic ischemic encephalopathy in neonates: a randomized controlled trial | Italian Journal of Pediatrics | 49 | 1 | 55 | No feeding outcomes reported |
| Sanjeevappa, M. and Monalisa, Z. H. and Mamatha, P. N. and Prathyusha, C. V. | 2023 | A study of neurosonogram findings in new borns with hypoxic ischemic encephalopathy and their correlation with neurodevelopmental outcome | Journal of Cardiovascular Disease Research | 14 | 3 | 294 EP - 298 | Article not locatable |
| Sarafidis, K. and Soubasi, V. and Diamanti, E. and Mitsakis, K. and Drossou-Agakidou, V. | 2014 | Therapeutic hypothermia in asphyxiated neonates with hypoxic-ischemic encephalopathy: A single-center experience from its first application in Greece | Hippokratia | 18 | 3 | 226-30 | No feeding outcomes reported |
| Sarkar, S. and Barks, J. D. and Bhagat, I. and Bapuraj, J. R. and Dechert, R. E. and Donn, S. M. | 2012 | Does clinical status one week after therapeutic hypothermia predict brain MRI abnormalities? | Brain Injury | 26 | 4 | 333-334 | Conference abstract |
| Sarkar, S. and Bhagat, I. and Bapuraj, J. R. and Dechert, R. E. and Donn, S. M. | 2013 | Does clinical status 1 week after therapeutic hypothermia predict brain MRI abnormalities? | Journal of perinatology : official journal of the California Perinatal Association | 33 | 7 | 538-42 | No analysis of breastfeeding or lactation outcomes |
| Scaramuzzo, Rosa T. and Giampietri, Matteo and Fiorentini, Erika and Bartalena, Laura and Fiori, Simona and Guzzetta, Andrea and Ciampi, Mariella and Boldrini, Antonio and Ghirri, Paolo | 2015 | Serum cortisol concentrations during induced hypothermia for perinatal asphyxia are associated with neurological outcome in human infants | Stress (Amsterdam, Netherlands) | 18 | 1 | 129-33 | No feeding outcomes reported |
| Scherer, Jonas and Whybra-Trumpler, Catharina and Mildenberger, Eva | 2020 | [Results of the hypothermia registry of the University Hospital Dresden from 2010-2017. Application of hypothermia therapy in newborns in Germany] | Ergebnisse des Hypothermienetzwerks des Universitatsklinikums Dresden von 2010-2017. Entwicklung der Hypothermietherapie bei Neugeborenen in Deutschland. | 224 | 6 | 367-373 | No analysis of factors influencing HIE feeding outcomes |
| Seo, Yu-Mi and Im, Soo-Ah and Sung, In Kyung and Youn, Young Ah | 2020 | The prognosis of brain magnetic resonance imaging injury pattern for outcomes of hypothermia-treated infants | Medicine | 99 | 48 | e23176 | No analysis of breastfeeding or lactation outcomes |
| Sepeng, Letlhogonolo | 2016 | Audit of feeding practices in the neonatal wards at the Charlotte Maxeke Johannesburg academic hospital |  |  |  |  | No analysis of factors influencing HIE feeding outcomes |
| Sepeng, L. and Ballot, D. E. | 2015 | Audit of feeding practices in the neonatal wards at the charlotte maxeke Johannesburg academic hospital | SAJCH South African Journal of Child Health | 9 | 4 | 133-136 | No analysis of factors influencing HIE feeding outcomes |
| Serdaroglu, G. and Tekgul, H. and Kitis, O. and Serdaroglu, E. and Gokben, S. | 2004 | Correlative value of magnetic resonance imaging for neurodevelopmental outcome in periventricular leukomalacia | Developmental Medicine and Child Neurology | 46 | 11 | 733-739 | No feeding outcomes reported |
| Sewell, E. K. and Shankaran, S. and McDonald, S. A. and Hamrick, S. and Wusthoff, C. J. and Adams-Chapman, I. and Chalak, L. F. and Davis, A. S. and Van Meurs, K. and Das, A. and Maitre, N. and Laptook, A. and Patel, R. M. | 2023 | Antiseizure medication at discharge in infants with hypoxic-ischaemic encephalopathy: An observational study | Archives of Disease in Childhood: Fetal and Neonatal Edition | 108 | 4 | 421-428 | No analysis of breastfeeding or lactation outcomes |
| Sewell, E. K. and Vezina, G. and Chang, T. and Tsuchida, T. and Harris, K. and Ridore, M. and Glass, P. and Massaro, A. N. | 2018 | Evolution of Amplitude-Integrated Electroencephalogram as a Predictor of Outcome in Term Encephalopathic Neonates Receiving Therapeutic Hypothermia | American Journal of Perinatology | 35 | 3 | 277-285 | No feeding outcomes reported |
| Shah, Divyen K. and Yip, Ping K. and Barlas, Akif and Tharmapoopathy, Pavithira and Ponnusamy, Vennila and Michael-Titus, Adina T. and Chisholm, Philippa | 2020 | Raised Plasma Neurofilament Light Protein Levels After Rewarming Are Associated With Adverse Neurodevelopmental Outcomes in Newborns After Therapeutic Hypothermia | Frontiers in neurology | 11 |  | 562510 | No feeding outcomes reported |
| Shah, Prakeshkumar | 2005 | Prediction of outcome within the first four hours after birth of a term infant with post-asphyxial hypoxic ischemic encephalopathy |  |  |  |  | No feeding outcomes reported |
| Shah, S. and Fern and ez, A. R. and Chirla, D. | 2001 | Role of brain SPECT in neonates with hypoxic ischemic encephalopathy and its correlation with neurodevelopmental outcome | Indian pediatrics | 38 | 7 | 705-13 | Feeding as part of a composite outcome |
| Shankaran, S. and Laptook, A. R. and Ehrenkranz, R. A. and Tyson, J. E. and McDonald, S. A. and Donovan, E. F. and Fanaroff, A. A. and Poole, W. K. and Wright, L. L. and Higgins, R. D. and Finer, N. N. and Carlo, W. A. and Duara, S. and Oh, W. and Cotten, C. M. and Stevenson, D. K. and Stoll, B. J. and Lemons, J. A. and Guillet, R. and Jobe, A. H. | 2005 | Whole-body hypothermia for neonates with hypoxic-ischemic encephalopathy | New England Journal of Medicine | 353 | 15 | 1574-1584 | No analysis of breastfeeding or lactation outcomes |
| Shankaran, S. and Laptook, A. R. and McDonald, S. A. and Hintz, S. R. and Barnes, P. D. and Das, A. and Higgins, R. D. | 2017 | Acute Perinatal Sentinel Events, Neonatal Brain Injury Pattern, and Outcome of Infants Undergoing a Trial of Hypothermia for Neonatal Hypoxic-Ischemic Encephalopathy | Journal of Pediatrics | 180 |  | 275-278.e2 | No feeding outcomes reported |
| Shany, E. and Goldstein, E. and Khvatskin, S. and Friger, M. D. and Heiman, N. and Goldstein, M. and Karplus, M. and Galil, A. | 2006 | Predictive Value of Amplitude-Integrated Electroencephalography Pattern and Voltage in Asphyxiated Term Infants | Pediatric Neurology | 35 | 5 | 335-342 | No feeding outcomes reported |
| Shellhaas, Renee A. and Kushwaha, Juhi S. and Plegue, Melissa A. and Selewski, David T. and Barks, John D. E. | 2015 | An Evaluation of Cerebral and Systemic Predictors of 18-Month Outcomes for Neonates With Hypoxic Ischemic Encephalopathy | Journal of child neurology | 30 | 11 | 1526-31 | No feeding outcomes reported |
| Shibasaki, J. and Aida, N. and Morisaki, N. and Tomiyasu, M. and Nishi, Y. and Toyoshima, K. | 2018 | Changes in brain metabolite concentrations after neonatal hypoxic-ischemic encephalopathy | Radiology | 288 | 3 | 840-848 | Feeding as part of a composite outcome |
| Shibasaki, J. and Mukai, T. and Tsuda, K. and Takeuchi, A. and Ioroi, T. and Sano, H. and Yutaka, N. and Takahashi, A. and Sobajima, H. and Tamura, M. and Hosono, S. and Nabetani, M. and Iwata, O. | 2020 | Outcomes related to 10-min Apgar scores of zero in Japan | Archives of Disease in Childhood: Fetal and Neonatal Edition | 105 | 1 | F64-F68 | No analysis of factors influencing HIE feeding outcomes |
| Shibasaki, Jun and Niwa, Tetsu and Piedvache, Aurelie and Tomiyasu, Moyoko and Morisaki, Naho and Fujii, Yuta and Toyoshima, Katsuaki and Aida, Noriko | 2021 | Comparison of Predictive Values of Magnetic Resonance Biomarkers Based on Scan Timing in Neonatal Encephalopathy Following Therapeutic Hypothermia | The Journal of pediatrics | 239 |  | 101-109.e4 | No feeding outcomes reported |
| Shubert, T. and Sitaram, S. and Jadcherla, S. | 2016 | Esophageal dysmotility mechanisms in infants with birth asphyxia may reflect heightened excitability of liquid-sensitive reflexes | Gastroenterology | 150 | 4 | S287-S288 | Conference abstract |
| Shubert, T. and Sitaram, S. and Jadcherla, S. | 2016 | Esophageal bolus volume-dependent upper esophageal sphincter (UES) and lower esophageal sphincter (LES) reflexes in infants with birth asphyxia | Gastroenterology | 150 | 4 | S228 | Conference abstract |
| Sibartie, P. and Crowley, P. and Fitzpatrick, C. | 2011 | Umbilical artery ph: Is it a good predictor of HIE and adverse neonatal outcome? | Irish Journal of Medical Science | 180 |  | S121-S122 | Conference abstract |
| Siddiqui, Muhammad Asif and Butt, Tayyaba Khawar | 2021 | Role of Intravenous Magnesium Sulphate in Term Neonates with Hypoxic Ischemic Encephalopathy (HIE) in a Low-income Country: A Randomised Clinical Trial | Journal of the College of Physicians and Surgeons--Pakistan : JCPSP | 30 | 7 | 817-820 | No analysis of breastfeeding or lactation outcomes |
| Simbron, Alicia V. and Sorbera-Ferrer, Lucas and Gomez de Ferraris, Maria E. and Carranza, Miriam L. | 2013 | Cyclical pattern of non-nutritive sucking in normal and high-risk neonates | Acta odontologica latinoamericana : AOL | 26 | 3 | 150-4 | No analysis of factors influencing HIE feeding outcomes |
| Singh, A. and Saluja, S. and Kler, N. and Garg, P. and Soni, A. and Thakur, A. | 2021 | Amplitude integrated EEG: how much it helps in prognostication in neonatal encephalopathy? | Journal of Maternal-Fetal and Neonatal Medicine |  |  |  | Mixed cohort |
| Sivakanthan, S. and Crane, D. and Ahmed, E. and John, N. and Akubuiro, C. and Das, A. | 2019 | Outcome trend of hypoxic ischemic encephalopathy in term and near-term neonates | Cogent Medicine | 6 | 1 |  | Conference abstract |
| Skoromets, A. P. and Schugareva, L. M. and Shumilina, M. V. and Gorelik, Yuu V. | 2016 | [The improvement of treatment efficacy in newborn full-term infants with severe birth asphyxia] | Povyshenie effektivnosti terapii novorozhdennykh donoshennykh detei s tyazheloi asfiksiei v rodakh. | 116 | 4 | 83-88 | No analysis of breastfeeding or lactation outcomes |
| Sofijanova, A. N. and Jordanova, O. V. and Piperkova, K. | 2012 | Initial experience of effect of selective head cooling in normothermic and hypothermic infants | Archives of Disease in Childhood | 97 |  | A306 | Conference abstract |
| Sowjanya, Svns and Venugopalan, L. and Thiagarajan, K. | 2016 | Therapeutic hypothermia for perinatal asphyxia in an Urban Tertiary Referral Center in South India: Our experience | Journal of Clinical Neonatology | 5 | 3 | 150-152 | No analysis of factors influencing HIE feeding outcomes |
| Stark, J. E. and Seibert, J. J. | 1994 | Cerebral artery Doppler ultrasonography for prediction of outcome after perinatal asphyxia | Journal of Ultrasound in Medicine | 13 | 8 | 595-600 | No feeding outcomes reported |
| Stelmach, T. and Kallas, E. and Pisarev, H. and Talvik, T. | 2004 | Antenatal risk factors associated with unfavorable neurologic status in newborns and at 2 years of age | Journal of Child Neurology | 19 | 2 | 116-122 | No feeding outcomes reported |
| Stetson, R. C. and Brumbaugh, J. E. and Weaver, A. L. and Mara, K. C. and Clark, R. H. and Carey, W. A. and Fang, J. L. | 2021 | Association of outborn versus inborn birth status on the in-hospital outcomes of neonates treated with therapeutic hypothermia: A propensity score-weighted cohort study | Resuscitation | 167 |  | 82-88 | No analysis of breastfeeding or lactation outcomes |
| Stone, A. C. and Strickl and , K. C. and Tanaka, D. T. and Gilner, J. B. and Lemmon, M. E. and Russ, J. B. | 2023 | The association of placental pathology and neurodevelopmental outcomes in patients with neonatal encephalopathy | Pediatric Research |  |  |  | Gestational age not stated |
| Sun, M. and Wang, W. L. and Wang, W. and Wen, D. L. and Zhang, H. and Han, Y. K. | 2001 | Gastroesophageal manometry and 24-hour double pH monitoring in neonates with birth asphyxia | World journal of gastroenterology | 7 | 5 | 695-7 | Not definitive HIE diagnosis |
| Sweetman, D. U. and Lakatos, P. and Molloy, E. J. and Kardasi, J. and Bango, M. and Szabo, M. | 2012 | Poor motor outcome at 2 years of age is predicted by elevated leukocyte count in infants with perinatal asphyxia | Archives of Disease in Childhood | 97 |  | A304 | Conference abstract |
| Syvalahti, T. and Tuiskula, A. and Nevalainen, P. and Metsaranta, M. and Haataja, L. and Vanhatalo, S. and Tokariev, A. | 2024 | Networks of cortical activity show graded responses to perinatal asphyxia | Pediatric Research | 96 | 1 | 132 EP - 140 | No analysis of breastfeeding or lactation outcomes |
| Takenouchi, T. and Cuaycong, M. and Ross, G. and Engel, M. and Perlman, J. M. | 2010 | Chain of brain preservation-A concept to facilitate early identification and initiation of hypothermia to infants at high risk for brain injury | Resuscitation | 81 | 12 | 1637-1641 | No feeding outcomes reported |
| Takle, M. and Conaway, M. and Burnsed, J. | 2021 | Electroencephalogram Background Predicts Time to Full Oral Feedings in Hypoxic-Ischemic Encephalopathy | American Journal of Perinatology |  |  |  | No analysis of breastfeeding or lactation outcomes |
| Tan, Jason Khay Ghim and Minutillo, Corrado and McMichael, Judy and Rao, Shripada | 2017 | Impact of hypoglycaemia on neurodevelopmental outcomes in hypoxic ischaemic encephalopathy: a retrospective cohort study | BMJ paediatrics open | 1 | 1 | e000175 | No feeding outcomes reported |
| Tanaka, M. and Kidokoro, H. and Kubota, T. and Fukasawa, T. and Okai, Y. and Sakaguchi, Y. and Ito, Y. and Yamamoto, H. and Ohno, A. and Nakata, T. and Negoro, T. and Okumura, A. and Kato, T. and Watanabe, K. and Takahashi, Y. and Natsume, J. | 2020 | Pseudo-sawtooth pattern on amplitude-integrated electroencephalography in neonatal hypoxic-ischemic encephalopathy | Pediatric Research | 87 | 3 | 529-535 | No feeding outcomes reported |
| Tang, Z. and Mahmoodi, S. and Meng, D. and Darekar, A. and Vollmer, B. | 2024 | Rule-based deep learning method for prognosis of neonatal hypoxic-ischemic encephalopathy by using susceptibility weighted image analysis | Magma | 37 | 2 | 227 EP - 239 | No feeding outcomes reported |
| Tanigasalam, Vasanthan and Plakkal, Nishad and Vishnu Bhat, B. and Chinnakali, Palanivel | 2018 | Does fluid restriction improve outcomes in infants with hypoxic ischemic encephalopathy? A pilot randomized controlled trial | Journal of Perinatology | 38 | 11 | 1512-1517 | No analysis of breastfeeding or lactation outcomes |
| Tayman, C. and Oztekin, O. and Serkant, U. and Yakut, I. and Aydemir, S. and Kosus, A. | 2017 | Ischemia-Modified Albumin May Be a Novel Marker for Predicting Neonatal Neurologic Injury in Small-For-Gestational-Age Infants in Addition to Neuron-Specific Enolase | American Journal of Perinatology | 34 | 4 | 349-358 | No feeding outcomes reported |
| Tekgul, H. and Gauvreau, K. and Soul, J. and Murphy, L. and Robertson, R. and Stewart, J. and Volpe, J. and Bourgeois, B. and du Plessis, A. J. | 2006 | The current etiologic profile and neurodevelopmental outcome of seizures in term newborn infants | Pediatrics | 117 | 4 | 1270-1280 | No feeding outcomes reported |
| Temko, Andriy and Doyle, Orla and Murray, Deirdre and Lightbody, Gordon and Boylan, Geraldine and Marnane, William | 2015 | Multimodal predictor of neurodevelopmental outcome in newborns with hypoxic-ischaemic encephalopathy | Computers in biology and medicine | 63 |  | 169-77 | No feeding outcomes reported |
| Thompson, C. M. and Puterman, A. S. and Linley, L. L. and Hann, F. M. and v and erElst, C. W. and Molteno, C. D. and Malan, A. F. | 1997 | The value of a scoring system for hypoxic ischaemic encephalopathy in predicting neurodevelopmental outcome | Acta Paediatrica | 86 | 7 | 757-761 | No feeding outcomes reported |
| Thoresen, Marianne and Jary, Sally and Walloe, Lars and Karlsson, Mathias and Martinez-Biarge, Miriam and Chakkarapani, Ela and Cowan, Frances M. | 2021 | MRI combined with early clinical variables are excellent outcome predictors for newborn infants undergoing therapeutic hypothermia after perinatal asphyxia | EClinicalMedicine | 36 |  | 100885 | No analysis of factors influencing HIE feeding outcomes |
| Thornton, Kimberly M. and Dai, Hongying and Septer, Seth and Petrikin, Joshua E. | 2014 | Effects of whole body therapeutic hypothermia on gastrointestinal morbidity and feeding tolerance in infants with hypoxic ischemic encephalopathy | International journal of pediatrics | 2014 |  | 643689 | No analysis of breastfeeding or lactation outcomes |
| Tillqvist, E. and Thyagarajan, B. and Baral, V. and Hallberg, B. and Vollmer, B. and Blennow, M. | 2012 | An evaluation of the use of enteral nutrition during hypothermia treatment for perinatal hypoxic ischaemic encephalopathy | Archives of Disease in Childhood | 97 |  | A318 | Conference abstract |
| Toet, Mona C. and Groenendaal, Floris and Osredkar, Damjan and van Huffelen, Alex and er C. and de Vries, Linda S. | 2005 | Postneonatal epilepsy following amplitude-integrated EEG-detected neonatal seizures | Pediatric neurology | 32 | 4 | 241-7 | No feeding outcomes reported |
| Toh, V. and Rajadurai, V. S. | 1999 | Term infants with hypoxic ischaemic encephalopathy: poor neurodevelopmental outcome despite standard neonatal intensive care | Journal of tropical pediatrics | 45 | 4 | 229-32 | No feeding outcomes reported |
| Tokariev, Anton | 2024 | Networks of cortical activity show graded responses to perinatal asphyxia: Aivokuoren toiminnallisissa verkostoissa nÃ¤kyy asteittaisia muutoksia syntymÃ¤asfyksian jÃ¤lkeen | http://hdl.handle.net/10138/571589 |  |  |  | Duplicate |
| Trotman, Helen and Garbutt, Andrea | 2011 | Predictors of outcome of neonates with hypoxic ischaemic encephalopathy admitted to the neonatal unit of the University Hospital of the West Indies | Journal of tropical pediatrics | 57 | 1 | 40-4 | No analysis of factors influencing HIE feeding outcomes |
| Tsuda, K. and Mukai, T. and Iwata, S. and Shibasaki, J. and Tokuhisa, T. and Ioroi, T. and Sano, H. and Yutaka, N. and Takahashi, A. and Takeuchi, A. and Takenouchi, T. and Araki, Y. and Sobajima, H. and Tamura, M. and Hosono, S. and Nabetani, M. and Iwata, O. and Baby Cooling Registry, Japan | 2017 | Therapeutic hypothermia for neonatal encephalopathy: a report from the first 3 years of the Baby Cooling Registry of Japan | Scientific Reports | 7 |  |  | No analysis of factors influencing HIE feeding outcomes |
| Tsuda, K. and Shibasaki, J. and Isayama, T. and Takeuchi, A. and Mukai, T. and Ioroi, T. and Takahashi, A. and Sano, H. and Yutaka, N. and Iwata, S. and Nabetani, M. and Sobajima, H. and Hosono, S. and Tamura, M. and Iwata, O. and the Baby Cooling Registry of, Japan | 2022 | Body temperature, heart rate and long-term outcome of cooled infants: an observational study | Pediatric Research | 91 | 4 | 921-928 | No analysis of factors influencing HIE feeding outcomes |
| Tsuda, K. and Shibasaki, J. and Takeuchi, A. and Mukai, T. and Sugiyama, Y. and Isayama, T. and Ioroi, T. and Takahashi, A. and Yutaka, N. and Iwata, O. | 2023 | Prolonged requirements for mechanical ventilation and tube feeding support predicted 18-month outcomes for neonatal encephalopathy | Acta Paediatrica, International Journal of Paediatrics | 112 | 4 | 734-741 | Feeding as part of a composite outcome |
| Tuiskula, A. and Haataja, L. and Metsaranta, M. | 2025 | Measuring the time it takes to achieve full oral feeding can be used as a low-resource tool to assess neurologic recovery after perinatal asphyxia | Journal of Tropical Pediatrics | 71 | 2 | fmaf012 | No analysis of breastfeeding or lactation outcomes |
| Tuiskula, A. and Pospelov, A. S. and Nevalainen, P. and Montazeri, S. and Metsaranta, M. and Haataja, L. and Stevenson, N. and Tokariev, A. and Vanhatalo, S. | 2025 | Quantitative EEG features during the first day correlate to clinical outcome in perinatal asphyxia | Pediatric Research | 97 | 1 | 261 EP - 267 | No analysis of breastfeeding or lactation outcomes |
| Tusor, Nora and Wusthoff, Courtney and Smee, Natalie and Merchant, Nazakat and Arichi, Tomoki and Allsop, Joanna M. and Cowan, Frances M. and Azzopardi, Denis and Edwards, A. David and Counsell, Serena J. | 2012 | Prediction of neurodevelopmental outcome after hypoxic-ischemic encephalopathy treated with hypothermia by diffusion tensor imaging analyzed using tract-based spatial statistics | Pediatric research | 72 | 1 | 63-9 | No feeding outcomes reported |
| Twomey, Eilish and Twomey, Anne and Ryan, Stephanie and Murphy, John and Donoghue, Veronica B. | 2010 | MR imaging of term infants with hypoxic-ischaemic encephalopathy as a predictor of neurodevelopmental outcome and late MRI appearances | Pediatric radiology | 40 | 9 | 1526-35 | No feeding outcomes reported |
| Van Bel, F. and Lemmers, P. L. and De Vries, L. S. and Groenendaal, F. and Benders, M. J. N. L. and Zwanenburg, R. and Toet, M. C. | 2011 | Does hypothermia change the relation between cerebral oxygenation and electrical activity with outcome after perinatal asphyxia? | Journal of Neonatal-Perinatal Medicine | 4 | 3 | 300-301 | Conference abstract |
| van Rooij, Linda G. M. and de Vries, Linda S. and H and ryastuti, Setyo and Hawani, Dewi and Groenendaal, Floris and van Huffelen, Alex and er C. and Toet, Mona C. | 2007 | Neurodevelopmental outcome in term infants with status epilepticus detected with amplitude-integrated electroencephalography | Pediatrics | 120 | 2 | e354-63 | No feeding outcomes reported |
| van Rooij, L. G. M. and Toet, M. C. and Osredkar, D. and van Huffelen, A. C. and Groenendaal, F. and de Vries, L. S. | 2005 | Recovery of amplitude integrated electroencephalographic background patterns within 24 hours of perinatal asphyxia | Archives of disease in childhood. Fetal and neonatal edition | 90 | 3 | F245-51 | No feeding outcomes reported |
| Van Steenis, A. and Cizmeci, M. N. and Groenendaal, F. and Thoresen, M. and Cowan, F. M. and De Vries, L. S. and Steggerda, S. J. | 2024 | Individualized Neuroprognostication in Neonates with Hypoxic-Ischemic Encephalopathy Treated with Hypothermia | Neurology: Clinical Practice | 15 | 1 | e200370 | No analysis of breastfeeding or lactation outcomes |
| Vasiljevic, Brankica and Maglajlic-Djukic, Svjetlana and Gojnic, Miroslava | 2012 | The prognostic value of amplitude-integrated electroencephalography in neonates with hypoxic-ischemic encephalopathy | Vojnosanitetski pregled | 69 | 6 | 492-9 | No feeding outcomes reported |
| Vasiljevic, B. and Maglajlic-Djukic, S. and Stankovic, S. and Lutovac, D. and Gojnic, M. | 2011 | Predictive value of color Doppler neuro-sonography for the development of neurological sequels in newborn infants with hypoxic ischemic encephalopathy | Vojnosanitetski Pregled | 68 | 10 | 825-831 | No feeding outcomes reported |
| Venkateswaran, Sunita and Shevell, Michael I. | 2008 | Comorbidities and clinical determinants of outcome in children with spastic quadriplegic cerebral palsy | Developmental medicine and child neurology | 50 | 3 | 216-22 | No analysis of factors influencing HIE feeding outcomes |
| Wang, Z. and Zhang, D. and Zhang, P. and Zhou, W. and Hu, L. and Wang, L. and Cheng, G. | 2023 | Safety and efficacy of therapeutic hypothermia in neonates with mild hypoxic-ischemic encephalopathy | BMC Pediatrics | 23 | 1 | 530 | No feeding outcomes reported |
| Weeke, L.C. | 2017 | Seizures and the Neonatal Brain |  |  |  |  | No feeding outcomes reported |
| Weeke, L. C. and Boylan, G. B. and Pressler, R. M. and Hallberg, B. and Blennow, M. and Toet, M. C. and Groenendaal, F. and de Vries, L. S. | 2016 | Role of EEG background activity, seizure burden and MRI in predicting neurodevelopmental outcome in full-term infants with hypoxic-ischaemic encephalopathy in the era of therapeutic hypothermia | European Journal of Paediatric Neurology | 20 | 6 | 855-864 | No feeding outcomes reported |
| Weeke, Lauren C. and Vilan, Ana and Toet, Mona C. and van Haastert, Ingrid C. and de Vries, Linda S. and Groenendaal, Floris | 2017 | A Comparison of the Thompson Encephalopathy Score and Amplitude-Integrated Electroencephalography in Infants with Perinatal Asphyxia and Therapeutic Hypothermia | Neonatology | 112 | 1 | 24-29 | No feeding outcomes reported |
| Whyte, H. E. and Taylor, M. J. and Menzies, R. | 1986 | Prognostic utility of visual evoked potentials in term asphyxiated neonates | Pediatric Neurology | 2 | 4 | 220-223 | No feeding outcomes reported |
| Winkler, Ira and Heisinger, Tatjana and Hammerl, Marlene and Huber, Eva and Urbanek, Martina and Kiechl-Kohlendorfer, Ursula and Griesmaier, Elke and Posod, Anna | 2022 | MicroRNA Expression Profiles as Diagnostic and Prognostic Biomarkers of Perinatal Asphyxia and Hypoxic-Ischaemic Encephalopathy | Neonatology | 119 | 2 | 204-213 | No feeding outcomes reported |
| Wu, Yvonne W. and Mathur, Amit M. and Taeun, Chang and McKinstry, Robert C. and Mulkey, Sarah B. and Mayock, Dennis E. and Van Meurs, Krisa P. and Rogers, Elizabeth E. and Gonzalez, Fern and o F. and Comstock, Bryan A. and Juul, S and ra E. and Msall, Michael E. and Bonifacio, Sonia L. and Glass, Hannah C. and Massaro, An N. and Dong, Lawrence and Tan, Katherine W. and Heagerty, Patrick J. and Ballard, Roberta A. | 2016 | High-Dose Erythropoietin and Hypothermia for Hypoxic-Ischemic Encephalopathy: A Phase II Trial | Pediatrics | 137 | 6 | 15-15 | No feeding outcomes reported |
| Xia, Y. Q. and Yang, M. S. and Qian, T. Y. and Zhou, J. Y. and Bai, M. and Luo, S. Q. and Lu, C. G. and Zhu, Y. H. and Wang, L. S. and Qiao, Z. W. | 2024 | Prediction of feeding difficulties in neonates with hypoxic-ischemic encephalopathy using magnetic resonance imaging-derived radiomics features | Pediatric Radiology | 54 | 12 | 2036-2045 | Mixed cohort |
| Yeo, C. L. and Tudehope, D. I. | 1994 | Outcome of resuscitated apparently stillborn infants: A ten year review | Journal of Paediatrics and Child Health | 30 | 2 | 129-133 | No feeding outcomes reported |
| Yokochi, K. | 1997 | Oral motor patterns during feeding in severely physically disabled children | Brain & development | 19 | 8 | 552-5 | No analysis of factors influencing HIE feeding outcomes |
| Yokochi, K. and Fujimoto, S. | 1996 | Magnetic resonance imaging in children with neonatal asphyxia: correlation with developmental sequelae | Acta paediatrica (Oslo, Norway : 1992) | 85 | 1 | 88-95 | Aged 5 years and older |
| Yudkin, P. L. and Johnson, A. and Clover, L. M. and Murphy, K. W. | 1994 | Clustering of perinatal markers of birth asphyxia and outcome at age five years | British journal of obstetrics and gynaecology | 101 | 9 | 774-81 | No feeding outcomes reported |
| Yum, Sook Kyung and Seo, Yu Mi and Kwun, Yoojin and Moon, Cheong-Jun and Youn, Young-Ah and Sung, In Kyung | 2018 | Therapeutic hypothermia in infants with hypoxic-ischemic encephalopathy and reversible persistent pulmonary hypertension: short-term hospital outcomes | The journal of maternal-fetal & neonatal medicine : the official journal of the European Association of Perinatal Medicine, the Federation of Asia and Oceania Perinatal Societies, the International Society of Perinatal Obstetricians | 31 | 23 | 3108-3114 | No analysis of breastfeeding or lactation outcomes |
| Zhang, D. D. and Ding, H. Y. and Liu, L. L. and Hou, X. L. and Sun, G. Y. and Li, L. and Liu, Y. Z. and Zhou, C. L. and Gu, R. L. and Luo, Y. J. | 2013 | The Prognostic Value of Amplitude-Integrated EEG in Full-Term Neonates with Seizures | Plos One | 8 | 11 |  | No feeding outcomes reported |
| Zhou, W. H. and Cheng, G. Q. and Shao, X. M. and Liu, X. Z. and Shan, R. B. and Zhuang, D. Y. and Zhou, C. L. and Du, L. Z. and Cao, Y. and Yang, Q. and Wang, L. S. and China Study, Grp | 2010 | Selective Head Cooling with Mild Systemic Hypothermia after Neonatal Hypoxic-Ischemic Encephalopathy: A Multicenter Randomized Controlled Trial in China | Journal of Pediatrics | 157 | 3 | 367-372 | No feeding outcomes reported |
| Zhou, W. H. and Shao, X. M. and Cao, Y. and Chen, C. and Zhang, X. D. | 2002 | Safety study of hypothermia for treatment of hypoxic-ischemic brain damage in term neonates | Acta Pharmacologica Sinica | 23 |  | 64-68 | No feeding outcomes reported |
| Zubcevic, S. and Heljic, S. and Spahovic, R. and Kalkan, I. and Terzic, S. and Sadikovic, M. | 2014 | Neurodevelopmental outcome following therapeutic hypothermia for perinatal asphyxia | Paediatria Croatica | 58 | 4 | 262-269 | No feeding outcomes reported |
