# Supplementary material for: Factors Influencing Breastfeeding Outcomes Following Neonatal Hypoxic Ischaemic Encephalopathy: A Mixed Methods Systematic Review
Source: J Hum Lact. 2026 Apr 3;42(2):302–14. doi: 10.1177/08903344261426707 (PMC13263470; doi:10.1177/08903344261426707)
Supplement: sj-docx-2-jhl-10.1177_08903344261426707 – Supplemental material for Factors Influencing Breastfeeding Outcomes Following Neonatal Hypoxic Ischaemic Encephalopathy: A Mixed Methods Systematic Review [file sj-docx-2-jhl-10.1177_08903344261426707.docx]

**Electronic Search Strategies**

Ovid MEDLINE(R) and In-Process, In-Data-Review & Other Non-Indexed Citations

1 Child, Preschool

2 limit 1 to (humans)

3 baby.mp.

4 limit 3 to (humans)

5 babies.mp.

6 limit 5 to (humans)

7 neonat*.mp.

8 limit 7 to (humans)

9 newborn.mp.

10 limit 9 to (humans)

11 infant*.mp. or exp Infant/

12 limit 11 to (humans)

13 toddler*.mp.

14 limit 13 to (humans)

15 Pediatrics/

16 limit 15 to (humans)

17 p?ediatric.mp.

18 limit 17 to (humans)

19 2 or 4 or 6 or 8 or 10 or 12 or 14 or 16 or 18

20 exp "Feeding and Eating Disorders"/

21 limit 20 to (humans)

22 feeding methods/ or enteral nutrition/

23 limit 22 to (humans)

24 deglutition/ or eating/

25 limit 24 to (humans)

26 swallowing.mp.

27 limit 26 to (humans)

28 dysphagia.mp.

29 limit 28 to (humans)

30 exp Feeding Behavior/

31 limit 30 to (humans)

32 Deglutition Disorders/

33 limit 32 to (humans)

34 feed*.mp.

35 limit 34 to (humans)

36 gastrostomy.mp. or Gastrostomy/

37 limit 36 to (humans)

38 nasogastric tube.mp.

39 limit 38 to (humans)

40 neurodevelopment* outcome*.mp.

41 limit 40 to (humans)

42 21 or 23 or 25 or 27 or 29 or 31 or 33 or 35 or 37 or 39 or 41

43 hypoxic isch?emic encephalopathy.mp. or Hypoxia-Ischemia, Brain/

44 limit 43 to (humans)

45 asphyxia neonatorum.mp. or Asphyxia Neonatorum/

46 limit 45 to (humans)

47 neonatal asphyxia.mp.

48 limit 47 to (humans)

49 perinatal asphyxia.mp.

50 limit 49 to (humans)

51 birth asphyxia.mp.

52 limit 51 to (humans)

53 newborn hypoxia.mp.

54 limit 53 to (humans)

55 neonatal brain injur*.mp.

56 limit 55 to (humans)

57 44 or 46 or 48 or 50 or 52 or 54 or 56

58 19 and 42 and 57

Embase

1 hypoxic isch?emic encephalopathy.mp. or hypoxic ischemic encephalopathy/

2 limit 1 to (human)

3 asphyxia neonatorum.mp.

4 limit 3 to (human)

5 neonatal asphyxia.mp. or newborn hypoxia/

6 limit 5 to (human)

7 perinatal asphyxia.mp. or perinatal asphyxia/

8 limit 7 to (human)

9 birth asphyxia.mp.

10 limit 9 to (human)

11 neonatal brain injur*.mp.

12 limit 11 to (human)

13 2 or 4 or 6 or 8 or 10 or 12

14 enteric feeding/ or artificial feeding/

15 limit 14 to (human)

16 eating/

17 limit 16 to (human)

18 dysphagia.mp. or dysphagia/

19 limit 18 to (human)

20 swallowing.mp. or swallowing/

21 limit 20 to (human)

22 deglutition.mp.

23 limit 22 to (human)

24 feed*.mp.

25 limit 24 to (human)

26 feeding behavior/

27 limit 26 to (human)

28 gastrostomy/ or gastrostomy.mp.

29 limit 28 to (human)

30 nasogastric tube.mp. or nasogastric tube/

31 limit 30 to (human)

32 exp feeding disorder/

33 limit 32 to (human)

34 neurodevelopment* outcome*.mp.

35 limit 34 to (human)

36 15 or 17 or 19 or 21 or 23 or 25 or 27 or 29 or 31 or 33 or 35

37 preschool child/

38 limit 37 to (human)

39 baby.mp. or baby/

40 limit 39 to (human)

41 babies.mp.

42 limit 41 to (human)

43 neonat*.mp.

44 limit 43 to (human)

45 newborn.mp. or newborn/

46 limit 45 to (human)

47 infant*.mp.

48 limit 47 to (human)

49 high risk infant/ or hospitalized infant/

50 limit 49 to (human)

51 toddler/ or toddler*.mp.

52 limit 51 to (human)

53 p*ediatric.mp. or pediatric patient/

54 limit 53 to (human)

55 38 or 40 or 42 or 44 or 46 or 48 or 50 or 52 or 54

56 13 and 36 and 55

CINAHL

(((MW child* OR TI child* OR AB child*) OR (MW baby OR TI baby OR AB baby) OR (MW babies OR TI babies OR AB babies)) OR ((MW neonat* OR TI neonat* OR AB neonat*) OR (MW newborn OR TI newborn OR AB newborn) OR (MW infant* OR TI infant* OR AB infant*)) OR ((MW toddler* OR TI toddler* OR AB toddler*) OR (MW p#ediatric* OR TI p#ediatric* OR AB p#ediatric*)))

AND

(((MW feed* OR TI feed* OR AB feed*) OR (MW enteral nutrition OR TI enteral nutrition OR AB enteral nutrition) OR (MW deglutition OR TI deglutition OR AB deglutition) OR (MW eating OR TI eating OR AB eating) OR (MW swallowing OR TI swallowing OR AB swallowing) OR (MW dysphagia OR TI dysphagia OR AB dysphagia) OR (MW gastrostomy OR TI gastrostomy OR AB gastrostomy)) OR ((MW nasogastric tube OR TI nasogastric tube OR AB nasogastric tube) OR (MW neurodevelopment* outcome* OR TI neurodevelopment* outcome* OR AB neurodevelopment* outcome*)))

AND

(((MW hypoxic isch#emic encephalopathy OR TI hypoxic isch#emic encephalopathy OR AB hypoxic isch#emic encephalopathy) OR (MW hypoxia isch#emia, brain OR TI hypoxia isch#emia, brain OR AB hypoxia isch#emia, brain) OR (MW asphyxia neonatorum OR TI asphyxia neonatorum OR AB asphyxia neonatorum) OR (MW neonatal asphyxia OR TI neonatal asphyxia OR AB neonatal) OR (MW perinatal asphyxia OR TI perinatal asphyxia OR AB perinatal asphyxia) OR (MW birth asphyxia OR TI birth asphyxia OR AB birth asphyxia)) OR ((MW newborn hypoxia OR TI newborn hypoxia OR AB newborn hypoxia) OR (MW neonatal brain injur* OR TI neonatal brain injur* OR AB neonatal brain injur*)))

APA PsycInfo

1 Preschool Students/

2 limit 1 to (human)

3 baby.mp.

4 limit 3 to (human)

5 babies.mp.

6 limit 5 to (human)

7 neonat*.mp.

8 limit 7 to (human)

9 newborn.mp.

10 limit 9 to (human)

11 infant*.mp.

12 limit 11 to (human)

13 toddler*.mp.

14 limit 13 to (human)

15 exp Pediatrics/ or p?ediatric*.mp.

16 limit 15 to (human)

17 2 or 4 or 6 or 8 or 10 or 12 or 14 or 16

18 exp Feeding Disorders/

19 limit 18 to (human)

20 enteral nutrition.mp.

21 limit 20 to (human)

22 feed*.mp.

23 limit 22 to (human)

24 exp Dysphagia/ or Swallowing/ or deglutition.mp.

25 limit 24 to (human)

26 exp Eating Behavior/

27 limit 26 to (human)

28 gastrostomy.mp.

29 limit 28 to (human)

30 nasogastric tube.mp.

31 limit 30 to (human)

32 neurodevelopment* outcome*.mp.

33 limit 32 to (human)

34 19 or 21 or 23 or 25 or 27 or 29 or 31 or 33

35 hypoxic isch?emic encephalopathy.mp.

36 limit 35 to (human)

37 hypoxia-isch?emia, brain.mp.

38 limit 37 to (human)

39 asphyxia neonatorum.mp.

40 limit 39 to (human)

41 neonatal asphyxia.mp.

42 limit 41 to (human)

43 perinatal asphyxia.mp.

44 limit 43 to (human)

45 birth asphyxia.mp.

46 limit 45 to (human)

47 newborn hypoxia.mp.

48 limit 47 to (human)

49 neonatal brain injur*.mp.

50 limit 49 to (human)

51 36 or 38 or 40 or 42 or 44 or 46 or 48 or 50

52 17 and 34 and 51

Scopus

("hypoxic isch#emic encephalopathy" OR "asphyxia neonatorum" OR "neonatal asphyxia" OR "perinatal asphyxia" OR "birth asphyxia" OR "newborn hypoxia" OR "neonatal brain injur*") AND (feed* OR "enteral nutrition" OR deglutition OR eating OR swallowing OR dysphagia OR gastrostomy OR "nasogastric tube" OR "neurodevelopment* outcome*") AND ( child* OR baby OR babies OR neonat* OR newborn OR infant* OR toddler* OR p#ediatric*)

Web of Science

("hypoxic isch$emic encephalopathy" OR "asphyxia neonatorum" OR "neonatal asphyxia" OR "perinatal asphyxia" OR "birth asphyxia" OR "newborn hypoxia" OR "neonatal brain injur*") AND (feed* OR "enteral nutrition" OR deglutition OR eating OR swallowing OR dysphagia OR gastrostomy OR "nasogastric tube" OR "neurodevelopment* outcome*") AND ( child* OR baby OR babies OR neonat* OR newborn OR infant* OR toddler* OR p$ediatric*)

Cochrane Database of Systematic Reviews

("hypoxic isch#emic encephalopathy" OR "asphyxia neonatorum" OR "neonatal asphyxia" OR "perinatal asphyxia" OR "birth asphyxia" OR "newborn hypoxia" OR "neonatal brain injur*") AND (feed* OR "enteral nutrition" OR deglutition OR eating OR swallowing OR dysphagia OR gastrostomy OR "nasogastric tube" OR "neurodevelopment* outcome*") AND ( child* OR baby OR babies OR neonat* OR newborn OR infant* OR toddler* OR p#ediatric*)

Open Access Theses and Dissertations

("hypoxic isch#emic encephalopathy" OR "asphyxia neonatorum" OR "neonatal asphyxia" OR "perinatal asphyxia" OR "birth asphyxia" OR "newborn hypoxia" OR "neonatal brain injur*") AND (feed* OR "enteral nutrition" OR deglutition OR eating OR swallowing OR dysphagia OR gastrostomy OR "nasogastric tube" OR "neurodevelopment* outcome*") AND ( child* OR baby OR babies OR neonat* OR newborn OR infant* OR toddler* OR p#ediatric*)

EThOS

("hypoxic isch$emic encephalopathy" OR "asphyxia neonatorum" OR "neonatal asphyxia" OR "perinatal asphyxia" OR "birth asphyxia" OR "newborn hypoxia" OR "neonatal brain injur*") AND (feed* OR "enteral nutrition" OR deglutition OR eating OR swallowing OR dysphagia OR gastrostomy OR "nasogastric tube" OR "neurodevelopment* outcome*") AND ( child* OR baby OR babies OR neonat* OR newborn OR infant* OR toddler* OR p$ediatric*)

Networked Digital Library of Theses and Dissertations (Global ETD search)

*site not longer functioning at 2025 search update

("hypoxic isch#emic encephalopathy" OR "asphyxia neonatorum" OR "neonatal asphyxia" OR "perinatal asphyxia" OR "birth asphyxia" OR "newborn hypoxia" OR "neonatal brain injur*") AND (feed* OR "enteral nutrition" OR deglutition OR eating OR swallowing OR dysphagia OR gastrostomy OR "nasogastric tube" OR "neurodevelopment* outcome*") AND ( child* OR baby OR babies OR neonat* OR newborn OR infant* OR toddler* OR p#ediatric*)

ProQuest Dissertation Express

("hypoxic isch*emic encephalopathy" OR "asphyxia neonatorum" OR "neonatal asphyxia" OR "perinatal asphyxia" OR "birth asphyxia" OR "newborn hypoxia" OR "neonatal brain injur*") AND (feed* OR "enteral nutrition" OR deglutition OR eating OR swallowing OR dysphagia OR gastrostomy OR "nasogastric tube" OR "neurodevelopment* outcome*") AND (child* OR baby OR babies OR neonat* OR newborn OR infant* OR toddler* OR p*ediatric*)
